# Developing clinical prediction models: a step-by-step guide

**DOI:** 10.1136/bmj-2023-078276

**Published:** 2024-09-03

**Authors:** Orestis Efthimiou, Michael Seo, Konstantina Chalkou, Thomas Debray, Matthias Egger, Georgia Salanti

**Affiliations:** 1 Institute of Primary Health Care (BIHAM), University of Bern, Bern, Switzerland; 2 Institute of Social and Preventive Medicine (ISPM), University of Bern, Bern, Switzerland; 3 Department of Clinical Research, University of Bern, Bern, Switzerland; 4 Smart Data Analysis and Statistics B V, Utrecht, The Netherlands; 5 Population Health Sciences, Bristol Medical School, University of Bristol, Bristol, UK

## Abstract

Predicting future outcomes of patients is essential to clinical practice, with many prediction models published each year. Empirical evidence suggests that published studies often have severe methodological limitations, which undermine their usefulness. This article presents a step-by-step guide to help researchers develop and evaluate a clinical prediction model. The guide covers best practices in defining the aim and users, selecting data sources, addressing missing data, exploring alternative modelling options, and assessing model performance. The steps are illustrated using an example from relapsing-remitting multiple sclerosis. Comprehensive R code is also provided.

Clinical prediction models aim to forecast future health outcomes given a set of baseline predictors to facilitate medical decision making and improve people’s health outcomes.^1^ Prediction models are becoming increasingly popular, with many new ones published each year. For example, a review of prediction models identified 263 prediction models in obstetrics alone^2^; another review found 606 models related to covid-19.^3^ Interest in predicting health outcomes has been heightened by the increasing availability of big data,^4^ which has also led to the uptake of machine learning methods for prognostic research in medicine.^5^ ^6^

Several resources are available to support prognostic research. The PROGRESS (prognosis research strategy) framework provides detailed guidance on different types of prognostic research.^7^ ^8^ ^9^ The TRIPOD (transparent reporting of a multivariable prediction model for individual prognosis or diagnosis) statement gives recommendations for reporting and has recently been extended to address prediction model research in clustered datasets.^10^ ^11^ ^12^ ^13^ ^14^ PROBAST (prediction model risk-of-bias assessment tool) provides a structured way to assess the risk of bias in a prediction modelling study.^15^ Several papers further outline good practices and provide software code.^16^ ^17^ ^18^

Despite these resources, published prediction modelling studies often have severe methodological limitations. For instance, a review of prediction models for cardiovascular disease identified 363 models^19^; the authors concluded that “the usefulness of most of the models remains unclear owing to methodological shortcomings, incomplete presentation, and lack of external validation and model impact studies.” Another review of 308 prediction models in psychiatry found that most were at high risk of bias.^20^ Many biases well known in clinical and epidemiological research also apply to prediction model studies, including inconsistent definitions and measurements of predictors and outcomes or lack of blinding. Some biases are particularly pertinent to prediction modelling; for example, overfitting—estimating many model parameters from few data points—can lead to overestimating the model's performance.^15^

This article provides a step-by-step guide for researchers interested in clinical prediction modelling. Based on a scoping review of the literature and discussions in our group, we identified 13 steps. We aim to provide an overview to help numerically minded clinicians, clinical epidemiologists, and statisticians navigate the field. We introduce key concepts and provide references to further reading for each step. We discuss issues related to model inception, provide practical recommendations about selecting predictors, outline sample size considerations, cover aspects of model development, such as handling missing data and assessing performance, and discuss methods for evaluating the model’s clinical usefulness. The concepts we describe and the steps we propose largely apply to statistical and machine learning models. An appendix with code in R accompanies the paper. Although several issues discussed here are also relevant to diagnostic research^21^ (which is related but has subtle differences with prediction modelling) and models on predicting treatment effects,^22^ ^23^ our focus is primarily on methods for predicting a future health outcome. We illustrate the proposed procedure using an example of a prediction model for relapse in relapsing-remitting multiple sclerosis. The glossary in table 1 summarises the essential concepts and terms used.

**Table 1:** Glossary of key terms and concepts used in prediction modelling

Term	Explanation
Predictors (also called covariates, baseline variables, or features)	Set of patient level characteristics on which prediction of a future outcome will be based
Training (also called development) set or dataset	Data used to develop a model
Testing set or dataset	Data used to test the model
Discrimination	For continuous outcomes, discrimination relates to the capacity of the model to rank patients concerning their outcomes. For a perfectly discriminating model, and for two randomly chosen patients, the patient with the higher predicted outcome will also have the higher observed outcome. For a binary or survival outcome, discrimination relates to the capacity of a model to split the patient into groups at different risk. For a perfectly discriminating model of a binary outcome, the patient with higher predicted probability will have higher true risk of an event. For a perfectly discriminating model of a survival outcome, the patient with the higher predicted survival probability will also have longer survival time
Calibration	Calibration refers to the agreement between predicted and observed outcomes. Three increasingly stringent methods for assessing calibration can be used: mean calibration (calibration in the large) is assessed by comparing the average predicted outcomes or average predicted risks with the average observed outcomes or risks. For example, for a model with good mean calibration, the mean predicted outcome was 2.4 (in some continuous scale) and the mean observed outcome was also 2.4. Calibration can also be assessed across predictions by fitting a calibration line on observations versus predictions; this summarises calibration with two numbers, the intercept and slope of the calibration line. Alternatively, a smooth calibration curve can be fit to also assess calibration locally; this might show, for example, that a model for a binary outcome is well calibrated for predicted risks of 20% or less, but overestimates risks higher than 20%
Underfitting	When a model is not complex enough and cannot capture patterns in the data well. Underfitting leads to reduced model performance (but no optimism)
Overfitting	When a model performs very well in the training dataset but fails to predict in new data; particularly relevant when the sample size is small, and the model has many parameters. Overfitting leads to optimism
Apparent performance	Performance of a prediction model when the same dataset is used for developing and assessing performance of the model (ie, training set=testing set). Apparent performance is prone to optimism, especially in case of overfitting. Therefore, apparent performance might be misleading
True performance	Performance of a prediction model when applied to the general population of interest
Optimism	Difference between apparent and true model performance. If a model is overfitting, optimism might be large
Bias-variance trade-off	Relates to the trade-off between having a simple, underfitting prediction model versus a complex, overfitting one. Prediction error in new data is a function of bias and variance. In this context, bias relates to the error of the model owing to simplifying assumptions used. Variance refers to the variability of predictions made. Simple models have high bias, low variance; the opposite holds for complex models. Increasing model complexity decreases bias and increases variance. The aim is to develop a model with minimum prediction error in new patients; such a model sits on the sweet spot of the bias-variance trade-off curve where the model is not too simple, or too complex
Internal validation	Methods for obtaining an honest (ie, not optimistic) assessment of the performance of prediction models using the data it was developed with
Optimism corrected performance	Predictive performance of model after correcting for optimism using internal validation
Split sample internal validation	Internal validation method where the sample is randomly split into two parts; one part is used for developing the model, the other for assessing its performance
k-fold cross validation	Data are split in k folds; the model is developed in k−1 folds and tested in the left out fold; procedure cycles through all folds. The method can be used for internal validation; it is also sometimes used for model development, for example, to determine the value of tuning parameters in penalisation (shrinkage) models
Temporal validation	Internal validation method where data are split according to the time of patient enrolment. Model is developed in patients enrolled earlier and is tested in patients enrolled later in time
Bootstrapping	Process of creating samples mimicking the original sample. Bootstrap samples are drawn with replacement from the original sample. Bootstrapping can be used for internal validation
Internal-external validation	Method for validating a prediction model using a clustering variable in the dataset. All clusters but one are used to develop the model; the model is subsequently tested in the left out cluster. The procedure cycles through all clusters and performance measures are summarised at the end
External validation	Evaluation of model’s performance in new data—ie, data not used for training the model. External validation should ideally be performed by independent researchers who are not involved in model development. The more diverse the setting and population of the external validation, the more we learn about model generalisability and transportability
Penalisation (also called regularisation, related to shrinkage)	General method for reducing model complexity to obtain a model with better predictions. In regression models, coefficients are shrunk, leading to less complex models. Penalisation is controlled by one or more penalty parameters embedded in the model. The amount of penalisation ideally needed is one that brings the model to the sweet spot of the bias-variance curve—ie, where the model is as complex as it should be, but no more than that
LASSO, ridge regression, elastic net	Penalised estimation methods for regression models. LASSO (least absolute shrinkage and selection operator) and elastic net perform variable selection
Reproducibility	Estimated model performance can be reproduced in new sample from the same population or setting as the one used to develop the model
Transportability	Transportability refers to the ability of the model to produce accurate predictions in new patients drawn from a different but related population or setting
Generalisability	Generalisability encompasses model’s reproducibility and transportability

**Summary points**

Many prediction models are published each year, but they often have methodological shortcomings that limit their internal validity and applicability. A 13 step guide has been developed to help healthcare professionals and researchers develop and validate prediction models, avoiding common pitfalls
In the first step, the objective of the prediction model should be defined, including the target population, the outcome to be predicted, the healthcare setting where the model will be used, the intended users, and the decisions the model will inform
Prediction modelling requires a collaborative and interdisciplinary effort within a team that ideally includes clinicians with content expertise, methodologists, users, and people with lived experiences
Common pitfalls include inappropriate categorising of continuous outcomes or predictors, data driven cut-off points, univariable selection methods, overfitting, and lack of attention to missing data and a sound assessment of performance and clinical benefit

## Step 1: Define aims, create a team, review literature, start writing a protocol

### Defining aims

We should start by clearly defining the purpose of the envisaged prediction model. In particular, it is important to clearly determine the following:

- The target population—for whom should the model predict? For example, people with HIV in South Africa; people with a history of diabetes; postmenopausal women in western Europe.
- The health outcome of interest—what is the endpoint that needs to be predicted? For example, AIDS, overall survival, progression free survival, a particular adverse event.
- The healthcare setting—how will the model be used? For example, the model might be used in primary care or be implemented in a clinical decision support system in tertiary care.
- The user—who is going to use the model? For example, primary care physicians, secondary care physicians, patients, researchers.
- The clinical decisions that the model will inform—how will model predictions be used in the clinical decision making process? For example, a model might be used to identify patients for further diagnostic investigation, to decide on treatment strategies, or to inform a range of personal decisions.^24^

Answers to these questions should guide the subsequent steps; they will inform various issues, such as what predictors to include in the model, what data to use for developing and validating the model, and how to assess its clinical usefulness.

### Creating a team

When developing a prediction model for clinical use, assembling a group with expertise in the specific medical field, the statistical methodology, and the source data are highly advisable. Including users—that is, clinicians who might use the model and people with lived experiences—is also beneficial. Depending on the model's complexity, it might be necessary to involve software developers at later stages of the project; that is, developing a web application for users to make predictions.

### Reviewing the literature

Identifying relevant published prediction models and studies on important risk factors is crucial and can be achieved through a scoping review. Discussing the review's findings with clinicians will help us to understand established predictors and the limitations of existing models. The literature review might also provide information on interactions between predictors, nonlinear associations between predictors and outcomes, reasons for missing data, and the expected distribution of predictors in the target population. In some situations, performing a systematic review might be helpful. Specific guidance on systematic reviews of prediction models has been published.^25^ ^26^ ^27^

### Protocol

A study protocol should guide subsequent steps. The protocol can be made publicly available in an open access journal or as a preprint in an online repository (eg, www.medrxiv.org or https://osf.io/). In addition to the steps discussed here, the TRIPOD statement^10^ ^14^ and the PROBAST tool^15^ might be helpful resources when writing the protocol.

## Step 2: Choose between developing a new model or updating an existing one

Depending on the specific field, the literature review might show that relevant prediction models already exist. Suppose an existing model has a low risk of bias (according to PROBAST^15^) and applies to the research question. In that case, assessing its validity for the intended setting might be more appropriate than developing a new model. This approach is known as external validation (table 1). Depending on the validation results, we might decide to update and adapt the model to the population and setting of intended use. Common strategies for updating a prediction model include recalibration (eg, adjustment of the intercept term in a regression model), revision (ie, re-estimation of some model parameters), and extension (ie, addition of new predictors).^28^ ^29^ Although updating strategies have mainly been described for regression models, they can also be applied to machine learning. For example, a random forest model was used to predict whether patients with stroke would experience full recovery within 90 days of the event. When tested on an external dataset, the model needed recalibration, which was performed by fitting logistic regression models to the predictions from the random forest.^30^ Prediction models for imaging data are often developed by fine tuning previously trained neural networks using a process known as transfer learning.^31^

Further guidance on external validation and model updating is available elsewhere,^32^ ^33^ ^34^ ^35^ ^36^ including sample size considerations for external validation.^37^ In the following steps, we focus on developing a new model; we briefly revisit external validation in step 9.

## Step 3: Define the outcome measure

An outcome can be defined and measured in many ways. For example, postoperative mortality can be measured as a binary outcome at 30 days, at 60 days, or using survival time. Using time-to-event instead of binary variables is good practice; a prediction model for time-to-event can better handle people who were followed up for a limited time and did not experience the outcome of interest. Moreover, time-to-event data provide richer information (eg, the survival probability at any time point) than a binary outcome at one time point only. Similarly, we can analyse a continuous health outcome using a continuous scale or after dichotomising or categorising. For example, a continuous depression score at week 8 after starting drug treatment could be dichotomised as remission or non-remission. Categorising a continuous outcome leads to loss of information.^38^ ^39^ ^40^ Moreover, the selection of thresholds for categorisation is often arbitrary, lacking biological justification. In some cases, thresholds are chosen after exploring various cut-off points and opting for those that fit the data best or yield statistically significant results. This data driven approach could lead to reduced performance in new data.^38^

## Step 4: Identify candidate predictors and specify measurement methods

### Candidate predictors

We should identify potential predictors based on the literature review and expert knowledge (step 1). Like the outcomes of interest, they should ideally be objectively defined and measured using an established, reliable method. Understanding the biological pathways that might underpin associations between predictors and the outcome is key. Predictors with proven or suspected causal relationships with the outcome should be prioritised for inclusion; this approach might increase the model's generalisability. On the other hand, the absence of a causal relationship should not a priori exclude potential predictors. Predictors not causally related to the outcome but strongly associated with it might still contribute to model performance, although they might generalise less well to different settings than causal factors. Further, we must include only baseline predictors; that is, information available when making a prognosis. Dichotomising or categorising continuous predictors reduces information and diminishes statistical power and should be avoided.^41^ ^42^ Similarly to categorising outcomes, we advise against making data driven, post hoc decisions after testing several categorisation thresholds for predictors. In other words, we should not choose the categories of a continuous outcome based solely on the associated model performance.

### Thinking about the user of the prediction model

It is crucial to consider the model's intended use (defined in step 1) and the availability of data. What variables are routinely measured in clinical practice and are available in the database? What are the costs and practical issues related to their measurement, including the degree of invasiveness?^43^ For example, the veterans ageing cohort study index (VACS index 2.0) predicts all cause mortality in people with HIV.^44^ However, some of its predictors, such as the liver fibrosis index (FIB-4), will not be available in routine practice in many settings with a high prevalence of HIV infection. Similarly, a systematic review of prognostic models for multiple sclerosis found that 44 of 75 models (59%) included predictors unlikely to be measured in primary care or standard hospital settings.^45^

## Step 5: Collect and examine data

### Data collection

Ideally, prediction models are developed using individual participant data from prospective cohort studies designed for this purpose.^1^ In practice, developing prediction models using existing data from cohort studies or other data not collected explicitly for this purpose is much more common. Data from randomised clinical trials can also be used. The quality of trial data will generally be high, but models could have limited generalisability because trial participants might not represent the patients seen in clinical practice. For example, a study found only about 20% of people who have schizophrenia spectrum disorders would be eligible for inclusion in a typical randomised clinical trial. Patients who are ineligible had a higher risk of hospital admission with psychosis than those who are eligible.^46^ Therefore, a prediction model based on trial data might underestimate the real world risk of hospital admissions. Registry data offer a simple, low cost alternative; their main advantage is the relatively large sample size and representativeness. However, drawbacks relate to data limitations such as inadequate data on relevant predictors or outcomes, and variability in the timing of measurements.^47^

### Data errors

Before fitting the model, addressing potential misclassification or measurement errors in predictors and outcomes is crucial. This involves considering the nature of the variables collected and the methods used for measurement or classification. For example, predictors such as physical activity or dietary intake are prone to various sources of measurement error.^48^ The extent of these errors can vary across settings, for example, because of differences in the measurement method used. This means that the model's predictive performance and potential usefulness could be reduced.^49^ If the risk of measurement error is considered high, we might consider alternative outcome measures or exclude less important, imprecisely measured predictors from the list created in step 4. In particular, if systematic errors in the dataset do not mirror those encountered in clinical practice, the model’s calibration might be poor. While methods for correcting measurement errors have been proposed, they typically require additional data and assumptions.^49^

### Variable distributions and missing data

After examining their distribution in the dataset, excluding predictors with limited variation is advisable because they will contribute little. For example, if the ages range from 25 to 45 years and the outcomes are not expected to change much within this range, we should remove age from the list of predictors. Similarly, a binary predictor might be present in only a few people. In such cases, we might consider removing it from the model unless there is previous evidence that this is a strong predictor.^47^ More complications arise when a variable with low prevalence is known to have meaningful predictive value. For example, a rare genetic mutation could be strongly associated with the outcome. The mutation could be omitted from the model because its effect is difficult to estimate accurately. Alternatively, the few people with the mutation could be excluded, making the model applicable only to people without it.^47^ Another issue is incomplete data on predictors and outcomes for some participants. Depending on the prevalence of missing data, we might want to modify the outcome or exclude certain candidate predictors. For example, we might omit a predictor with many missing values, especially if there is little evidence of its predictive power and imputing the missing data is challenging (step 7); that is, when the missing values cannot be reliably predicted using the observed data. Conversely, if the missing information can be imputed, we might decide to retain the variable, particularly when there is existing evidence that the predictor is important.

## Step 6: Consider sample size

### General considerations about sample size

A very simple model or a model based on covariates that are not associated with the outcome will perform poorly in the data used to develop it and in new data; this scenario is called underfitting. Conversely, a model with too many predictors developed in a small dataset (overfitting) could perform well in this particular dataset but fail to predict accurately in new data. In practice, overfitting is more common than underfitting because datasets are often small and have few events, and there is the temptation to create models with the best (apparent) performance. Therefore, we must ensure the data are sufficient to develop a robust model that includes the relevant predictors.

### Calculating sample size requirements for a specific model

Riley and colleagues^50^ provide helpful guidance and code^51^ ^52^ on sample size calculations. Users need to specify the overall risk (for binary outcomes) or mean outcome value (for continuous outcomes) in the target population, the number of model parameters, and a measure of expected model performance (eg, the coefficient of determination, R^2^). Note that the number of parameters can be larger than the number of predictors. For example, we need two parameters when using a restricted cubic spline with three knots to model a nonlinear association of age with the outcome. The sample size calculated this way is the minimum for a standard statistical model. The sample size must be several times larger if we want to use machine learning models.^53^ Sample size calculations for such models are considerably more complex and might require simulations.^54^

### Calculating number of model parameters for fixed sample size

Suppose the sample size is fixed or based on an existing study, as is often the case. Then, we should perform sample size calculations to identify the maximum number of parameters we can include in the model. A structured way to guide model development can be summarised as follows:

- Calculate the maximum number of parameters that can be included in the model given the available sample size.
- Use the available parameters sequentially by including predictors from the list, starting from the ones that are perceived to be more important.^55^
- Note that additional parameters will be needed for including nonlinear terms or interactions among the predictors in the list.

## Step 7: Deal with missing data

### General considerations on missing data

After removing predictors or outcomes with many missing values, as outlined in step 5, we might still need to address missing values in the retained data. Relying only on complete cases for model development—that is, participants with data for all variables—can dramatically reduce the sample size. To mitigate the loss of valuable information during model development and evaluation, researchers should consider imputing missing data.

### Imputation of missing data

Multiple imputation is the approach usually recommended to handle missing data during model development, and appropriately accounts for missing data uncertainty.^56^ Several versions of the original dataset are created, each with missing values imputed using an imputation model. The imputation model should be the same (in terms of predictors included, their transformations and interactions) as the final model we will use to make predictions. Additionally, the imputation model might involve auxiliary variables associated with missing data, which can enhance the effectiveness of the imputations. Once we have created the imputed datasets, we must decide whether to include participants with imputed outcomes in the model development. If no auxiliary variables were used in the imputations, people with imputed outcomes can be removed, and the model can be developed based only on people with observed outcomes.^57^ However, if imputation incorporates auxiliary variables, including those with imputed outcomes in the model development is advisable.^58^ A simpler alternative to multiple imputation is single imputation when each missing value is imputed only once using a regression model. Sisk and colleagues showed that single imputation can perform well, although multiple imputation tends to be more consistent and stable.^59^

In step 4, we made the point that a model should include predictors that will be available in practice. However, we might want to make the model available even when some predictors are missing, for example, when using the model in a lower level of care. For example, the QRisk3 tool for predicting cardiovascular disease can be used even if the general practitioner does not enter information on blood pressure variability (the standard deviation of repeated readings).^60^ When anticipating missing data during use in clinical practice, we can impute data during the development and implementation phases. In this case, single imputation can be used during model development and model use.^59^

Ιmputation methods are not a panacea and might fail, typically when the tendency of the outcome to be missing correlates with the outcome itself. For example, patients receiving a new treatment might be more likely to miss follow-up visits if the treatment was successful, leading to missing data. Developing a prediction model in such cases requires additional modelling efforts^61^ that are beyond the scope of this tutorial.

## Step 8: Fit the prediction models

### Modelling strategies

The strategies for model development should be specified in the protocol (step 5). Linear regression for continuous outcomes, logistic regression for binary outcomes, and Cox or simple parametric models for survival outcomes are the usual starting points in modelling. If the sample size is large enough (see step 6), models can include nonlinear terms for continuous predictors or interactions between predictors. More advanced modelling strategies, such as machine learning models (eg, random forests, support vector machines, boosting methods, neural networks, etc), can also be used.^62^ ^63^ These strategies might add value if there are strong nonlinearities and interactions between predictors, although they are not immune to biases.^64^ As discussed under step 10, a final strategy needs to be selected if several modelling strategies are explored.

### Dealing with competing events

When predicting binary or time-to-event outcomes, we should consider whether there are relevant competing events. This situation occurs when several possible outcomes exist, but a person can only experience one event. For example, when predicting death from breast cancer, death from another cause is a competing event. In this case, and especially whenever competing events are common, we should use a competing risks model for the analysis, such as a cause specific Cox regression model.^65^ A simpler approach would be to analyse a composite outcome.

### Data driven variable selection methods

We advise against univariable selection methods—that is, methods that test each predictor separately and retain only statistically significant predictors. These methods do not consider the association between predictors and could lead to loss of valuable information.^55^ ^66^ Stepwise methods for variable selection (eg, forward, backwards, or bidirectional variable selection) are commonly used. Again, they are not recommended because they might lead to bias in estimation and worse predictive performance.^55^ ^67^ ^68^ If variable selection is desirable—for instance, to simplify the implementation of the model by further reducing the number of predetermined predictors—more suitable methods can be used as described below.

### Model estimation

**Fig 1:**
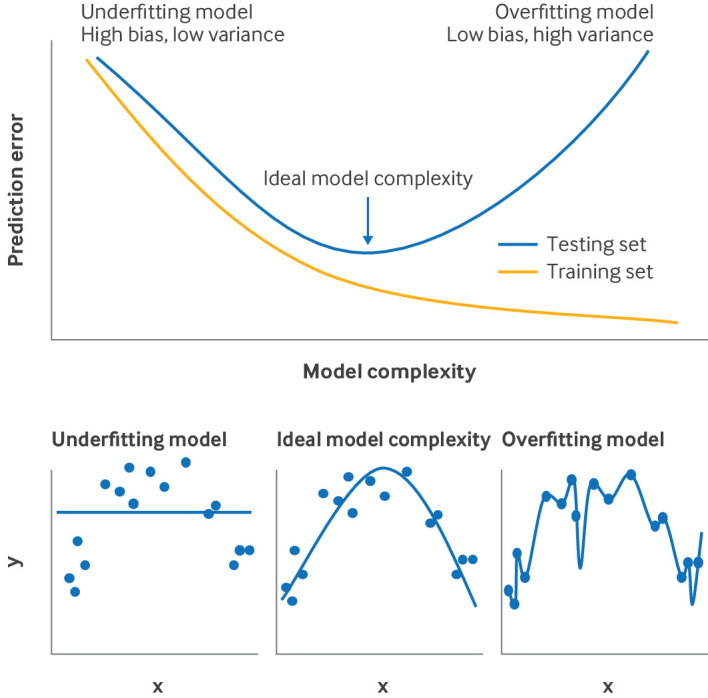
Upper panel: graphical illustration of bias-variance trade-off. The training set is used to develop a model; the testing set is used to test it. A simple, underfitting model leads to high prediction error in training and testing sets. By increasing model complexity, the training set error can be lowered to zero. However, the testing set error (which needs to be minimised) only reduces to a point and then increases as complexity increases. The ideal model complexity is one that minimises the testing set error. An overfitting model might appear to perform well in the training set but might still be worthless—ie, overfitting leads to optimism. Lower three panels: fictional example of three prediction models (lines) developed using a dataset (points). x, y: single continuous predictor and outcome, respectively. The underfitting model has large training error and will also have large testing error; the overfitting model performs perfectly in the development set (ie, zero training error) but will perform poorly in new data (large testing error). The ideal model complexity will perform better than the other two in new data

Adding penalty terms to the model (a procedure called penalisation, regularisation, or shrinkage; see table 1) is recommended to control the complexity of the model and prevent overfitting.^69^ ^70^ ^71^ Penalisation methods such as ridge, LASSO (least absolute shrinkage and selection operator), and elastic net generally lead to smaller absolute values of the coefficients—that is, they shrink coefficients towards zero—compared with maximum likelihood estimation.^72^ LASSO and elastic net can be used for variable selection (similar to the methods described above). These models might exclude some predictors by setting their coefficients to zero, leading to a more interpretable and simpler model. Machine learning methods typically also have penalisation embedded. Penalisation is closely related to the bias-variance trade-off depicted in figure 1, and is a method aiming to bring the model closer to the sweet spot of the bias-variance trade-off curve, where model performance in new data is maximised (note that the figure does not include a description of the double descent phenomenon).^73^ Although penalisation methods have advantages, they do not solve all the problems associated with small sample sizes. While these methods typically are superior to standard estimation techniques, they can be unstable in small datasets. Moreover, their application does not ensure improved predictive performance.^74^ ^75^

### Treating multiply imputed data in model development

If multiple imputation was used, we must apply each modelling strategy to every imputed dataset. Consequently, if there are m imputed datasets, m different models will be developed for each modelling strategy. When predicting outcomes, these m models need to be combined. There are two methods to achieve this. The first method uses Rubin’s rule,^76^ which is suitable for simple regression models. The estimated parameters from the m models are averaged, resulting in a final set of parameters, which can then be used to predict the outcome for a new person. However, this method is not straightforward for model selection strategies (eg, LASSO) because the m fitted models might have selected different sets of parameters. As a result, combining them becomes more complex.^77^ ^78^ Rubin’s rule might not apply to machine learning methods because the m models could have different architectures. Another method for combining the m models is to use them to make predictions for the new person and then average these m predictions,^79^ a procedure conceptually similar to stacking in machine learning.

## Step 9: Assess the performance of prediction models

### General concepts in assessing model performance

We assess the predictive performance of the modelling strategies explored in step 8. Specifically, we contrast predictions with observed outcomes for people in a dataset to calculate performance measures. For continuous outcomes like blood pressure this is straightforward: observed outcomes can be directly compared with predictions because they are on the same scale. When dealing with binary or survival outcomes, the situation becomes more complex. In these cases, prediction models might give the probability of an event occurring for each individual while observed outcomes are binary (event or no event) or involve time-to-event data with censoring. Consequently, more advanced methods are required.

### Dimensions of prediction performance

Prediction performance has two dimensions, and it is essential to assess them both, particularly for binary and survival outcomes (see glossary in table 1).

- Discrimination—for continuous outcomes, discrimination refers to the model’s ability to distinguish between patients with different outcomes: good discrimination means that patients with higher predicted values also had higher observed outcome values. For binary outcomes, good discrimination means that the model separates people at high risk from those at low risk. For time-to-event outcomes, discrimination refers to the ability of the model to rank patients according to their survival; that is, patients predicted to survive longer survived longer.
- Calibration relates to the agreement between observed and predicted outcome values.^80^ ^81^ For continuous outcomes, good calibration means that predicted values do not systematically overestimate or underestimate observed values. For binary and survival outcomes, good calibration means the model does not overestimate or underestimate risks.

Discrimination and calibration are essential when evaluating prediction models. A model can have good discrimination by accurately distinguishing between risk levels, but still have poor calibration owing to a mismatch between predicted and observed probabilities. Moreover, a well calibrated model might have poor discrimination. Thus, a robust prediction model should have good discrimination and calibration. Box 1 outlines measures for assessing model performance.

Box 1 **Measures of performance of prediction models for different types of outcomes**

**Continuous outcomes**

Predicted and observed outcomes can be compared through mean bias, mean squared error, and the coefficient of determination, R^2^, to measure overall performance—ie, combining calibration and discrimination. For discrimination alone, rank correlation statistics between predictions and observations can be used, although this seldom occurs in practice. For calibration, results can be visualised in a scatterplot and an observed versus predicted line fitted. For a perfectly calibrated model, this line is on the diagonal; for an overfit (underfit) model, the calibration line is above (below) the diagonal. A smooth calibration line can assess calibration locally—ie, it can indicate areas where the model underestimates or overestimates the outcome. Smooth calibration lines can be obtained by fitting, for example, restricted cubic splines or a locally estimated scatterplot smoothing line (LOESS) of the predicted versus the observed outcomes.

**Binary outcomes**

Discrimination can be assessed using the area under the receiver operating characteristic curve (AUC). Mean calibration (calibration in the large, see table 1) can be determined by comparing mean observed versus mean predicted event rates. A logistic regression model can be fit to the observed outcome using the log odds of the event from the prediction model as the sole independent variable and then the intercept and slope can be evaluated. Additionally, a calibration curve can be created; for this, participants are grouped according to their predicted probabilities. Calculate the mean predicted probability and the proportion of events for each group; then compare the two in a scatterplot and draw a smooth calibration curve (eg, using splines) to assess calibration locally. The Brier score measures overall performance—it is simply calculated as the mean squared difference between predicted probabilities and actual outcomes. Many additional measures can be used to measure performance, for example, F score, sensitivity-specificity, etc.

**Survival outcomes**

If focus is on a specific time point, discrimination can be assessed as for binary outcomes (fixed time point discrimination).^18^ However, censoring of follow-up times complicates this assessment. Uno and colleagues' inverse probability of censoring weights method can account for censoring.^82^ Also, discrimination can be assessed across all time points using Harrell's c statistic.^83^ Uno's c statistic can be expanded to a global measure, across all time points.^84^ Calibration can be assessed for a fixed time point by comparing the average predicted survival from the model with the observed survival—ie, estimated while accounting for censorship; this can be obtained from a Kaplan-Meier curve by looking at the specific time point (calibration in the large at a fixed time). The Kaplan-Meier curve can be compared with the mean predicted survival across all times. More details can be found elsewhere.^18^ Smooth calibration curves can also be used to assess performance of the model across the full range of predicted risks, while additional calibration metrics have also been proposed.^85^ ^86^ Similar measures can be used for competing events, with some adjustments.^16^

### Model validation

What data should we use to assess the performance of a prediction model? The simplest approach is to use the same dataset as for model development; this approach will return the so-called apparent model performance (apparent validation). However, this strategy might overestimate the model’s performance (fig 1); that is, it might lead to erroneous (optimistic) assessments. Optimism is an important issue in prediction modelling and is particularly relevant when sample sizes are small and models complex. Therefore, assessing model performance using a more adequate validation procedure is crucial. Proper validation is essential in determining a prediction model’s generalisability—that is, its reproducibility and transportability.^33^ ^47^ Reproducibility refers to the model’s ability to produce accurate predictions in new patients from the same population. Transportability is the ability to produce accurate predictions in new patients drawn from a different but related population. Below, we describe different approaches to model validation.

### Internal validation

Internal validation focuses on reproducibility and specifically aims to ensure that assessments of model performance using the development dataset are honest, meaning optimism does not influence them. In an internal validation procedure, we use data on the same patient population as the one used to develop the model and try to assess model performance while avoiding optimism. Validation must follow all steps of model development, including variable selection.

The simplest method is the split sample approach where the dataset is randomly split into two parts (eg, 70% training and 30% testing). However, this method is problematic because it wastes data and decreases statistical power.^55^ ^87^ When applied to a small dataset, it might create two datasets that are inadequate for both model development and evaluation. Conversely, for large datasets it offers little benefit because the risk of overfitting is low. Further, it might encourage researchers to repeat the procedure until they obtain satisfactory results.^88^ Another approach is to split the data according to the calendar time of patient enrolment. For example, we might develop the model using data from an earlier period and test it in patients enrolled later. This procedure (temporal validation)^35^ ^89^ might inform us about possible time trends in model performance. However, the time point used for splitting the data will generally be arbitrary and older data might not reflect current patient characteristics or health care. Therefore, this approach is not recommended for the development phase.^88^

A better method is k-fold cross validation. In this approach, we divide the data randomly in k (usually 10) subsets (folds). The model is built using k−1 of these folds and evaluated on the remaining one fold. This process is repeated, cycling through all the folds so that each can be the testing set. The model's performance is measured in each cycle, and the k estimates are then combined and summarised to get a final performance measure. Bootstrapping is another method,^90^ which can be used to calculate optimism and optimism corrected performance measures for any model. Box 2 outlines the procedure.^47^ Bootstrapping generally leads to more stable and less biased results,^93^ and is therefore recommended for internal validation.^47^ However, implementation of k-fold cross validation and bootstrapping can be computationally demanding when multiple imputation of missing data is needed.^88^

Box 2 **Calculating optimism corrected measures of performance through bootstrapping**

Use bootstrapping to correct apparent performance and obtain optimism corrected measures for any model M and any performance measure as follows.

Select a measure X (eg, R^2^, mean squared error, AUC (area under the receiver operating characteristic curve)) and calculate apparent performance (X_0_) of model M in the original sample.

1. Create many (at least N_B_=100) bootstrap samples with the same size as the original dataset by drawing patients from the study population with replacement. Replacement means that some individuals might be included several times in a bootstrap sample, while others might not appear at all.
2. In each bootstrap sample i (i=1, 2 … N_B_) construct model M_i_ by exactly reiterating all steps of developing M, ie, including variable selection methods (if any were used). Determine the apparent performance X_i_ of model M_i_ in sample i.
3. Apply M_i_ to the original sample and calculate performance, X_i_*. This performance will generally be worse than X_i_ owing to optimism. Calculate optimism for measure X, sample i, as O_i_^X^=X_i_−X_i_*.
4. Average the N_B_ different values of O_i_^X^ to estimate optimism, O^X^.
5. Calculate the optimism corrected value of X as X_corrected_=X_0_−O_i_^X^.

More advanced versions of bootstrapping (eg, the 0.632+ bootstrap^91^) require slightly different procedures.^92^ In practice, we often need to combine bootstrapping with multiple imputation. Ideally, we should first bootstrap and then impute.^92^ However, this strategy might be computationally difficult. Instead, we can first impute, then bootstrap, obtain optimism corrected performance measures from each imputed dataset, and finally pool these.

Another method of assessing whether a model’s predictions are likely to be reliable or not is by checking the model’s stability. Model instability means that small changes in the development dataset lead to large changes in the resulting model structure (important differences in estimates of model parameters, included predictors, etc), leading to important changes in predictions and model performance. Riley and Collins described how to assess the stability of clinical prediction models during the model development phase using a bootstrap approach.^94^ The model building procedure is repeated in several bootstrap samples to create numerous models. Predictions from these models are then compared with the original model predictions to investigate possible instability.

### Internal-external validation

An alternative approach is the internal-external or leave-one-out cross validation. This method involves partitioning the data into clusters based on a specific variable (eg, different studies, hospitals, general practices, countries) and then iteratively using one cluster as the test set while training the model on the remaining clusters.^95^ ^96^ Like in k-fold cross validation, this process is repeated for each cluster, and the performance results are summarised at the end. In contrast to k-fold cross validation, internal-external validation can provide valuable insights into how well the model generalises to new settings and populations because it accounts for heterogeneity across different clusters. For example, prediction models for patients with HIV were developed based on data from treatment programmes in Côte d’Ivoire, South Africa, and Malawi and validated using leave-one-country-out cross validation.^97^

Note here that although all internal and internal-external validation methods include some form of data splitting, the final model should be developed using data from all patients. This strategy contrasts with the external validation method outlined below.

### Εxternal validation

External validation requires testing the model on a new set of patients—that is, those not used for model development.^36^ Assuming that the model has shown good internal validity, external validation studies are the next step in determining a model’s transportability before considering its implementation in clinical practice. The more numerous and diverse the settings in which the model is externally validated, the more likely it will generalise to a new setting. An external validation study could indicate that a model requires updating before being used in a new setting. A common scenario is when a model’s discrimination is adequate in new settings and fairly stable over time, but calibration is suboptimal across settings or deteriorates over time (calibration drift).^98^ For example, EuroSCORE is a model developed in 1999 for predicting mortality in hospital for patients undergoing cardiac surgery.^99^ Using data from 2001 to 2011, EuroSCORE was shown to consistently overestimate mortality and its calibration deteriorated over time.^100^ In such situations, model updating (step 2) might be required.

The inclusion of external validation in model development is a topic of debate, with certain journals mandating it for publication.^88^ ^100^ One successful external validation, however, does not establish transportability to many other settings, while such a requirement might lead to the selective reporting of validation data.^100^ Therefore, our view (echoing recent recommendations^88^) is that external validation studies should be separated from model development at the moment of model development. External validation studies are ideally performed by independent investigators who were not involved in the original model development.^101^ For guidance on methods for external validation, see references cited in step 2.

## Step 10: Decide on the final model

Now it is time to choose the final model based on the internal and internal-external validation performance metrics (and possibly on stability assessments). If different modelling strategies perform similarly, we might want to select the simpler model (related to Occam’s razor principle^102^). For example, logistic regression performed similarly to optimised machine learning models for discriminating between type 1 and type 2 diabetes in young adults.^103^ In this case, we would prefer the regression model because it is simpler and easier to communicate and use.

## Step 11: Perform a decision curve analysis

A prediction model might strongly discriminate and be well calibrated, but its value depends on how we intend to use it in clinical practice. While an accurate prediction model can be valuable in counselling patients on likely outcomes, determining its utility in guiding decisions is less straightforward. Decision analysis methods can be used to assess whether a prediction model should be used in practice by incorporating and quantifying its clinical impact, considering the anticipated benefits, risks, and costs.^104^ For example, the National Institute for Health and Care Excellence (NICE) in the UK recommends cholesterol lowering treatment if the predicted 10 year risk of myocardial infarction or stroke is 10% or higher (the cut-off threshold probability) based on the QRISK3 risk calculator.^60^ ^105^ The assumption is that the benefit of treating one patient who would experience a cardiovascular event over 10 years outweighs the harms and costs incurred by treating another nine people who will not benefit. In other words, the harm associated with not treating the one patient who would develop the event is assumed to be nine times greater than the consequences of treating a patient who does not need it.

Net benefit brings the benefits and harms of a decision strategy (eg, to decide for or against treatment based on a prediction model) on the same scale so they can be compared.^104^ We can compute the net benefit of using the model at a particular cut-off threshold (eg, 10% risk for the case of QRISK3 risk calculator). The net benefit is calculated as the expected percentage of true positives minus the expected percentage of true negatives, multiplied by a weight determined by the chosen cut-off threshold. We obtain the decision curve by plotting the model's net benefit across a range of cut-off thresholds deemed clinically relevant.^106^ ^107^ We can compare the benefit of making decisions based on the model with alternative strategies, such as treating everyone or no one. We can also compare different models. The choice of decision threshold can be subjective, and the range of sensible thresholds will depend on the settings, conditions, available diagnostic tests or treatments, and patient preferences. The lower the threshold, the more unnecessary tests or interventions we are willing to accept. Of note, a decision curve analysis might indicate that a model is not useful in practice despite its excellent predictive ability.

There are several pitfalls in the interpretation of decision curves.^24^ Most importantly, the decision curve cannot determine at what threshold probability the model should be used. Moreover, because the model’s predictive performance influences the decision curve, the decision curve can be affected by optimism. Therefore, a model’s good predictive performance (in internal validation and after correction for optimism) should be established before evaluating its clinical usefulness through a decision curve. Additionally, the curve can be obtained using a cross validation approach.^108^ Vickers and colleagues provide a helpful step-by-step guide to interpreting decision curve analysis, and a website with a software tutorial and other resources.^107^ The multiple sclerosis example below includes a decision curve analysis.

## Step 12: Assess the predictive ability of individual predictors (optional step)

In prediction modelling, the primary focus is typically not on evaluating the importance of individual predictors; rather, the goal is to optimise the model’s overall predictive performance. Nevertheless, identifying influential predictors might be of interest, for example, when evaluating the potential inclusion of a new biomarker as a routine measurement. Also, some predictors might be modifiable, raising the possibility that they could play a part in prevention if their association with the outcome is causal. Therefore, as an additional, optional step, researchers might want to assess the predictive capacity of the included predictors.

Looking at estimated coefficients in (generalised) linear regression models is a simple way to assess the importance of different predictors. However, when the assumptions of linear regression are not met, for example, when there is collinearity, these estimates might be unreliable. However, note that multicollinearity does not threaten a model's predictive performance, just at the interpretation of the coefficients. Another method to assess the importance of a predictor, also applicable to machine learning models, is to fit the model with and without this predictor and note the reduction in model performance; omitting more important predictors will lead to a larger reduction in performance. More advanced methods include the permutation importance algorithm^109^ and SHAP (Shapley additive explanations)^110^; we do not discuss these here.

Regardless of the method we choose to assess predictor importance, we should be careful in our interpretations; associations seen in data might not reflect causal relationships (eg, see the “Table 2 fallacy”^111^). A thorough causal inference analysis is needed to establish causal associations between predictors and outcomes.^112^

## Step 13: Write up and publish

Congratulations to us! We have developed a clinical prediction model! Now, it is time to write the paper and describe the process and results in detail. The TRIPOD reporting guideline and checklist^10^ ^14^ (or, for clustered datasets, TRIPOD cluster^13^) should be used to ensure all important aspects are covered in the paper. If possible, the article should report the full model equation to allow reproducibility and independent external validation studies. Software code and, ideally, data should be made freely available. Further, we must ensure the model is accessible to the users we defined in step 1. Although this should be self-evident, in practice, there is often no way to use published models to make an actual prediction; for example, Reeve and colleagues found that 52% of published models for multiple sclerosis could not be used in practice because no model coefficients, tools, or instructions were provided.^45^

The advantages and disadvantages of different approaches for making the model available to users, including score systems, graphical score charts, nomograms, and websites and smartphone applications have been reviewed elsewhere.^113^ Simpler approaches are easier to use, for example, on ward rounds, but might require model simplification by removing some predictors or categorising continuous variables. Online calculators where users input predictor values (eg, a web application using Shiny in R)^114^ can be based on the whole model without information loss. However, if publicly accessible, calculators might be misused by people for whom they are not intended, or if the model fails to show any clinical value (eg, in a subsequent external validation). Generally, the presentation and implementation should always be discussed with the users to match their needs (defined in step 1).

## Example: relapsing-remitting multiple sclerosis

### Background

Multiple sclerosis is a chronic inflammatory disorder of the central nervous system with a highly variable clinical course.^115^ Relapsing-remitting multiple sclerosis (RRMS), the most common form, is characterised by attacks of worsening neurological function (relapses) followed by periods of partial or complete recovery (remissions).^116^ ^117^ ^118^ These fluctuations pose a major challenge in managing the disease. A predictive tool could inform treatment decisions. Below, we describe the development of a prediction model for RMMS.^119^ We briefly outline the procedures followed in the context of our step-by-step guide. Details of the original analysis and results are provided elsewhere.^119^

### Step-by-step model development

The aim was to predict relapse within two years in patients with RRMS. Such a prediction can help treatment decisions; if the risk of relapsing is high, patients might consider intensifying treatment, for example, by taking more active disease modifying drugs, which might however have a higher risk of serious adverse events, or considering stem cell transplantation. A multidisciplinary team comprising clinicians, patients, epidemiologists, and statisticians was formed. A literature review identified several potential predictors for relapse in RRMS. Additionally, the review showed limitations of existing prediction models, including lack of internal validation, inadequate handling of missing data, and lack of assessment of clinical utility (step 1). These deficiencies compromised the reliability and applicability of existing models in clinical settings. Based on the review, it was decided to pursue the development of a new model, instead of updating an existing one (step 2). The authors chose the (binary) occurrence of at least one relapse within a two year period for people with RRMS (step 3) as the outcome measure.

The following predictors were used based on the literature review and expert opinion: age, expanded disability status scale score, previous treatment for multiple sclerosis, months since last relapse, sex, disease duration, number of previous relapses, and number of gadolinium enhanced lesions. The selection aimed to include relevant predictors while excluding those that are difficult to measure in clinical practice (step 4). The model was developed using data from the Swiss Multiple Sclerosis Cohort,^120^ a prospective cohort study that closely monitors patients with RRMS. Data included a total of 1752 observations from 935 patients followed up every two years, with 302 events observed (step 5). Sample size calculations^50^ indicated a minimum sample of 2082 patients, which is larger than the available sample, raising concerns about possible overfitting issues (step 6). Multiple imputations were used to impute missing covariate data. The authors expected no missing data when using the model in practice (step 7).

**Fig 2:**
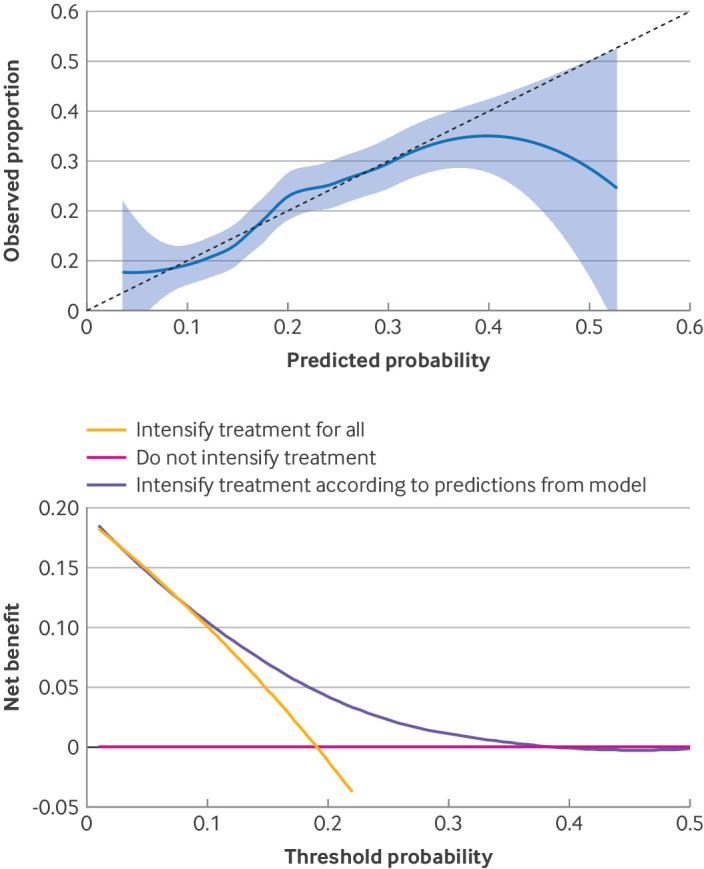
Results from a model predicting the probability of a patient with relapsing-remitting multiple sclerosis experiencing a relapse in the next two years. Figures adapted from Chalkou et al.^119^ Upper panel: calibration plot. Solid blue line shows calibration using a LOESS (locally estimated scatterplot smoothing line), and shaded area shows 95% confidence intervals. Dotted blue line corresponds to perfect calibration. Maximum predicted probability was around 60% for this example. The model is well calibrated for predicted probabilities lower than 35%. Lower panel: decision curve analysis comparing net benefit of three strategies deciding on whether to intensify treatment in patients with relapsing-remitting multiple sclerosis (from no treatment to first line treatment, or from first line to second line treatment, etc). The strategies are to continue current treatment (do not intensify), to intensify treatment for all, or to intensify treatment according to predictions from model considering probability of experiencing a relapse in next two years—ie, if predicted probability is higher than a threshold (shown on x axis), then the treatment can be intensified

A Bayesian logistic mixed effects prediction model was developed, which accounted for several observations within patients. Regression coefficients were penalised through a Laplace prior distribution to address possible overfitting (step 8). Model calibration was examined in a calibration plot (fig 2, upper panel), and discrimination was assessed using the AUC (area under the receiver operating characteristic curve). Both assessments were corrected for optimism through a bootstrap validation procedure (described in box 2), with 500 bootstrap samples created for each imputed dataset. The optimism corrected calibration slope was 0.91, and the optimism corrected AUC was 0.65—this value corresponds to low to moderate discriminatory ability, comparable to or exceeding previous RRMS models (steps 9 and 10). A decision curve analysis was performed to assess the clinical utility of the model (fig 2, lower panel). The analysis indicated that deciding to intensify or not intensify the treatment using information from the model is preferable to simpler strategies—do not intensify treatment, and intensify treatment for all—for thresholds between 15% and 30%. Therefore, the model is useful to guide decisions in practice only if we value the avoidance of relapse 3.3–6.6 times more than the risks and inconveniences of more intensive treatments (step 11). Among the included predictors, younger age, higher expanded disability status scale scores, and shorter durations since the last relapse were associated with higher odds of experiencing a relapse in the next two years according to the estimated regression coefficients. However, none of the predictors were modifiable factors (step 13). The model was implemented in a freely available R-shiny^114^ web application, where patients, doctors, and decision makers can estimate the probability of experiencing at least one relapse within the next two years (https://cinema.ispm.unibe.ch/shinies/rrms/ ). To enable reproducibility, all code was made publicly available at https://github.com/htx-r/Reproduce-results-from-papers/tree/master/PrognosticModelRRMS (step 13).

## Software

Our appendix is available online at https://github.com/esm-ispm-unibe-ch/R-guide-to-prediction-modelling, where we provide R code covering many aspects of the development of prediction models. The code uses simulated datasets and describes the case of continuous, binary, time-to-event, and competing risk outcomes. The code covers the following aspects: sample size calculations, multiple imputation, modelling nonlinear associations, assessing apparent model performance, performing internal validation using bootstrap, internal-external validation, and decision curve analysis. Readers should note that the appendix does not cover all possible modelling methods, models, and performance measures that can be used. Moreover, parts of the code are based on previous publications.^16^ ^18^ Additional code is provided elsewhere, for example, by Zhou and colleagues.^17^

## Conclusions

**Fig 3:**
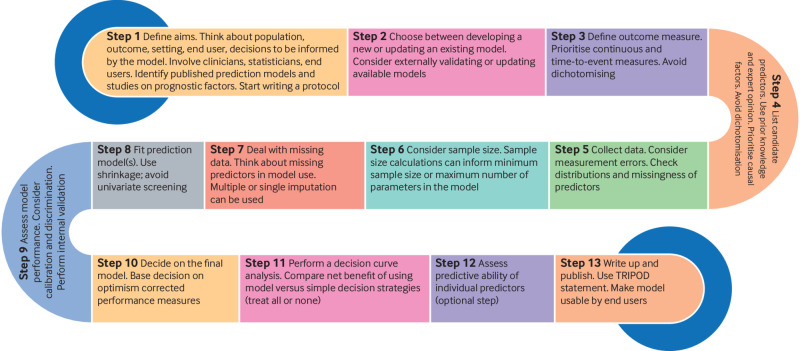
Graphical overview of 13 proposed steps for developing a clinical prediction model. TRIPOD=transparent reporting of a multivariable prediction model for individual prognosis or diagnosis

This tutorial provides a step-by-step guide to developing and validating clinical prediction models. We stress that this is not a complete and exhaustive guide, and it does not aim to replace existing resources. Our intention is to introduce essential aspects of clinical prediction modelling. Figure 3 provides an overview of the proposed steps.

In principle, most steps we have described apply to traditional statistical and machine learning approaches,^14^ with some exceptions. For example, the structure of a machine learning model is often defined during model development and so will not be known a priori. Consequently, using the final model for multiple imputations, as we discussed in step 7, might not be possible. Further, bootstrapping, which we recommended as the method of choice for internal validation, might not be computationally feasible for some machine learning approaches. Moreover, some machine learning approaches might require additional development steps to ensure calibration.^94^ ^121^ ^122^

We trust that our presentation of the key concepts and discussion of topics relevant to the development of clinical prediction models will help researchers to choose the most sensible approach for the problem at hand. Moreover, the paper will hopefully increase awareness among researchers of the need to work in diverse teams, including clinical experts, methodologists, and future model users. Similar to guidance on transparent reporting of research, adopting methodological guidance to improve the quality and relevance of clinical research is a responsibility shared by investigators, reviewers, journals, and funders.^123^

## Web extra

Extra material supplied by authors

Web appendix 1: Appendix

## Notes

Contributors: OE conceived the idea of the project and wrote the first draft of the manuscript. KC performed the analysis of the real example in relapsing-remitting multiple sclerosis. MS and OE prepared the online supplement. ME and GS contributed concepts and revised the manuscript. All authors contributed to the final manuscript. OE is the guarantor of the article. ME and GS contributed equally to the manuscript as last authors. The corresponding author attests that all listed authors meet authorship criteria and that no others meeting the criteria have been omitted.

Provenance and peer review: Not commissioned; externally peer reviewed.
