# Supplementary material for: Developing clinical prediction models: a step-by-step guide
Source: BMJ. 2024 Sep 3;386:e078276. doi: 10.1136/bmj-2023-078276 (PMC11369751; doi:10.1136/bmj-2023-078276)
Supplement: Supplementary file 1 — Web appendix 1: Appendix [file efto078276.ww.pdf]

Appendix to the paper:

Developing clinical prediction models: a step-by-step practical guide with examples in R

Contents

1 Sample size calculations ... 3

2 Example for continuous outcomes ... 3

2.1 Simulate a toy example ... 3

2.2 Perform multiple imputations ... 5

2.3 Fit the competing prediction models ... 5

2.4 Calculate apparent performance of the models ... 12

2.5 Calculate optimism-corrected performance of the models ... 12

2.6 Perform internal-external cross-validation ... 14

3 Example for binary outcomes ... 18

3.1 Simulate a toy example ... 18

3.2 Perform multiple imputations ... 19

3.3 Fit the competing prediction models ... 19

3.4 Calculate apparent performance of the models ... 23

3.5 Calculate optimism-corrected performance of the models ... 25

3.6 Perform internal-external cross-validation ... 26

3.7 Decision curve analysis ... 29

4 Example for time-to-event outcomes ... 31

4.1 Simulate a toy example ... 31

4.2 Visualize data ... 32

4.3 Perform multiple imputations ... 33

4.4 Fit a prediction model ... 33

4.5 Calculate apparent performance of the model ... 35

4.6 Calculate optimism-corrected performance of the models ... 39

4.7 Perform internal-external cross-validation ... 40

4.8 Decision curve analysis ... 41

5 Example for competing risks outcomes ... 43

5.1 Simulate a toy example ... 43

5.2 Perform multiple imputations 44
5.3 Visualize the data 44
5.4 Fit a prediction model in the imputed datasets 45
5.5 Calculate apparent performance of the model 47
5.6 Calculate optimism-corrected performance of the model 51
5.7 Perform internal-external cross-validation 52
5.8 Obtain uncertainty intervals for performance measures 54
5.9 Decision curve analysis 56
6 References 57

1 Sample size calculations

Sample size calculations can be done using the **`pmsampsize`** package in R[1]. Here is an example for a continuous outcome.

```
library(pmsampsize)
pmsampsize(type = "c", rsquared = 0.6, parameters = 10, intercept = 0, sd =
1)
```

User needs to define the expected R^2, the number of parameters of the models, the expected intercept and standard deviation of the outcome. Additional parameters can be set by hand – see the help file of the package. For a binary outcome we need to provide instead an expected c-statistic of the model, and prevalence of the outcome:

```
pmsampsize(type = "b", cstatistic = 0.80, parameters = 12,
           prevalence = 0.12)
```

For a time-to-event outcome we can instead use e.g.

```
pmsampsize(type = "s", rsquared = 0.10, parameters = 15, rate = 0.12,
           timepoint = 1, meanfup = 1.25)
```

As mentioned in our main paper, it is often the case that sample size is fixed. In this case we can use this code to back-calculate the maximum number of parameters (i.e. degrees of freedom) that can be included in the model. Then, we can spend these degrees of freedom to pre-select which predictors, non-linear, or interaction terms to include in the model, given the existing sample size.

2 Example for continuous outcomes

In this section, we use a simulated example to illustrate some of the steps discussed in the main document, particularly with respect to model development, for the case of a continuous outcome.

2.1 Simulate a toy example

The dataset includes:

- five predictors `x1, x2, x3, x4, x5`, of which `x1, x2` are continuous, `x3, x4` binary, and `x5` categorical.
- the outcome `y` we want to predict.
- five auxiliary variables `z1, z2, z3, z4` and `z5`.
- a clustering variable `clust`.

The aim is to build a model using the predictors (`x`) to predict the outcome (`y`). The auxiliary variables (`z`) will only be used for imputation of missing variables and outcomes. Auxiliary variables may be variables that are related to predictors or the outcome, but ones that will not be used for the prediction model. For example, an intermediate outcome cannot be used for predicting y (because its value is not known at time zero, i.e. at baseline), but may still be useful in imputing missing values. The `clust` variable provides information about an important stratification of the patients in the data. For example, it may be that the data was collected in different centres. This variable will not be used in the model per se but may be useful for imputation and for the internal-external cross-validation process.

```
rm(list=ls()) # empty memory
set.seed(42) # the answer to life the universe and everything
library(MASS)
N <- 100
Sigma <- outer(1:10, 1:10, function(x,y) 0.5^abs(x-y)) #variance covariance
matrix for covariates
```

```
x <- mvrnorm(N, rep(0,10), Sigma)
x[,3]<- ifelse(x[,3] > 0.5, 1, 0)
x[,4] <- ifelse(x[,4] > 0, 1, 0)
x[,5] <- cut(x[,5], breaks=c(-Inf, -1, 0, 1, Inf), labels = FALSE)
x[,8] <- ifelse(x[,8] > -0.5, 1, 0)
x[,9] <- ifelse(x[,9] > 0.5, 1, 0)
x[,10] <- cut(x[,10], breaks=c(-Inf, -1, 0, 1, Inf), labels = FALSE)

data.cont.complete <- data.frame(x)
colnames(data.cont.complete) <- c(paste0("x", 1:5), paste0("z", 1:5))

data.cont.complete$y<-with(data.cont.complete, x1+0.2*x1^2+0.5*x2-
0.2*x2^2+0.3*x3+0.2*x4+
                             0.2*(x5==2)-
0.1*(x5==3)+0.4*(x5==4)+rnorm(N,0,1))
data.cont.complete[,c(3:5, 8:10)] <- lapply(data.cont.complete[,c(3:5,
8:10)], factor)
head(data.cont.complete)
```

Above we have used the **`MASS`** package.[2] We will now create some missing data and add a clustering variable.

```
missing.matrix=matrix(0, nrow=nrow(data.cont.complete),
ncol=ncol(data.cont.complete))
missing.matrix=matrix(rbinom(length(missing.matrix),1, p=0.1),
nrow=nrow(data.cont.complete))
data.cont=data.cont.complete
data.cont[missing.matrix==1]=NA
```

This is what the data looks like:

```
head(data.cont)
            x1          x2 x3   x4   x5         z1         z2 z3   z4 z5          y clust
4   1.05863018  1.87572000  1    0    2 -1.7989668 -1.9059333  0    0  3  2.3939380     1
21 -0.88456382  0.76148109  0 <NA> <NA>  1.3763961  0.9986441  1    1  4  1.1785683     1
23 -0.16254908 -0.20319098  0    0    3         NA  0.8798468  0 <NA>  3  0.9866370     1
25 -1.55744244 -0.61766584  0 <NA>    1         NA -1.3144854  0    0  4 -1.9483904     1
26  0.07578796 -0.07518767  0    0    2  1.5922787  0.3666538  1    0  2  0.6266513     1
30  1.27672866          NA  0    1    3 -0.6970741 -0.3550338  1    1  3  0.9573074     1
```

This is the distribution of the observed outcomes, using **`ggplot2`**:[3]

```
library(ggplot2)
ggplot(data.cont, aes(x=y)) + geom_histogram(color="black", fill="white",
binwidth = 0.5)
```

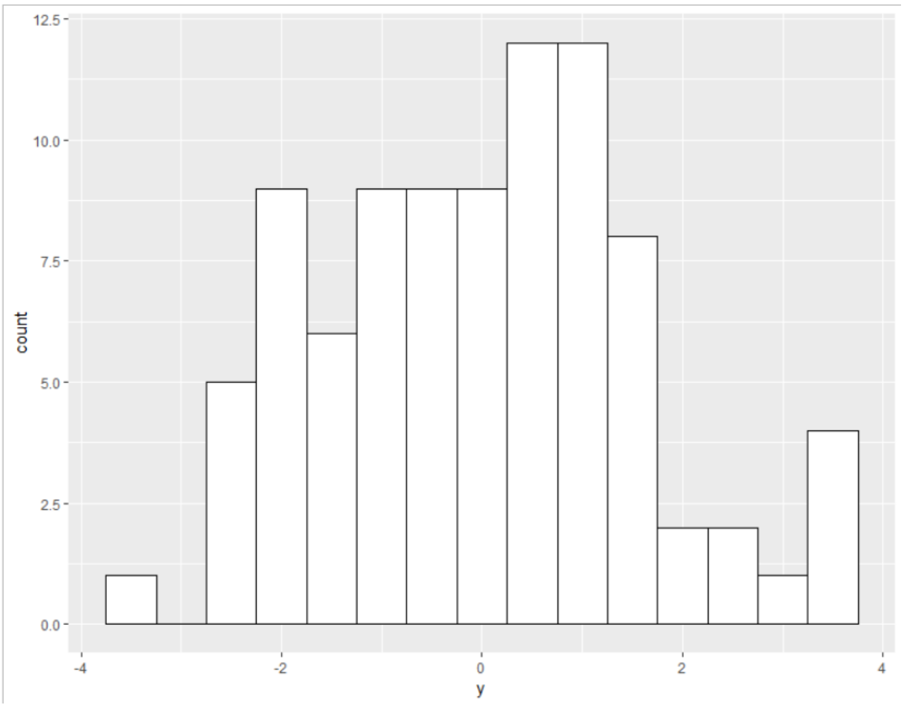

2.2 Perform multiple imputations

We will use the `areg.impute` function from the **`Hmisc`** package.[4] Since our prediction models will include splines for `x1` and `x2`, we need to include splines in the imputation model.

```
library(Hmisc)
n.impute <- 10
a <- aregImpute(data=data.cont, I(y)~x1+x2+I(x3)+I(x4)+I(x5)+
                z1+z2+I(z3)+I(z4)+I(z5)+clust, n.impute=n.impute, nk=3,
match='closest')

# get imputed datasets
imputed1 <- list()
for (i in 1:n.impute){
  imputed1[[i]] <- impute.transcan(a, imputation=i, data=data.cont,
list.out=TRUE, pr=FALSE, check=FALSE)}
```

2.3 Fit the competing prediction models

Now that we have created the imputed datasets, we can fit our models. Let us start with a ordinary least squares regression model with splines for the two continuous predictors, `x1` and `x2`. Note that since the imputation method used auxiliary variables to impute the outcome, it makes sense to use patients with imputed outcomes when building the model. We use the `ols` function from **`rms`**.[5]

```
library(rms)
regression.splines<-list()
for (i in 1:n.impute){regression.splines[[i]]<-
ols(y~rcs(x1,3)+rcs(x2,3)+x3+x4+x5,data=imputed1[[i]])}
```

Let us plot[6] the fitted splines, to get a sense about their shape:

```
library(gridExtra)
p.sp1<-ggplot(data.frame(x1=seq(-3,3, 0.1),
              y=Predict(regression.splines[[i]], x1=seq(-3,3, 0.1), x2=0,
x3=1, x4=1, x5=2)$yhat), aes(x=x1, y=y)) + geom_smooth()
p.sp2<-ggplot(data.frame(x2=seq(-3,3, 0.1),
              y=Predict(regression.splines[[i]], x1=1, x2=seq(-3,3, 0.1),
x3=1, x4=1, x5=2)$yhat), aes(x=x2, y=y)) + geom_smooth()
```

```
grid.arrange(p.sp1, p.sp2, ncol=2)
```

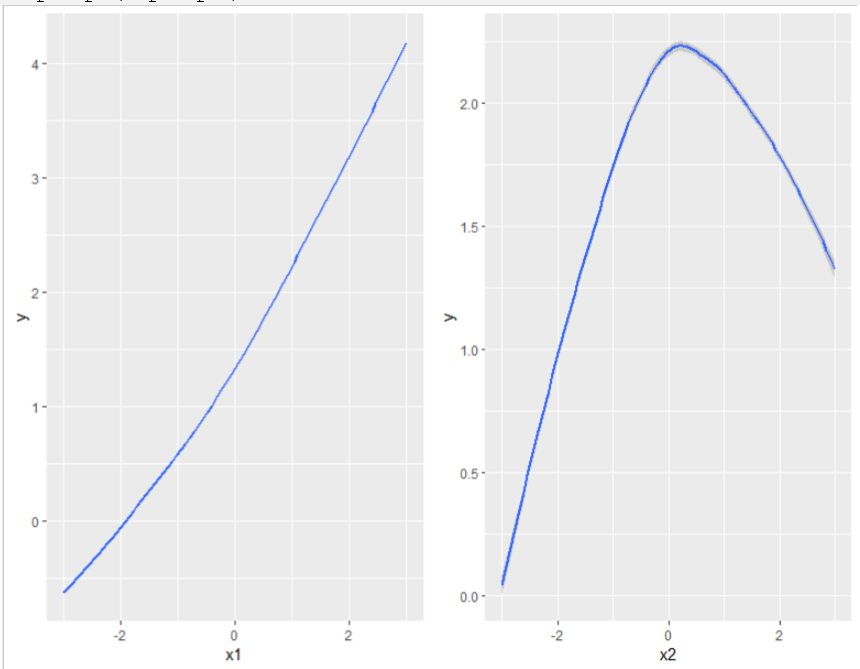

We have fit a separate prediction model in each of the imputed dataset. To obtain a single prediction for a new patient, we can either:

i. Use Rubin's rules to pool the coefficients of the prediction models; or
ii. Use the models to make separate predictions and average these predictions in the end.

Approach ii. is easier to use, particularly with complicated models with many predictors (e.g. machine learning methods), or when the model has different structure in each imputed dataset (e.g. when it includes variable selection). In what follows we will use this second method. We will define a function for making predictions for a dataframe of new patients.

```
prediction.ols <- function(new.patient, single.fit = NULL, multiple.fit =
NULL){

  if(!is.null(multiple.fit)){
    mygrid <- expand.grid(k = 1:dim(new.patient)[1], i =
1:length(multiple.fit))

    ff <- function(k,i){
      with(new.patient, Predict(multiple.fit[[i]], x1= x1[k], x2 = x2[k],
x3= x3[k], x4= x4[k], x5= x5[k])$y)
    }

    prediction_matrix <- matrix(mapply(ff, mygrid$k, mygrid$i),
 nrow = dim(new.patient)[1], ncol = length(multiple.fit))
    prediction <- apply(prediction_matrix, 1, mean)
  } else if(!is.null(single.fit)){
    ff <- function(k){
      with(new.patient, Predict(single.fit, x1= x1[k], x2 = x2[k], x3=
x3[k], x4= x4[k], x5= x5[k])$y)
    }
    prediction <- sapply(1:dim(new.patient)[1], ff)
  }
   return(prediction)
}
```

Let us now use the model to predict for a new patient.

```
new.patient<-data.frame(x1=1.2, x2=-1.6, x3=0, x4=1, x5=4)
[1] 0.6871744
```

Let us also use the model to make predictions for patients in the dataset and compare with observations. We will only use patients with complete observations on the five predictors and the outcome.

```
complete.data <- data.cont[complete.cases(data.cont[,c(1:5,11)]),]
predicted.ols <- prediction.ols(complete.data, multiple.fit =
regression.splines) # these are patients with fully observed data
ggplot(data.frame(observed=complete.data$y, predicted.ols=predicted.ols),
        aes(x=predicted.ols, y=observed)) +
        geom_point(size=1) +
        geom_abline(intercept = 0, slope = 1,
color="black",linetype="dashed", size=0.5) +
        xlim(-3,3) + ylim(-3,3)+geom_smooth(method=lm)
```

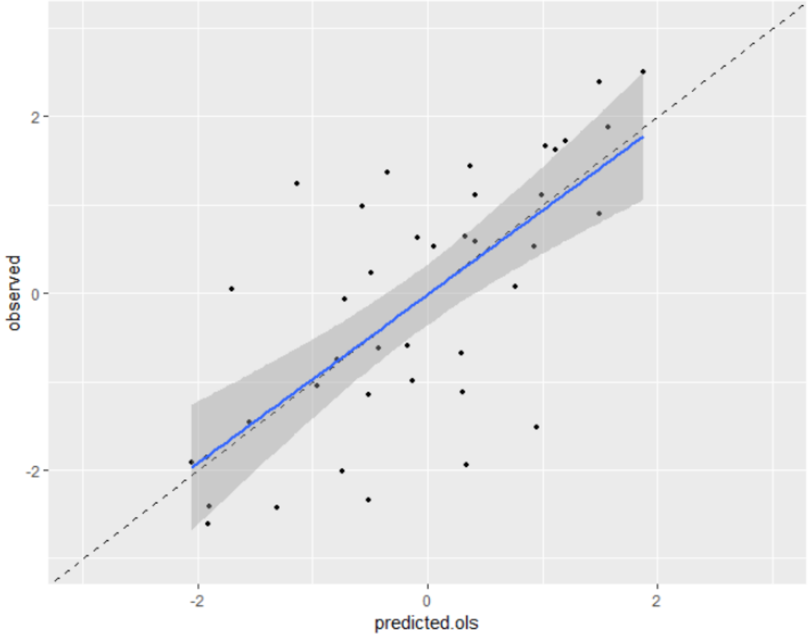

Here we see that the model is well calibrated on average. Let us now fit a generalized additive model (GAM). This model allows for a non-linear relationship between the continuous predictor and the outcome, while utilizing penalization (shrinkage). We will use the **mgcv** package.[7]

```
library(mgcv)
fit.gam=list()
for(i in 1:n.impute){fit.gam[[i]] <- gam(y ~ x3 + x4 + x5 + s(x1) + s(x2),
data = imputed1[[i]])}
```

We can now use the fitted models to make predictions

```
prediction.gam <- function(new.patient, single.fit = NULL, multiple.fit =
NULL){
  if(!is.null(multiple.fit)){
    mygrid <- expand.grid(k = 1:dim(new.patient)[1],i =
1:length(multiple.fit))

    ff <- function(k,i){
      predict.gam(multiple.fit[[i]], newdata = new.patient[k,])}

    prediction_matrix <- matrix(mapply(ff, mygrid$k, mygrid$i),
                                nrow = dim(new.patient)[1], ncol =
length(multiple.fit))
    prediction <- apply(prediction_matrix, 1, mean)
  } else if(!is.null(single.fit)){
```

```
    ff <- function(k){
      predict.gam(single.fit, newdata = new.patient[k,])
    }
    prediction <- sapply(1:dim(new.patient)[1], ff)   }

  return(prediction)     }

prediction.gam(new.patient, multiple.fit = fit.gam)
```

```
[1] 0.8556169
```

We can visualize the GAMs

```
new1 <- data.frame(x1=seq(-3,3, 0.2), x2=0, x3=1, x4=1, x5=2)
p.g1 <- ggplot(data.frame(x1=seq(-3,3, 0.2),
              y=prediction.gam(new1, multiple.fit = fit.gam)), aes(x=x1,
y=y)) +
              geom_smooth()

new2 <- data.frame(x1=2, x2=seq(-3,3, 0.2), x3=1, x4=1, x5=2)
p.g2 <- ggplot(data.frame(x2=seq(-3,3, 0.2),
              y=prediction.gam(new2, multiple.fit = fit.gam)), aes(x=x2,
y=y)) +
              geom_smooth()
grid.arrange(p.g1, p.g2, ncol=2)

ggplot(data.frame(observed=complete.data$y,
      predicted.gam=predicted.gam),
      aes(x=predicted.gam, y=observed)) + geom_point(size=1) +
      geom_abline(intercept = 0, slope = 1, color="black",
linetype="dashed", size=0.5) +
      xlim(-5,5) + ylim(-5,5)
```

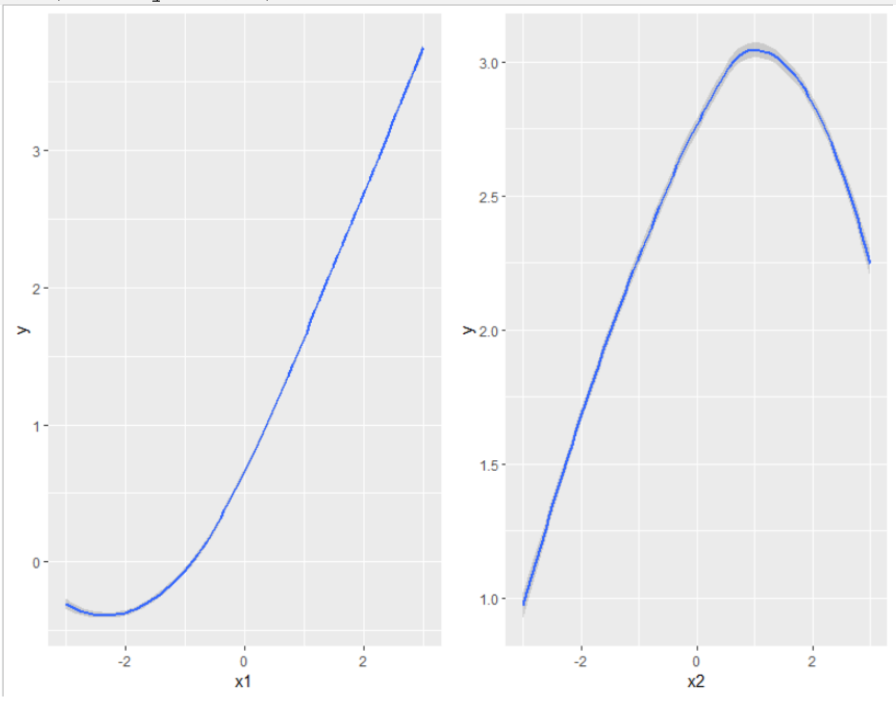

We can make predictions for patients in the dataset with complete predictors and compare with observed outcomes

```
predicted.gam <- prediction.gam(complete.data, multiple.fit = fit.gam)
ggplot(data.frame(observed=complete.data$y,
      predicted.gam=predicted.gam),
      aes(x=predicted.gam, y=observed)) + geom_point(size=1) +
```

```
      geom_abline(intercept = 0, slope = 1, color="black",
linetype="dashed", size=0.5) +
      xlim(-3,3) + ylim(-3,3)+geom_smooth(method=lm)
```

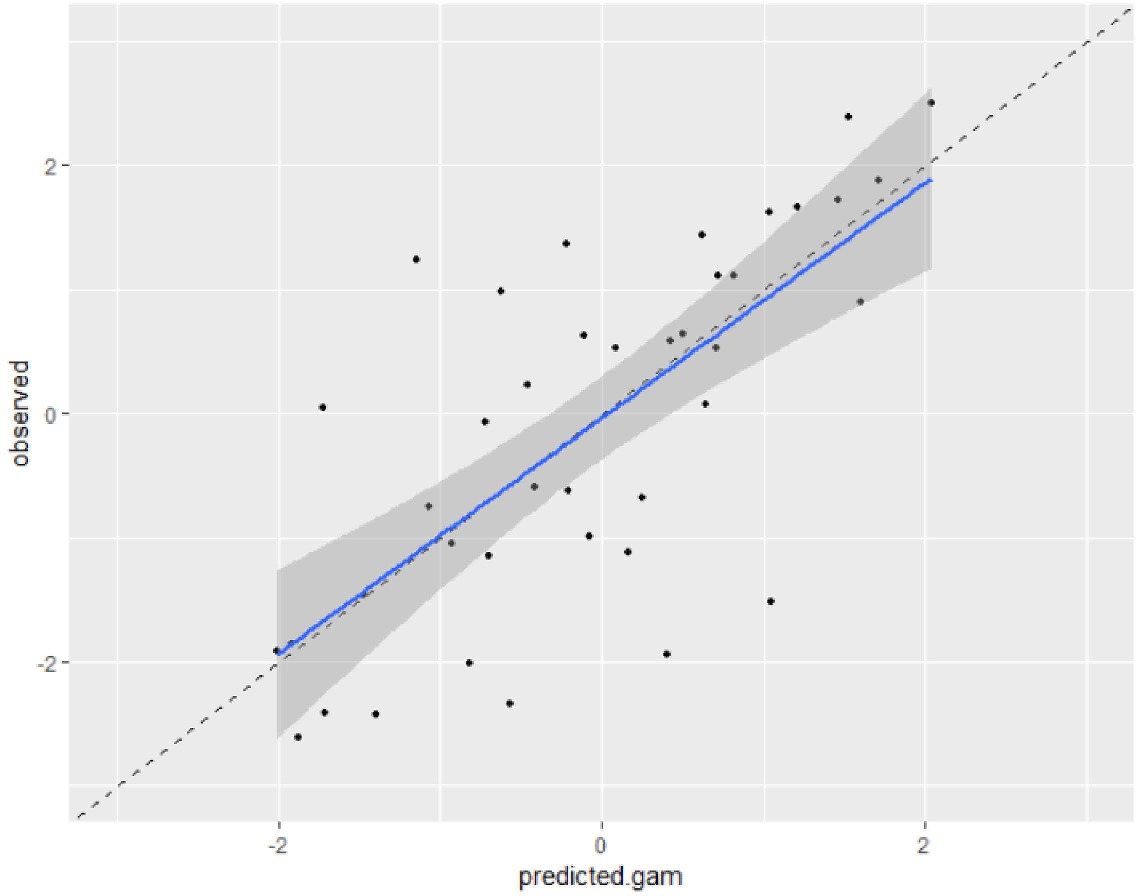

Let us also fit a third model, a ridge regression model using the packages **`glmnet`** and **`splines2`**.[8,9]

```
library(splines2)
library(glmnet)

lambdas <- 10^seq(2, -10, by = -0.3)
#first find the position of the knots for the splines
bsMat.x1 <- bSpline(data.cont$x1[complete.cases(data.cont$x1)],
                    knots = quantile(data.cont$x1, c(0.25, 0.5, 0.75),
na.rm = TRUE))
bsMat.x2 <- bSpline(data.cont$x2[complete.cases(data.cont$x2)],
                    knots = quantile(data.cont$x2, c(0.25, 0.5, 0.75),
na.rm = TRUE))

fit.ridge <- list()
for( i in 1:n.impute){
  dfSplined.x1 <- as.data.frame(predict(bsMat.x1, imputed1[[i]]$x1))
  dfSplined.x2 <- as.data.frame(predict(bsMat.x2, imputed1[[i]]$x2))
  imp <- imputed1[[i]]
  imp <- cbind(imp$y, imp$x3, imp$x4, imp$x5, dfSplined.x1, dfSplined.x2)
  colnames(imp) <- c("y", "x3", "x4", "x5", paste0("V",
as.character(1:(length(colnames(imp))-4))))
  data_glmnet <- model.matrix(y ~., data = imp)
  X <- as.matrix(data_glmnet[,-1])
  colnames(X)[1:2] <- c("x3", "x4")
  Y <- imp$y
```

```
  cvfit <- cv.glmnet(X,Y,family = "gaussian", alpha=0,
                     lambda = lambdas, nfolds=10)
  lambda.min <- cvfit$lambda.min
  fit.ridge[[i]] <- glmnet(X,Y,family = "gaussian", alpha=0, lambda =
lambda.min)
}
```

Now use the fitted models to make predictions

```
prediction.ridge <- function(new.patient, single.fit = NULL, multiple.fit =
NULL){

  if(!is.null(multiple.fit)){
    mygrid <- expand.grid(k = 1:dim(new.patient)[1],i =
1:length(multiple.fit))

    ff <- function(k,i){

      dfSplined.x1 <- as.data.frame(predict(bsMat.x1, new.patient$x1[k]))
      dfSplined.x2 <- as.data.frame(predict(bsMat.x2, new.patient$x2[k]))

      imp <-
data.frame(x3=1*(new.patient$x3[k]==1),x4=1*(new.patient$x4[k]==1),x52=1*(n
ew.patient$x5[k]==2),
                        x53=1*(new.patient$x5[k]==3),
x54=1*(new.patient$x5[k]==4), dfSplined.x1,
                        dfSplined.x2)
      colnames(imp)=c("x3","x4","x52","x53","x54", paste0("V",
as.character(1:(length(colnames(imp))-5))))
      predict(multiple.fit[[i]], newx = as.matrix(imp))
    }
    prediction_matrix <- matrix(mapply(ff, mygrid$k, mygrid$i),
                                nrow = dim(new.patient)[1], ncol =
length(multiple.fit))
    prediction <- apply(prediction_matrix, 1, mean)
  } else if(!is.null(single.fit)){

    ff <- function(k){

      dfSplined.x1 <- as.data.frame(predict(bsMat.x1, new.patient$x1[k]))
      dfSplined.x2 <- as.data.frame(predict(bsMat.x2, new.patient$x2[k]))

      imp <-
data.frame(x3=1*(new.patient$x3[k]==1),x4=1*(new.patient$x4[k]==1),x52=1*(n
ew.patient$x5[k]==2),
                        x53=1*(new.patient$x5[k]==3),
x54=1*(new.patient$x5[k]==4), dfSplined.x1,
                        dfSplined.x2)
      colnames(imp)=c("x3","x4","x52","x53","x54", paste0("V",
as.character(1:(length(colnames(imp))-5))))

      predict(single.fit, newx = as.matrix(imp))
    }
    prediction <- sapply(1:dim(new.patient)[1], ff)
  }
  return(prediction)
}

prediction.ridge(new.patient, multiple.fit = fit.ridge)
```

```
[1] 0.759369
```

```
predicted.ridge <- prediction.ridge(complete.data, multiple.fit =
fit.ridge)

ggplot(data.frame(observed=complete.data$y,
                  predicted.ridge=predicted.ridge),
       aes(x=predicted.ridge, y=observed))+ geom_point(size=1)+
       geom_abline(intercept = 0, slope = 1, color="black",
       linetype="dashed", size=0.5) +
       xlim(-3,3) + ylim(-3,3)+geom_smooth(method=lm)
```

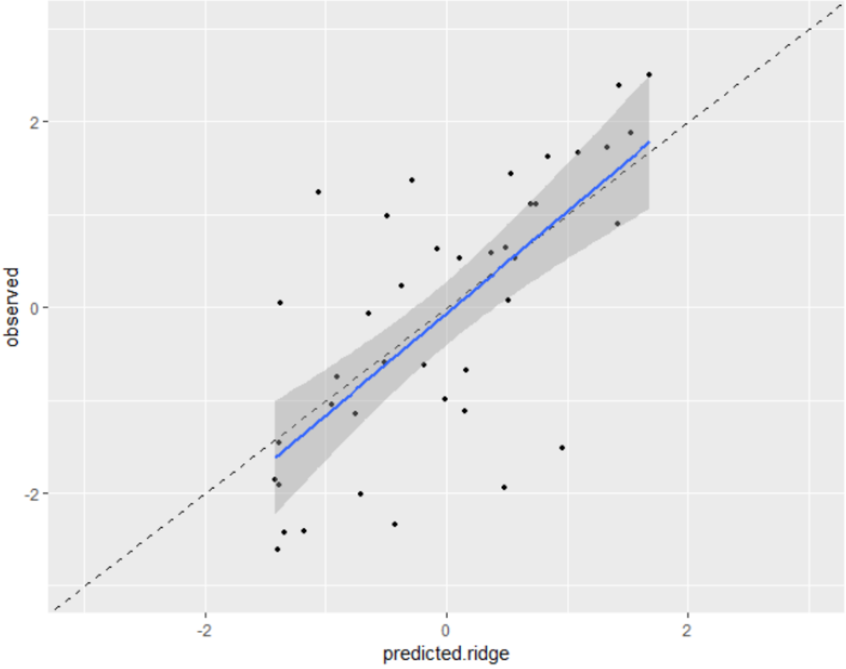

We can now compare the predictions from the three models

```
p1<-ggplot(data.frame(predicted.gam=predicted.gam,
predicted.ols=predicted.ols),
        aes(x=predicted.ols, y=predicted.gam))+  geom_point(size=1)+
        geom_abline(intercept = 0, slope = 1,
color="black",linetype="dashed", size=0.5)+
        xlim(-4.5,4.5)+ylim(-4.5,4.5)

p2<-ggplot(data.frame(predicted.ols=predicted.ols,
predicted.ridge=predicted.ridge),
        aes(x=predicted.ridge, y=predicted.ols))+  geom_point(size=1)+
        geom_abline(intercept = 0, slope = 1,
color="black",linetype="dashed", size=0.5)+
        xlim(-4.5,4.5)+ylim(-4.5,4.5)

p3<-ggplot(data.frame(predicted.gam=predicted.gam,
predicted.ridge=predicted.ridge),
        aes(x=predicted.ridge, y=predicted.gam))+  geom_point(size=1)+
        geom_abline(intercept = 0, slope = 1,
color="black",linetype="dashed", size=0.5)+
        xlim(-4.5,4.5)+ylim(-4.5,4.5)

grid.arrange(p1, p2, p3, ncol=3)
```

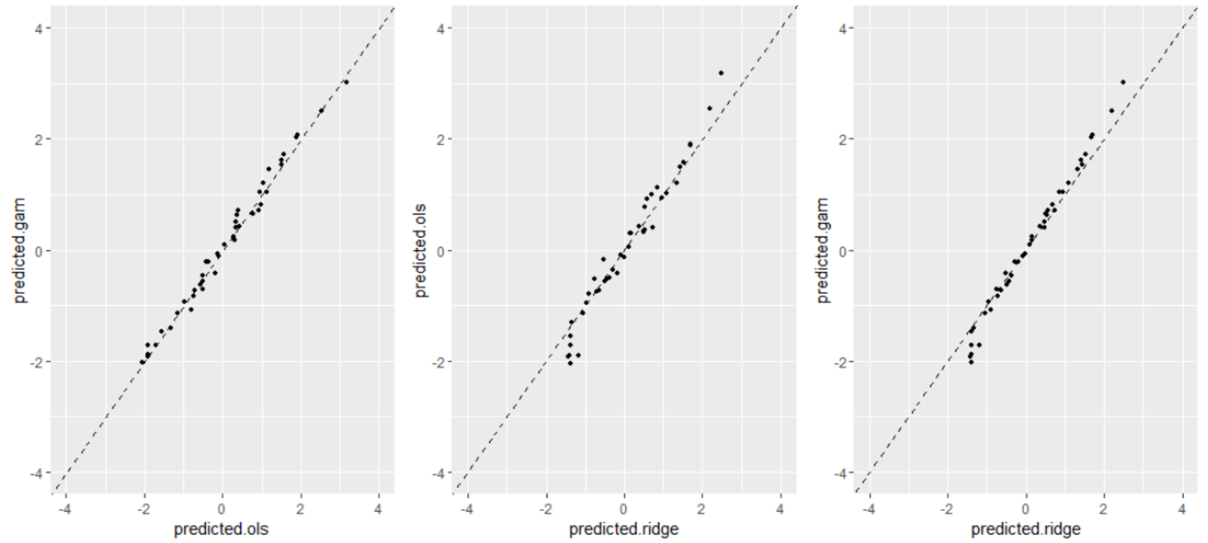

We see similar predictions for the unpenalized splines and the GAM approach, and some small differences with the predictions from ridge.

2.4 Calculate apparent performance of the models

After having obtained the predictions from each model and for each patient in the data with fully observed predictors and outcomes, it is easy to assess the models' performance, by comparing predicted vs. observed outcomes:

```
 calculate_performance <- function(observed, predicted){

  MAE <- mean(abs(observed - predicted)) # mean absolute error
  MSE <- mean((observed - predicted)^2) # mean squared error
  R2 <- summary(lm(observed~predicted))$r.squared

  vec <- c(MAE, MSE, R2)
  names(vec) <- c("MAE", "MSE", "R2")
  return(vec)
}

apparent.ols <- calculate_performance(complete.data$y, predicted.ols)
apparent.gam <- calculate_performance(complete.data$y, predicted.gam)
apparent.ridge <- calculate_performance(complete.data$y, predicted.ridge)
round(rbind(apparent.ols, apparent.gam, apparent.ridge), digits=2)
                 MAE  MSE   R2
apparent.ols    0.82 1.08 0.61
apparent.gam    0.78 1.03 0.63
apparent.ridge 0.86 1.14 0.61
```

As we discuss in the paper, however, this apparent performance may be optimistic.

2.5 Calculate optimism-corrected performance of the models

We will use bootstrapping to obtain optimism-corrected assessments of model performance, for our three models. Ideally, we should first bootstrap and then perform the multiple imputations within each bootstrap sample. This, however, would be computationally demanding. Instead, we can bootstrap the already imputed datasets. For each one of the `n.impute=10` imputed datasets we create `N.bootstrap=10` bootstrap samples. We then fit the models in the bootstrap sample and estimate bootstrap performance. We use the bootstrap models to the imputed dataset and estimate test performance. The difference between the two is optimism. We repeat for all 100

(=`n.impute * N.bootstrap`) bootstrap samples. We average, to obtain a final estimate of model optimism. We only focus on mean absolute error (MAE), mean squared error (MSE), and coefficient of determination (R^2).

```
N.bootstrap=10
optimism.ols.eachbootstrap <- optimism.gam.eachbootstrap <-
optimism.ridge.eachbootstrap <- matrix(NA, N.bootstrap, 3)
optimism.ols <- optimism.gam <- optimism.ridge <- matrix(NA, n.impute, 3)

for (i in 1:n.impute){
  for(j in 1:N.bootstrap){

    boot.sample <- sample(length(imputed1[[i]]$y),replace = T)

    # create bootstrap sample
    imp.boot <- lapply(imputed1[[i]], function(x){x[boot.sample]})

    regression.splines.boot <- ols(y~rcs(x1,3)+rcs(x2,3)+x3+x4+x5,data=
imp.boot)

    fit.gam.boot <- gam(y ~ x3 + x4 + x5 + s(x1) + s(x2), data = imp.boot)

    dfSplined.x1 <- as.data.frame(predict(bsMat.x1, imp.boot$x1))
    dfSplined.x2 <- as.data.frame(predict(bsMat.x2, imp.boot$x2))
    imp <- cbind(imp.boot$y, imp.boot$x3, imp.boot$x4, imp.boot$x5,
dfSplined.x1, dfSplined.x2)
    colnames(imp)=c("y", "x3", "x4", "x5", paste0("V",
as.character(1:(length(colnames(imp))-4))))
    data_glmnet <- model.matrix(y ~ ., data = imp)
    X <- as.matrix(data_glmnet[,-1])
    colnames(X)[1:2] <- c("x3", "x4")
    Y <- imp$y
    cvfit <- cv.glmnet(X,Y,family = "gaussian", alpha=0,
                       lambda = lambdas, nfolds=10)
    lambda.min <- cvfit$lambda.min
    fit.ridge.boot <- glmnet(X,Y,family = "gaussian", alpha=0, lambda =
lambda.min)

    # predict in bootstrap
    f1 <- as.data.frame(do.call(cbind, lapply(imp.boot, function(x)
{as.numeric(as.character(x))})))

    boot.prediction.ols <- prediction.ols(f1, single.fit =
regression.splines.boot)
    boot.prediction.gam <- prediction.gam(f1, single.fit = fit.gam.boot)
    boot.prediction.ridge <- prediction.ridge(f1, single.fit =
fit.ridge.boot)

    ols.boot <- calculate_performance(f1$y, boot.prediction.ols)
    gam.boot <- calculate_performance(f1$y, boot.prediction.gam)
    ridge.boot <- calculate_performance(f1$y, boot.prediction.ridge)

    # predict in test data
    f2 <- as.data.frame(do.call(cbind, lapply(imputed1[[i]], function(x)
{as.numeric(as.character(x))})))
    test.prediction.ols <- prediction.ols(f2, single.fit =
regression.splines.boot)
    test.prediction.gam <- prediction.gam(f2, single.fit = fit.gam.boot)
    test.prediction.ridge <- prediction.ridge(f2, single.fit =
fit.ridge.boot)
```

```
    ols.test <- calculate_performance(f2$y, test.prediction.ols)
    gam.test <- calculate_performance(f2$y, test.prediction.gam)
    ridge.test <- calculate_performance(f2$y, test.prediction.ridge)

    optimism.ols.eachbootstrap[j,] <- ols.boot - ols.test
    optimism.gam.eachbootstrap[j,] <- gam.boot - gam.test
    optimism.ridge.eachbootstrap[j,] <- ridge.boot - ridge.test  }
  optimism.ols[i,] <- apply(optimism.ols.eachbootstrap, 2, mean)
  optimism.gam[i,] <- apply(optimism.gam.eachbootstrap, 2, mean)
  optimism.ridge[i,] <- apply(optimism.ridge.eachbootstrap, 2, mean)

  print(paste0("imputation done: ", i))}

mean.optimism.ols <- apply(optimism.ols, 2, mean)
mean.optimism.gam <- apply(optimism.gam, 2, mean)
mean.optimism.ridge <- apply(optimism.ridge, 2, mean)

optimism.corrected.ols <- apparent.ols - mean.optimism.ols
optimism.corrected.gam <- apparent.gam - mean.optimism.gam
optimism.corrected.ridge <- apparent.ridge - mean.optimism.ridge
round(rbind(optimism.corrected.ols, optimism.corrected.gam,
optimism.corrected.ridge), digits=2)
```

Optimism-corrected measures of performance:

```
                          MAE  MSE   R2
optimism.corrected.ols   0.92 1.33 0.54
optimism.corrected.gam   0.98 1.67 0.44
optimism.corrected.ridge 1.02 3.85 0.45
```

Least squares regression seems to outperform the other two methods, ridge performed worse.

2.6 Perform internal-external cross-validation

Finally, we perform an internal-external cross-validation of the models, using the clustering variable, `clust`. In a nutshell, we take one cluster out, use the remaining clusters to fit the models, and make predictions for the left-out cluster. We then cycle through all clusters. At the end of the procedure, for each patient with complete data for all predictors, we have a single prediction. This prediction is obtained from models that were fit in patients in all other clusters apart from the one the patient belonged to.

```
clusters <- unique(data.cont$clust)
N.clust <- length(clusters) # 5 clusters in this example
data.in <- data.leftout <- list()

#create the datasets
for(i in 1:N.clust){
  data.in[[i]]<-data.cont[data.cont$clust!=clusters[i],]
  data.leftout[[i]]<-data.cont[data.cont$clust==clusters[i],]
  complete.index <- complete.cases(data.leftout[[i]][,c(paste0("x", 1:5),
"y")])
  data.leftout[[i]] <- data.leftout[[i]][complete.index,]
}

#impute the data and fit the model
n.impute <- 10

imputed <- regression.splines.CV <- fit.gam.CV <- fit.ridge.CV <- list()
leftout.prediction.ols <- leftout.prediction.gam <-
leftout.prediction.ridge <- list()
```

```
leftout.performance.ols <- leftout.performance.gam <-
leftout.performance.ridge <- list()

for (i in 1:N.clust){
  a <- aregImpute(data=data.in[[i]], I(y)~x1+x2+I(x3)+I(x4)+I(x5),
n.impute=n.impute, nk=3, match='closest')

  for (j in 1:n.impute){
    imputed[[j]] <- impute.transcan(a, imputation=j, data=data.in[[i]],
list.out=TRUE,
                                  pr=FALSE, check=FALSE)
    regression.splines.CV[[j]]<-
ols(y~rcs(x1,3)+rcs(x2,3)+x3+x4+x5,data=imputed[[j]])
    fit.gam.CV[[j]] <- gam(y ~ x3+x4+x5+s(x1)+s(x2), data = imputed[[j]])

    dfSplined.x1 <- as.data.frame(predict(bsMat.x1, imputed[[j]]$x1))
    dfSplined.x2 <- as.data.frame(predict(bsMat.x2, imputed[[j]]$x2))
    imp <- cbind(imputed[[j]]$y, imputed[[j]]$x3, imputed[[j]]$x4,
imputed[[j]]$x5, dfSplined.x1, dfSplined.x2)
    colnames(imp) <- c("y", "x3", "x4", "x5", paste0("V",
as.character(1:(length(colnames(imp))-4))))
    data_glmnet <- model.matrix(y ~ ., data = imp)
    X <- as.matrix(data_glmnet[,-1])
    colnames(X)[1:2] <- c("x3", "x4")
    Y <- imp$y
    cvfit <- cv.glmnet(X,Y,family = "gaussian", alpha=0,
                       lambda = lambdas, nfolds=10)
    lambda.min <- cvfit$lambda.min
    fit.ridge.CV[[j]] <- glmnet(X,Y,family = "gaussian", alpha=0, lambda =
lambda.min)
  }

  leftout.prediction.ols[[i]] <- prediction.ols(data.leftout[[i]],
multiple.fit = regression.splines.CV)
  leftout.prediction.gam[[i]] <- prediction.gam(data.leftout[[i]],
multiple.fit = fit.gam.CV)
  leftout.prediction.ridge[[i]] <- prediction.ridge(data.leftout[[i]],
multiple.fit = fit.ridge.CV)

  leftout.performance.ols[[i]] <-
calculate_performance(data.leftout[[i]]$y, leftout.prediction.ols[[i]])
  leftout.performance.gam[[i]] <-
calculate_performance(data.leftout[[i]]$y, leftout.prediction.gam[[i]])
  leftout.performance.ridge[[i]] <-
calculate_performance(data.leftout[[i]]$y, leftout.prediction.ridge[[i]])
}
# performance per cluster
#leftout.performance.ols
#leftout.performance.gam
#leftout.performance.ridge
# performance aggregating all clusters
leftout.prediction.ols <- do.call(c, leftout.prediction.ols)
leftout.prediction.gam <- do.call(c, leftout.prediction.gam)
leftout.prediction.ridge <- do.call(c, leftout.prediction.ridge)
IECV.observed <- do.call(rbind, data.leftout)$y
IECV.cluster <- do.call(rbind, data.leftout)$clust

IECV.ols <- calculate_performance(IECV.observed, leftout.prediction.ols)
IECV.gam <- calculate_performance(IECV.observed, leftout.prediction.gam)
IECV.ridge <- calculate_performance(IECV.observed,
leftout.prediction.ridge)
```

```
round(rbind(IECV.ols, IECV.gam, IECV.ridge), digits=2)
```

	MAE	MSE	R2
**IECV.ols**	0.92	1.43	0.50
**IECV.gam**	0.90	1.43	0.49
**IECV.ridge**	0.96	1.49	0.46

Let us now plot[10] results of the internal-external CV.

```
p4<-ggplot(IECV,
       aes(x=leftout.prediction.ols, y=IECV.observed, color=cluster))+
geom_point(size=2)+
      geom_abline(intercept = 0, slope = 1,
color="black",linetype="dashed", size=0.5)+
      xlim(-4,4)+ylim(-4,4) + theme(legend.position = "none")+
  geom_smooth(method=lm, se=F) +ylab("observed")

p5<-ggplot(IECV,
       aes(x=leftout.prediction.gam, y=IECV.observed, color=cluster))+
geom_point(size=2)+
  geom_abline(intercept = 0, slope = 1, color="black",linetype="dashed",
size=0.5)+
  xlim(-4,4)+ylim(-4,4) + theme(legend.position = "none")+
  geom_smooth(method=lm, se=F) +ylab("observed")

p6<-ggplot(IECV,
       aes(x=leftout.prediction.ridge, y=IECV.observed, color=cluster))+
geom_point(size=2)+
        geom_abline(intercept = 0, slope = 1,
color="black",linetype="dashed", size=0.5)+
  xlim(-4,4)+ylim(-4,4)+geom_smooth(method=lm, se=F) +ylab("observed")

library(ggpubr)
ggarrange(p4, p5, p6, ncol=3, common.legend = TRUE, legend="bottom")
```

Each point represents a patient, colours denote clusters.

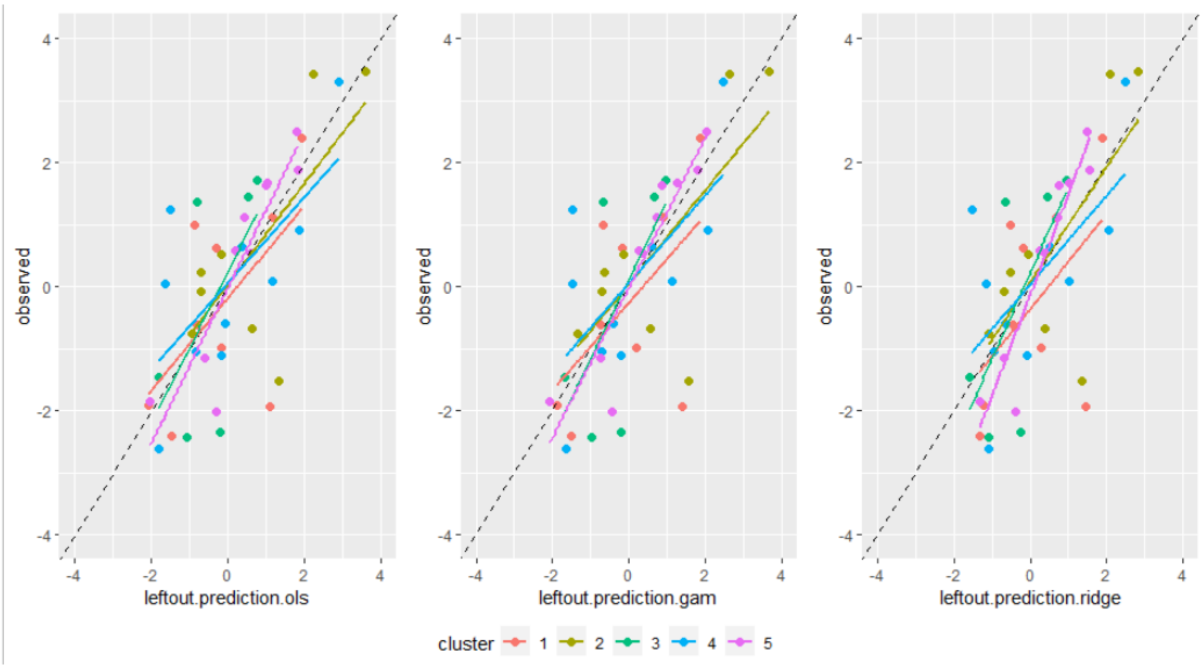

We see good calibration overall. Finally, we can look into the cluster-specific performance for a model, e.g. aiming to identify patterns across clusters

```
per.cluster.ols=matrix(leftout.performance.ols[[1]], nrow=1)
for(i in 2: N.clust){
```

```
  per.cluster.ols=rbind(per.cluster.ols,
matrix(leftout.performance.ols[[i]], nrow=1))}
per.cluster.ols=data.frame(per.cluster.ols)
colnames(per.cluster.ols)=c("MAE", "MSE", "R2")
rownames(per.cluster.ols)=paste("cluster",clusters,":")
round(per.cluster.ols, digits=2)
```

```
             MAE  MSE   R2
cluster 1 : 0.94 1.72 0.35
cluster 2 : 0.99 1.64 0.50
cluster 3 : 1.31 2.17 0.36
cluster 4 : 0.97 1.46 0.46
cluster 5 : 0.56 0.52 0.82
```

3 Example for binary outcomes

3.1 Simulate a toy example

We will follow the methods of Section 2.1 to generate an example dataset. One difference is that here we generate for each patient the log-odds of an event using a linear model of the covariates, and from that we simulate the outcome using a Bernoulli distribution.

```
remove(list=ls()) # empty memory
set.seed(42) # the answer to life the universe and everything
#useful functions
logit <- function(x){log(x/(1-x))}
expit <- function(x){exp(x)/(1+exp(x))}

## simulate data
library(MASS)
N <- 200

Sigma <- outer(1:10, 1:10, function(x,y) 0.5^abs(x-y)) #variance covariance
matrix for covariates

x <- mvrnorm(N, rep(0,10), Sigma)
x[,3] <- ifelse(x[,3] > 0.5, 1, 0)
x[,4] <- ifelse(x[,4] > 0, 1, 0)
x[,5] <- cut(x[,5], breaks=c(-Inf, -1, 0, 1, Inf), labels = FALSE)
x[,8] <- ifelse(x[,8] > 1, 0.5, 0)
x[,9] <- ifelse(x[,9] > 1.5, 1, 0)
x[,10] <- cut(x[,10], breaks=c(-Inf, -1, 0.5, 1, Inf), labels = FALSE)

data.bin.complete <- data.frame(x)
colnames(data.bin.complete) <- c(paste0("x", 1:5), paste0("z", 1:5))

logit.py <- with(data.bin.complete,-2+x1+0.2*x1^2+
0.3*x2+0.1*x2^2+0.2*(x3==2)+0.2*(x4==2)+0.2*(x5==2)-
0.1*(x5==3)+0.2*(x5==4)+rnorm(N,0,0.1))
py <- expit(logit.py)
data.bin.complete$y <- rbinom(N,1,py)
data.bin.complete[,c(3:5, 8:10, 11)] <- lapply(data.bin.complete[,c(3:5,
8:10, 11)], factor)

# introduce missing data
missing.matrix=matrix(0, nrow=nrow(data.bin.complete),
ncol=ncol(data.bin.complete))
missing.matrix=matrix(rbinom(length(missing.matrix),1, p=0.1),
nrow=nrow(data.bin.complete))
data.bin=data.bin.complete
data.bin[missing.matrix==1]=NA
```

Let us see the number of events in the dataset:

```
table(data.bin$y)
  0   1
137  42
```

Obviously the dataset is very small (N=200) and with very few events; in real applications we would probably not use such a dataset to develop a model. However, we use it here for illustration purposes. Next, we create a clustering variable:

```
#create a clustering variable
data.bin$clust <- factor(sample(1:5, size = N, replace = TRUE, prob =
rep(0.2,5)))
```

```
data.bin <- data.bin[order(data.cont$clust),]
data.bin <- data.bin[order(data.bin$clust),]
head(data.bin)
           x1         x2   x3 x4 x5         z1          z2  z3 z4 z5 y clust
11 -0.6914087 -1.2313564    0  0  1         NA  1.53889813   0  0  1 0     1
21 -1.1537552  0.8313891    1  1  1         NA -0.18854078   0  0  2 0     1
30  0.9075665         NA    1  1  3  0.3859338 -0.60386017   0  0  2 0     1
31  0.5666889  0.4127938    0  0  1 -1.8668800 -0.07871549   0  0  4 0     1
32 -0.1716803  0.0967226    0  1  3 -0.3332295          NA   0  0  2 0     1
36  0.7355847  0.5791860 <NA>  0  4  0.6506267  0.88753494 0.5  1  2 1     1
```

The true probability of an event for a new patient, according to the data generating mechanism:

```
new.patient <- new.patient <- data.frame(x1=-0.3, x2=-0.5, x3=1, x4=1, x5=2)
new.logit <- with(new.patient,-2 +0.5*x1+0.1*x1^2+0.2*x2-0.05*x2^2+0.1*(x3==2)+0.2*(x4==2)+0.2*(x5==2)-0.1*(x5==3)+0.2*(x5==4))  # this is the true log odds
expit(new.logit)
[1] 0.1124456
```

3.2 *Perform multiple imputations*

We can now perform the multiple imputations using the same code[4] as in section 2.2:

```
library(Hmisc)
n.impute <- 10
a <- aregImpute(data=data.bin,
I(y)~x1+x2+I(x3)+I(x4)+I(x5)+z1+z2+z3+z4+z5+clust
 n.impute=n.impute, nk=3, match='closest')
# get imputed datasets
imputed1=list()
for (i in 1:n.impute){
  imputed1[[i]] <- impute.transcan(a, imputation=i, data=data.bin, list.out=TRUE,pr=FALSE, check=FALSE)}
```

3.3 *Fit the competing prediction models*

Similar to the example of the continuous outcome, we provide code to fit three models. First, a logistic regression with splines:[5]

```
library(rms)
regression.splines<-list()
for (i in 1:n.impute){  regression.splines[[i]]<-lrm(y~rcs(x1,3)+rcs(x2,3)+x3+x4+x5, data=imputed1[[i]]) }
```

Let us plot the fitted splines

```
spl1<-ggplot(data.frame(x1=seq(-3,3, 0.1),
            logit.py=Predict(regression.splines[[i]], x1=seq(-3,3,0.1), x2=0, x3=1, x4=1, x5=2)$yhat),
            aes(x=x1, y=logit.py)) + geom_smooth()

spl2<-ggplot(data.frame(x2=seq(-3,3, 0.1),
            logit.py=Predict(regression.splines[[i]], x1=1, x2=seq(-3,3,0.1), x3=1, x4=1, x5=2)$yhat),
            aes(x=x2, y=logit.py)) + geom_smooth()

library(gridExtra)
grid.arrange(spl1,spl2,ncol=2)
```

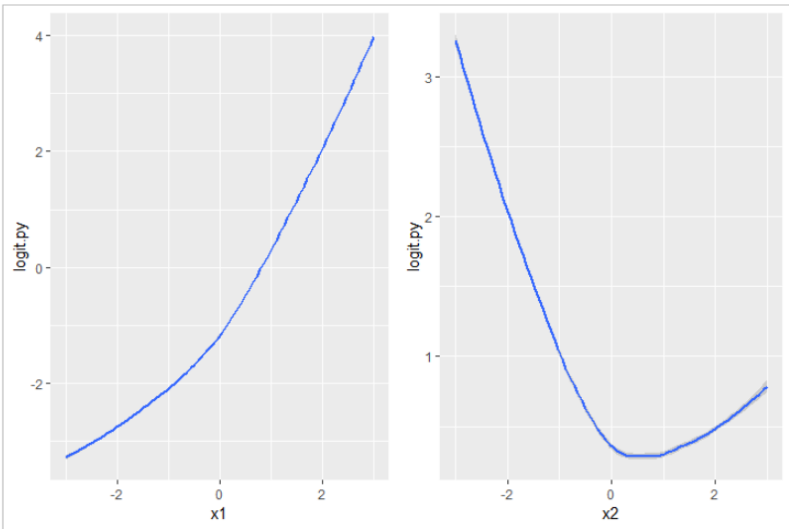

We can now use the fitted models (one per imputed dataset) to make predictions about new patients.

```
prediction.lr <- function(new.patient, single.fit = NULL, multiple.fit =
NULL){
  if(!is.null(multiple.fit)){
    mygrid <- expand.grid(k = 1:dim(new.patient)[1],i =
1:length(multiple.fit))

    ff <- function(k,i){
      with(new.patient, Predict(multiple.fit[[i]], x1= x1[k], x2 = x2[k],
x3= x3[k], x4= x4[k], x5= x5[k])$y)     }

    prediction_matrix <- matrix(mapply(ff, mygrid$k, mygrid$i),
                                nrow = dim(new.patient)[1], ncol =
length(multiple.fit))
    prediction <- apply(prediction_matrix, 1, mean)
  } else if(!is.null(single.fit)){
    ff <- function(k){
      with(new.patient, Predict(single.fit, x1= x1[k], x2 = x2[k], x3=
x3[k], x4= x4[k], x5= x5[k])$y)     }
    prediction <- sapply(1:dim(new.patient)[1], ff)   }
  return(prediction)}
```

The predicted probability of an event for the new patients created above is:

```
expit(prediction.lr(new.patient, multiple.fit = regression.splines))
[1] 0.1941315
```

i.e. quite far from the true probability given above (0.11). We can also calculate probability of an event for all patients with complete data in our dataset, and show them in a histogram:

```
complete.data <-
data.bin[complete.cases(data.bin[,c("x1","x2","x3","x4","x5","y")]),]
predicted.lr <- prediction.lr(complete.data, multiple.fit =
regression.splines) # these are patients with fully observed data
ggplot(data.frame(p=expit(predicted.lr)), aes(x=p)) +
      geom_histogram() + geom_histogram(color="black", fill="white")
```

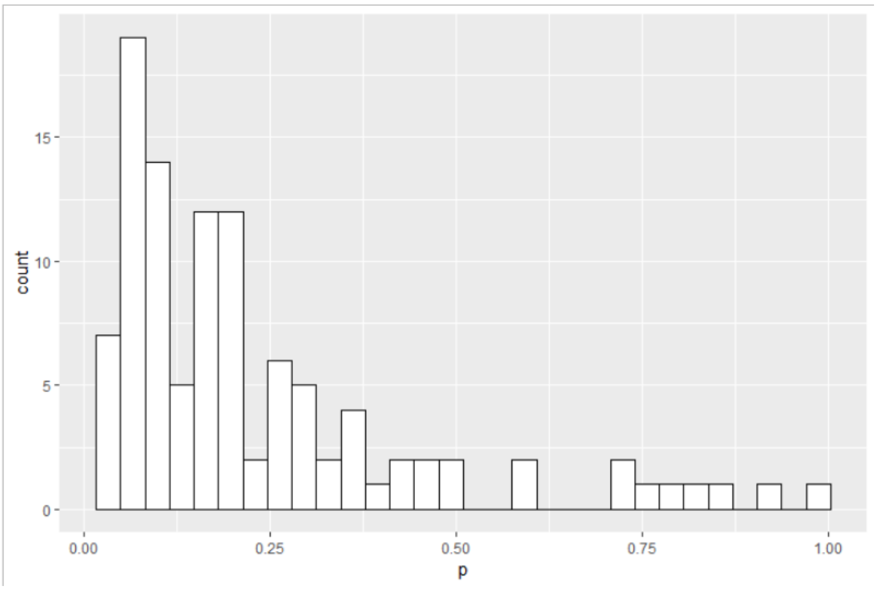

The second model is a GAM:[7]

```
library(mgcv)
fit.gam <- list()
for( i in 1:n.impute){
  fit.gam[[i]] <- gam(y~x3+x4+x5+s(x1)+s(x2), data = imputed1[[i]],
family=binomial)}

prediction.gam <- function(new.patient, single.fit = NULL, multiple.fit =
NULL){
  if(!is.null(multiple.fit)){
    mygrid <- expand.grid(k = 1:dim(new.patient)[1],i =
1:length(multiple.fit))

    ff <- function(k,i){
      predict.gam(multiple.fit[[i]], newdata = new.patient[k,])
    }
    prediction_matrix <- matrix(mapply(ff, mygrid$k, mygrid$i
nrow = dim(new.patient)[1], ncol = length(multiple.fit))
    prediction <- apply(prediction_matrix, 1, mean)
  } else if(!is.null(single.fit)){

    ff <- function(k){
      predict.gam(single.fit, newdata = new.patient[k,])
    }
    prediction <- sapply(1:dim(new.patient)[1], ff)
  }
 return(prediction)
}
```

Predict in the complete dataset:

```
predicted.gam <- prediction.gam(complete.data, multiple.fit = fit.gam)
```

Predict for a new patient:

```
expit(prediction.gam(new.patient, multiple.fit = fit.gam))
[1] 0.1928841
```

The third model is a ridge regression with only linear associations:

```
library(glmnet)
lambdas <- 10^seq(2, -10, by = -0.3)
fit.ridge <- list()
for( i in 1:n.impute){
  imp <- imputed1[[i]]
  imp <- with(imp, data.frame(y, x1, x2, x3, x4, x5))
  data_glmnet <- model.matrix(y ~.,data = imp)
```

```
  data_glmnet <- data_glmnet[,-1]
  data_glmnet <- cbind(y = as.numeric(as.character(imp$y)), data_glmnet =
data_glmnet)
  X <- as.matrix(data_glmnet[,-1])
  colnames(X)[3:4] <- c("x3", "x4")
  Y <- data_glmnet[,1]
  cvfit <- cv.glmnet(X,Y,family = "binomial",alpha=0, lambda = lambdas,
nfolds=10)
  lambda.min <- cvfit$lambda.min
  fit.ridge[[i]] <- glmnet(X,Y,family = "binomial",alpha=0, lambda =
lambda.min)
}

# predict for new patients
prediction.ridge <- function(new.patient, single.fit = NULL, multiple.fit =
NULL){
  if(!is.null(multiple.fit)){
    mygrid <- expand.grid(k = 1:dim(new.patient)[1],i =
1:length(multiple.fit))
    ff <- function(k,i){
      imp <- with(new.patient, data.frame(x1[k], x2[k], x3[k], x4[k], x52=
x5[k]==2, x53= x5[k]==3, x54 = x5[k]==4))
      imp[,3:7] <- lapply(imp[,3:7], as.numeric)
      colnames(imp) <- c(paste0("x",1:4), "x52", "x53", "x54")
      predict(multiple.fit[[i]], newx = as.matrix(imp))
    }
    prediction_matrix <- matrix(mapply(ff, mygrid$k, mygrid$i),
    nrow = dim(new.patient)[1], ncol = length(multiple.fit))
    prediction <- apply(prediction_matrix, 1, mean)
  } else if(!is.null(single.fit)){
    ff <- function(k){
          imp <- with(new.patient, data.frame(x1[k], x2[k], x3[k], x4[k],
x52= x5[k]==2, x53= x5[k]==3, x54 = x5[k]==4))
      imp[,3:7] <- lapply(imp[,3:7], as.numeric)
      colnames(imp) <- c(paste0("x",1:4), "x52", "x53", "x54")
      predict(single.fit, newx = as.matrix(imp))
    }
    prediction <- sapply(1:dim(new.patient)[1], ff)
  }
  return(prediction)
}
```

Predict in the complete dataset:

```
predicted.ridge <- prediction.ridge(complete.data, multiple.fit =
fit.ridge)
```

Predict for a new patient:

```
expit(prediction.ridge(new.patient, multiple.fit = fit.ridge))
[1] 0.2303426
```

Let us now compare the three predictions in the log-odds scale:

```
axislim1=c(-5,3)
p1<-ggplot(data.frame(predicted.gam=predicted.gam,
predicted.lr=predicted.lr),
          aes(x=predicted.lr, y=predicted.gam))+  geom_point(size=1)+
          geom_abline(intercept = 0, slope = 1,
color="black",linetype="dashed", size=0.5)+
          xlim(axislim1)+ylim(axislim1)

p2<-ggplot(data.frame(predicted.lr=predicted.lr,
predicted.ridge=predicted.ridge),
          aes(x=predicted.ridge, y=predicted.lr))+  geom_point(size=1)+
```

```
          geom_abline(intercept = 0, slope = 1,
color="black",linetype="dashed", size=0.5)+
          xlim(axislim1)+ylim(axislim1)

p3<-ggplot(data.frame(predicted.gam=predicted.gam,
predicted.ridge=predicted.ridge),
          aes(x=predicted.ridge, y=predicted.gam))+  geom_point(size=1)+
          geom_abline(intercept = 0, slope = 1,
color="black",linetype="dashed", size=0.5)+
          xlim(axislim1)+ylim(axislim1)
grid.arrange(p1, p2, p3, ncol=3)
```

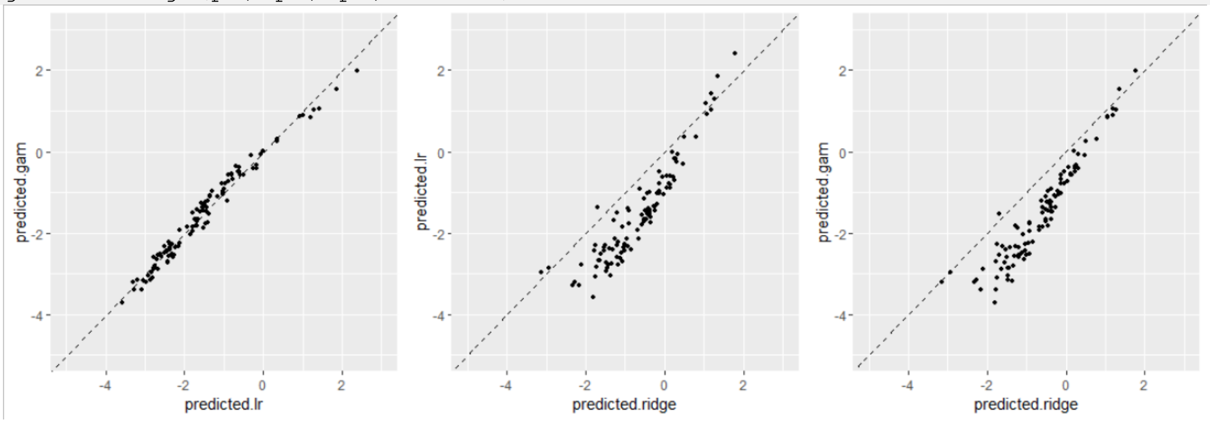

We see similar predictions for logistic regression and GAM, and important differences with the predictions from ridge (which only included linear terms).

3.4 Calculate apparent performance of the models

For binary outcomes we need to assess performance in terms of discrimination and calibration. We will use the **pROC** package.[11]

```
library(pROC)
apparent.auc.LR<-auc(complete.data$y~predicted.LR)
apparent.auc.gam<-auc(complete.data$y~predicted.gam)
apparent.auc.ridge<-auc(complete.data$y~predicted.ridge)

#calibration in the large
mean(complete.data$y=="1")
[1] 0.2095238
mean(expit(predicted.LR))
[1] 0.2311552
mean(expit(predicted.gam))
[1] 0.2329497
mean(expit(predicted.ridge))
[1] 0.3687317
```

We see that the mean estimated event rate by ridge is quite off. Next, we fit calibration lines and calculate AUC:

```
 calculate_performance2 <- function(observed, predicted){
  auc <- auc(observed~predicted)
  glm.fit <- summary(glm(observed~predicted, family = binomial))
  calibration.intercept <- glm.fit$coef[1,1]
  calibration.slope <- glm.fit$coef[2,1]
  vec <- c(auc, calibration.intercept, calibration.slope)
  names(vec) <- c("auc", "calibration intercept", "calibration slope")
  return(vec)
}

library(pROC)
apparent.lr <- calculate_performance2(complete.data$y, predicted.lr)
```

```
apparent.gam <- calculate_performance2(complete.data$y, predicted.gam)
apparent.ridge <- calculate_performance2(complete.data$y, predicted.ridge)
round(rbind(apparent.lr, apparent.gam, apparent.ridge),2)
                auc calibration intercept calibration slope
apparent.lr    0.82                  0.05              1.22
apparent.gam   0.83                  0.08              1.28
apparent.ridge 0.80                 -0.83              1.49
```

We can also assess performance using the `val.prob` function in **`rms`**:[5]

```
val.prob(y=as.numeric(complete.data$y)-1,p=expit(predicted.LR))
val.prob(y=as.numeric(complete.data$y)-1,p=expit(predicted.gam))
val.prob(y=as.numeric(complete.data$y)-1,p=expit(predicted.ridge))
```

Here is the plot for the first model, showing that it is relatively well calibrated.

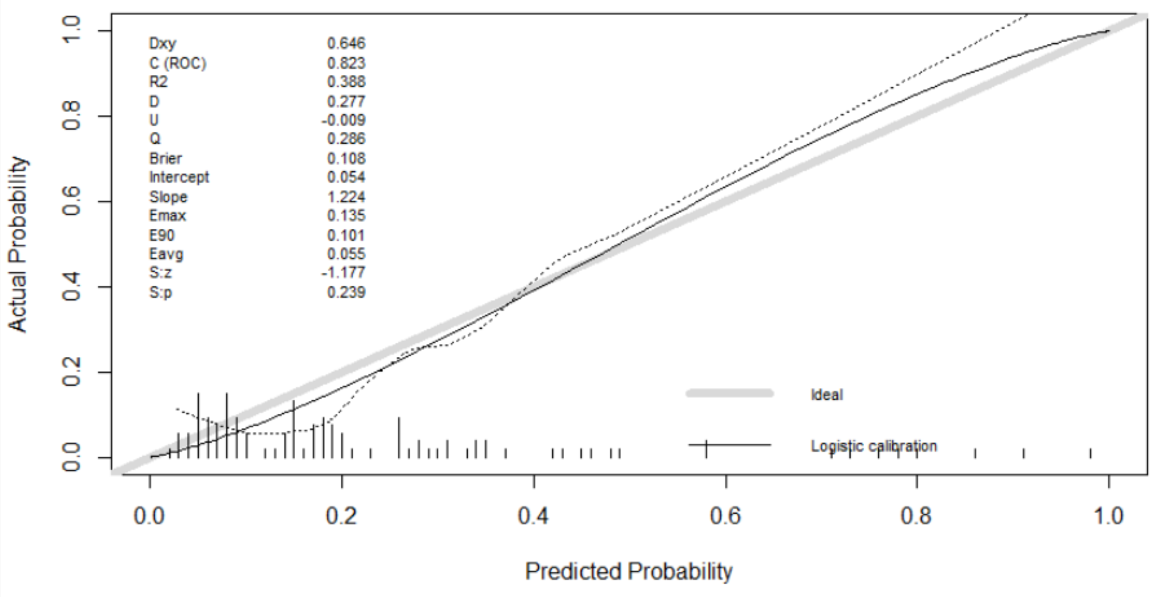

Finally, here is alternative code for obtaining a calibration plot

```
df.calibration <- data.frame(y = complete.data$y, predicted.lr =
expit(predicted.lr))
Ngroups <- 10
d1 <- quantile(df.calibration$predicted.lr, probs = seq(0, 1, 1/Ngroups))

g1<-list()
for (i in 1:Ngroups) {
  g1[[i]] <- df.calibration[df.calibration$predicted.lr >= d1[i] &
df.calibration$predicted.lr < d1[i+1],]
}

predicted <- observed <- vector(mode = "numeric", length = Ngroups)
for (i in 1:Ngroups) {
  predicted[i] <- mean(g1[[i]]$predicted.lr)
  observed[i] <- mean(g1[[i]]$y == 1)
}

dat1 <- data.frame(pred = predicted, obs = observed)
ggplot(dat1,aes(x=pred,y=obs))+geom_point(size=3,shape=20)+
  labs(x="Predicted  from LR", y="Observed") +
  geom_abline(intercept=0,slope=1,color="black",linetype="dashed",size=0.7)
+ geom_smooth(method="lm",colour="blue",size=0.7) + theme(aspect.ratio=1)
```

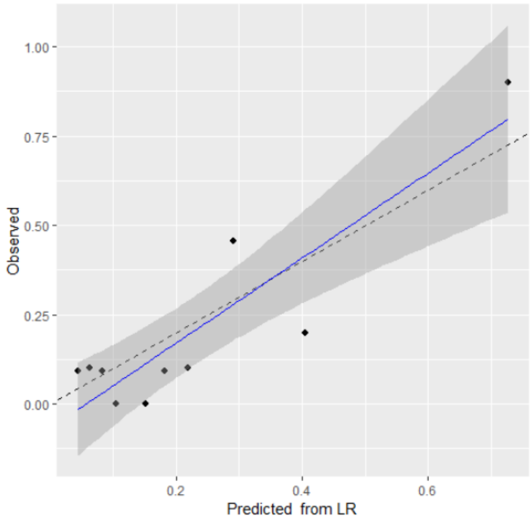

3.5 *Calculate optimism-corrected performance of the models*

We will use bootstrapping to obtain an optimism-corrected assessment of model performance, as we did in Section 2.5:

```
# Internal CV via bootstrapping -------------------
# bootstrap in each multiply imputed dataset
N.bootstrap <- 10
optimism.lr.eachbootstrap <- optimism.gam.eachbootstrap <-
optimism.ridge.eachbootstrap <- matrix(NA, N.bootstrap, 3)
optimism.lr <- optimism.gam <- optimism.ridge <- matrix(NA, n.impute, 3)

for (i in 1:n.impute){
  for(j in 1:N.bootstrap){
    boot.sample <- sample(length(imputed1[[i]]$y),replace = T)
    # create bootstrap sample
    imp.boot <- lapply(imputed1[[i]], function(x){x[boot.sample]})

    # fit spline model in bootstrap sample
    regression.splines.boot<-
lrm(y~rcs(x1,3)+rcs(x2,3)+x3+x4+x5,data=imp.boot)

    # fit gam model in bootstrap sample
    fit.gam.boot <- gam(y~x3+x4+x5+s(x1)+s(x2), data = imp.boot, family =
binomial)

    # fit ridge model in bootstrap sample
    imp <- imp.boot
    imp <- with(imp, data.frame(y, x1, x2, x3, x4, x5))
    data_glmnet <- model.matrix(y ~.,data = imp)
    data_glmnet <- data_glmnet[,-1]
    data_glmnet <- cbind(y = as.numeric(as.character(imp$y)), data_glmnet =
data_glmnet)
    X <- as.matrix(data_glmnet[,-1])
    colnames(X)[3:4] <- c("x3", "x4")
    Y <- data_glmnet[,1]
    cvfit <- cv.glmnet(X,Y,family = "binomial",alpha=0,
                       lambda = lambdas, nfolds=10)
    lambda.min <- cvfit$lambda.min
    fit.ridge.boot <- glmnet(X,Y,family = "binomial", alpha=0, lambda =
lambda.min)

    # predict in bootstrap
```

```
    f1 <- as.data.frame(do.call(cbind, lapply(imp.boot, function(x)
{as.numeric(as.character(x))})))

    boot.prediction.lr <- prediction.lr(f1, single.fit =
regression.splines.boot)
    boot.prediction.gam <- prediction.gam(f1, single.fit = fit.gam.boot)
    boot.prediction.ridge <- prediction.ridge(f1, single.fit =
fit.ridge.boot)

    lr.boot <- calculate_performance2(f1$y, boot.prediction.lr)
    gam.boot <- calculate_performance2(f1$y, boot.prediction.gam)
    ridge.boot <- calculate_performance2(f1$y, boot.prediction.ridge)

    # predict in test data
    f2 <- as.data.frame(do.call(cbind, lapply(imputed1[[i]], function(x)
{as.numeric(as.character(x))})))
    test.prediction.lr <- prediction.lr(f2, single.fit =
regression.splines.boot)
    test.prediction.gam <- prediction.gam(f2, single.fit = fit.gam.boot)
    test.prediction.ridge <- prediction.ridge(f2, single.fit =
fit.ridge.boot)

    lr.test <- calculate_performance2(f2$y, test.prediction.lr)
    gam.test <- calculate_performance2(f2$y, test.prediction.gam)
    ridge.test <- calculate_performance2(f2$y, test.prediction.ridge)

    optimism.lr.eachbootstrap[j,] <- lr.boot - lr.test
    optimism.gam.eachbootstrap[j,] <- gam.boot - gam.test
    optimism.ridge.eachbootstrap[j,] <- ridge.boot - ridge.test
  }
  optimism.lr[i,] <- apply(optimism.lr.eachbootstrap, 2, mean)
  optimism.gam[i,] <- apply(optimism.gam.eachbootstrap, 2, mean)
  optimism.ridge[i,] <- apply(optimism.ridge.eachbootstrap, 2, mean)

  print(paste0("imputation done: ", i))
}

mean.optimism.lr <- apply(optimism.lr, 2, mean)
mean.optimism.gam <- apply(optimism.gam, 2, mean)
mean.optimism.ridge <- apply(optimism.ridge, 2, mean)

optimism.corrected.lr <- apparent.lr - mean.optimism.lr
optimism.corrected.gam <- apparent.gam - mean.optimism.gam
optimism.corrected.ridge <- apparent.ridge - mean.optimism.ridge
round(rbind(optimism.corrected.lr, optimism.corrected.gam,
optimism.corrected.ridge),2)
```

	auc	calibration intercept	calibration slope
**optimism.corrected.lr**	0.78	-0.17	1.00
**optimism.corrected.gam**	0.75	-0.35	0.82
**optimism.corrected.ridge**	0.76	-1.01	1.30

Based on these results, the logistic regression with splines model seems to be the best in both calibration and discrimination.

3.6 Perform internal-external cross-validation

We follow the procedures of Section 2.6:

```
# Internal-external CV -------------------
clusters <- unique(data.bin$clust)
N.clust <- length(clusters) # 5 clusters in this example
data.in <- data.leftout <- list()
```

```
#create the datasets
for(i in 1:N.clust){
  data.in[[i]]<-data.bin[data.bin$clust!=clusters[i],]
  data.leftout[[i]]<-data.bin[data.bin$clust==clusters[i],]
  complete.index <- complete.cases(data.leftout[[i]][,c(paste0("x", 1:5),
"y")])
  data.leftout[[i]] <- data.leftout[[i]][complete.index,]
}

n.impute <- 10

imputed <- regression.splines.CV <- fit.gam.CV <- fit.ridge.CV <- list()
leftout.prediction.lr <- leftout.prediction.gam <- leftout.prediction.ridge
<- list()
leftout.performance.lr <- leftout.performance.gam <-
leftout.performance.ridge <- list()

for (i in 1:N.clust){
  a <- aregImpute(data=data.in[[i]], I(y)~x1+x2+I(x3)+I(x4)+I(x5),
n.impute=n.impute, nk=3, match='closest')

  for (j in 1:n.impute){
    imputed[[j]] <- impute.transcan(a, imputation=j, data=data.in[[i]],
list.out=TRUE, pr=FALSE, check=FALSE)
    regression.splines.CV[[j]]<-
lrm(y~rcs(x1,3)+rcs(x2,3)+x3+x4+x5,data=imputed[[j]])
    fit.gam.CV[[j]] <- gam(y ~ x3+x4+x5+s(x1)+s(x2), data = imputed[[j]],
family = binomial)

    imp <- with(imputed[[j]], data.frame(y, x1, x2, x3, x4, x5))
    data_glmnet <- model.matrix(y ~.,data = imp)
    data_glmnet <- data_glmnet[,-1]
    data_glmnet <- cbind(y = as.numeric(as.character(imp$y)), data_glmnet =
data_glmnet)
    X <- as.matrix(data_glmnet[,-1])
    colnames(X)[3:4] <- c("x3", "x4")
    Y <- data_glmnet[,1]
    cvfit <- cv.glmnet(X,Y,family = "binomial",alpha=0,
                       lambda = lambdas, nfolds=10)
    lambda.min <- cvfit$lambda.min
    fit.ridge.CV[[j]] <- glmnet(X,Y,family = "binomial", alpha=0, lambda =
lambda.min)
  }

  leftout.prediction.lr[[i]] <- prediction.lr(data.leftout[[i]],
multiple.fit = regression.splines.CV)
  leftout.prediction.gam[[i]] <- prediction.gam(data.leftout[[i]],
multiple.fit = fit.gam.CV)
  leftout.prediction.ridge[[i]] <- prediction.ridge(data.leftout[[i]],
multiple.fit = fit.ridge.CV)

  leftout.performance.lr[[i]] <-
calculate_performance2(data.leftout[[i]]$y, leftout.prediction.lr[[i]])
  leftout.performance.gam[[i]] <-
calculate_performance2(data.leftout[[i]]$y, leftout.prediction.gam[[i]])
  leftout.performance.ridge[[i]] <-
calculate_performance2(data.leftout[[i]]$y, leftout.prediction.ridge[[i]])
}

#performance per cluster
```

```
#leftout.performance.lr
#leftout.performance.gam
#leftout.performance.ridge

# performance aggregating all cluster
leftout.prediction.lr.agg <- do.call(c, leftout.prediction.lr)
leftout.prediction.gam.agg <- do.call(c, leftout.prediction.gam)
leftout.prediction.ridge.agg <- do.call(c, leftout.prediction.ridge)
IECV.observed <- do.call(rbind, data.leftout)$y
IECV.cluster <- do.call(rbind, data.leftout)$clust

IECV.lr <- calculate_performance2(IECV.observed, leftout.prediction.lr.agg)
IECV.gam <- calculate_performance2(IECV.observed,
leftout.prediction.gam.agg)
IECV.ridge <- calculate_performance2(IECV.observed,
leftout.prediction.ridge.agg)
round(rbind(IECV.lr, IECV.gam, IECV.ridge),2)
```

```
            auc calibration intercept calibration slope
IECV.lr    0.74                 -0.33              0.78
IECV.gam   0.75                 -0.54              0.62
IECV.ridge 0.73                 -0.85              1.06
```

We can also see the performance of a model when predicting in each of the left-out clusters, to get a sense about the expected heterogeneity of the performance of the prediction model in a new setting:

```
auc.clusters=data.frame(auc=rep(NA,N.clust), SE=NA, cluster=NA)
for(i in 1:N.clust){
  d.cl<-Int.ext.complete[Int.ext.complete$cluster==clusters[i],]
  roc1<-roc(d.cl$observed,d.cl$predicted.ridge)
  auc.clusters$auc[i]<-auc(roc1)
  auc.clusters$SE[i]<-(ci(roc1)[3]-ci(roc1)[1])/3.92
  auc.clusters$cluster[i]<-clusters[i]
}
round(auc.clusters,digits=2)
```

```
   auc   SE cluster
1 0.62 0.25       1
2 0.88 0.07       2
3 0.79 0.13       3
4 0.60 0.21       4
5 0.72 0.15       5
```

Finally we can create a forestplot for AUC using **`meta`**:[12]

```
library(meta)
meta.AUC<-metagen(TE=auc, seTE=SE, studlab = cluster, data=auc.clusters)
forestplot<-forest(meta.AUC, prediction = T, xlim=c(0.4,1),
           colgap.left="5mm", rightcols = c("effect", "ci"),
           leftlabs = c("Cluster", "AUC", "seTE"))
```

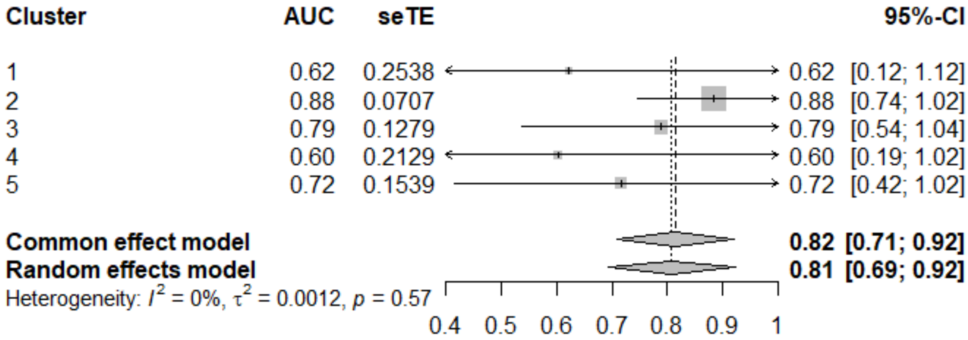

3.7 Decision curve analysis

We perform a decision curve analysis (DCA) using the complete dataset, via the **`dcurves`** package.[13]

```
# plot a decision curve analysis
# see also https://cran.r-
project.org/web/packages/dcurves/vignettes/dca.html

library(dcurves)
for.dca=data.frame(obs=as.numeric(complete.data$y)-1,
                   predicted.gam=expit(predicted.gam),
                   predicted.lr=expit(predicted.lr),
predicted.ridge=expit(predicted.ridge))
for.dca=data.frame(obs=as.numeric(complete.data$y)-1,
                   predicted.gam=expit(predicted.gam),
predicted.ridge=expit(predicted.ridge),
                   predicted.lr=expit(predicted.lr))
dca1<-dca(obs~predicted.lr+predicted.ridge+predicted.gam, data=for.dca)
plot(dca1, smooth = TRUE)
```

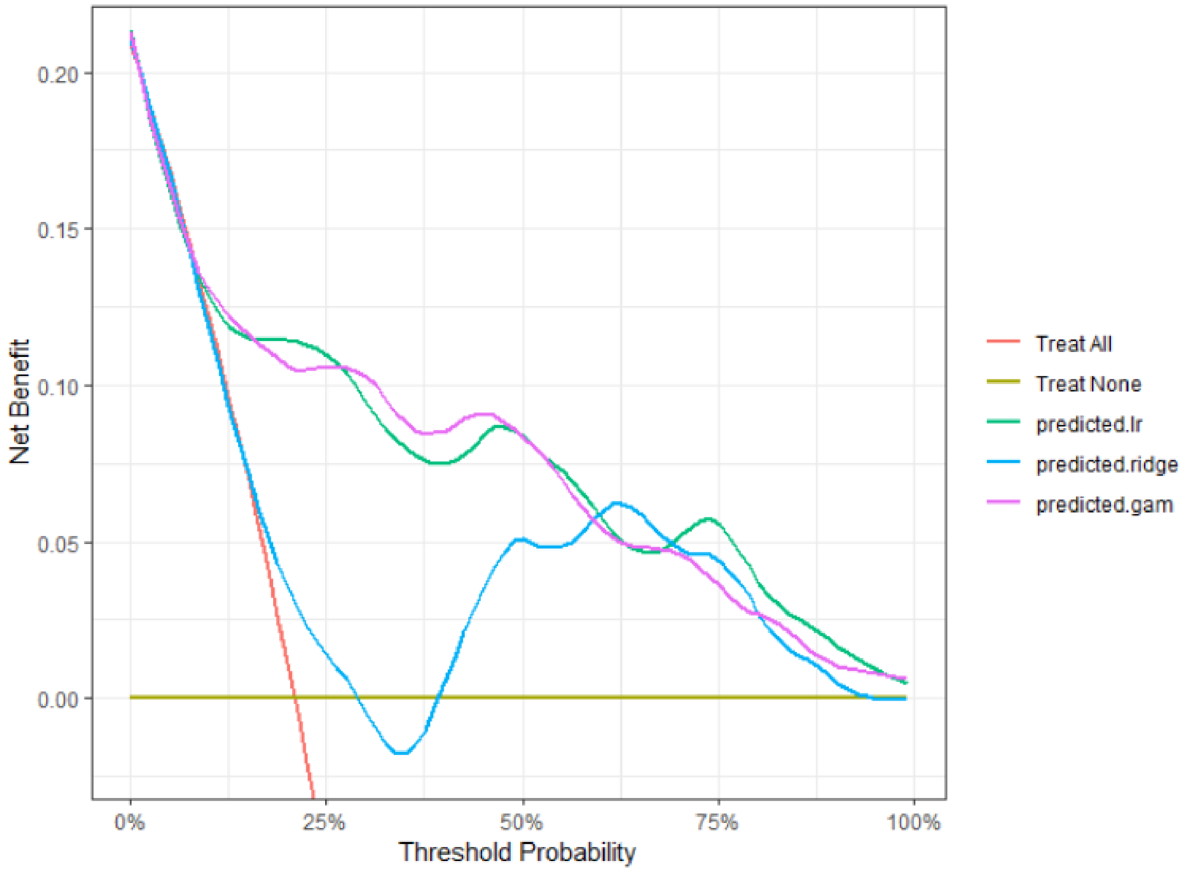

We see again that the ridge model is underperforming. We also see that for a threshold probability of ~10% or less, the treat all strategy is equally beneficial to using our models. Of note, the decision curve analysis presented in this section used in-sample predictions (i.e. it may be prone to overfitting). One way to correct for this is again bootstrapping or cross-validation;[14,15] however, we do not apply this method here.

4 Example for time-to-event outcomes

4.1 Simulate a toy example

We follow the methods of Section 2.1 to generate an example dataset. Here we will generate 5 covariates `x1, x2, x3, x4, x5,` and five auxiliary variables `z1, z2, z3, z4, z5,` a clustering variable `clust,` the `time` of follow-up for each patient and the censoring `status` (0 or 1).

```
remove(list=ls())
set.seed(42) # the answer to life the universe and everything

# simulate data
library(MASS)
N <- 500
Sigma <- outer(1:10, 1:10, function(x,y) 0.5^abs(x-y)) #variance covariance
matrix for covariates

x <- mvrnorm(n = N, rep(0, 10), Sigma)
x[,3] <- ifelse(x[,3] > 0.5, 1, 0) #binary predictor
x[,4] <- ifelse(x[,4] > 0, 1, 0) #binary predictor
x[,5] <- ifelse(x[,5] > 1, 1, 0) #binary predictor
x[,8] <- ifelse(x[,8] > 2, 1, 0) #binary auxiliary variable
x[,9] <- ifelse(x[,9] > 2, 1, 0) #binary auxiliary variable
x[,10] <- cut(x[,10], breaks=c(-Inf, -1, 1, 2, Inf)) #categorical with 4
categories

survdat.compl <- data.frame(x)
colnames(survdat.compl) <- paste0("x", 1:10)
rate <- with(survdat.compl,
exp(x1+0.4*x1^2+0.4*x2+0.05*x2^2+(x3==2)+0.5*(x4==2)-
            0.5*(x5==1)+rnorm(N,0,0.1))/10)
survdat.compl[,c(3:5, 8:10)] <- lapply(survdat.compl[,c(3:5, 8:10)],
factor)

fulltime <- rexp(N, rate = rate)
censtimes <- 5 + 20*runif(N)
mean(censtimes)
survdat.compl$time <- pmin(fulltime, censtimes)
survdat.compl$status <- as.numeric(censtimes > fulltime)
colnames(survdat.compl)[6:10]=c("z1","z2" ,"z3", "z4", "z5")
table(survdat.compl$status)
```

```
  0   1
137 363
```

i.e. 137 patients were censored while 363 experienced the event.

Next, we introduce missing data in the covariates and we create a clustering variable:

```
# introduce missing data for the covariates
missing.matrix=matrix(0, nrow=nrow(survdat.compl), ncol=10)
missing.matrix=matrix(rbinom(length(missing.matrix),1, p=0.1),
nrow=nrow(survdat.compl))
survdat=survdat.compl[,1:10]
survdat[missing.matrix==1]=NA
survdat=cbind(survdat, survdat.compl[11:12])

# create clusters
survdat$clust <- factor(sample(1:5, size = N, replace = TRUE, prob =
rep(0.2,5)))
survdat <- survdat[order(survdat$clust),]
head(survdat)
```

```
x1          x2 x3 x4    x5           z1          z2   z3 z4   z5        time status clust
10  1.5967150         NA  0  0 <NA>  0.38452856  1.0118199    0  0    2  1.0591365      1     1
14 -0.7489477 -0.4119789  0  1    0 -0.04344516 -0.1876478    0  0    2 13.2169852      1     1
18  1.7327055 -0.6244390  0  1    1          NA  2.7708399 <NA>  0 <NA>  0.2686032      1     1
27 -1.4119981 -0.2056145  0  0    0  1.15318721  0.6450764    0  0    4 16.8137300      0     1
29  0.6733386  0.3010077  0  0 <NA>  0.65092121  0.9063692    0  0    1  5.0152636      1     1
35 -0.5702342 -0.6440433  0  0    0 -0.51055435  0.3857109    0  0    2  9.8360372      0     1
```

4.2 Visualize data

Before commencing with the analyses, we can visualize the data, starting with a Kaplan-Meier curve. We use **`ggplot2`**, **`ggfortify`**, and **`survival`** here:[3,16,17]

```
# KM curve
library(ggplot2)
library(ggfortify)
library(survival)
model_fit <- survfit(Surv(time, status) ~ 1, data=survdat)

autoplot(model_fit) +
  labs(x = "\n Survival Time (Days) ", y = "Survival Probabilities \n",
       title = "Survival Times") +
  theme(plot.title = element_text(hjust = 0.5),
        axis.title.x = element_text(face="bold", size = 12),
        axis.title.y = element_text(face="bold", size = 12),
        legend.title = element_text(face="bold", size = 10))
```

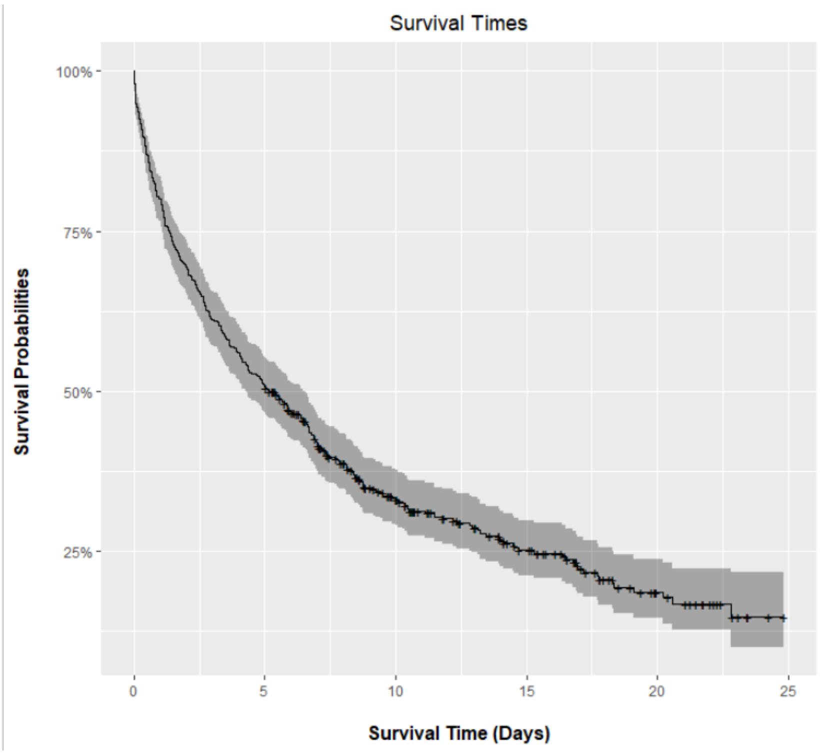

We can also plot for different levels of the covariates, e.g. for different values of `x5`:

```
### plot survival curves for specific levels of covariates
model_fit2 <- survfit(Surv(time, status) ~ x5, data=survdat)
autoplot(model_fit2) +
  labs(x = "\n Survival Time (Days) ", y = "Survival Probabilities \n",
       title = "Survival Times") +
  theme(plot.title = element_text(hjust = 0.5),
        axis.title.x = element_text(face="bold", size = 12),
        axis.title.y = element_text(face="bold", size = 12),
        legend.title = element_text(face="bold", size = 10))+ xlim(0,30)
```

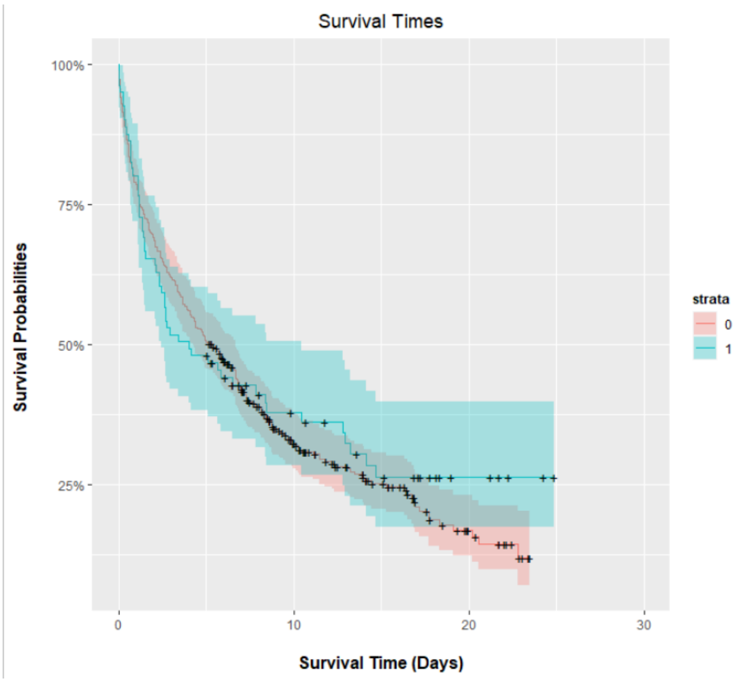

4.3 *Perform multiple imputations*

The data has several missing entries, so we need to perform multiple imputations. Following White and Royston,[18] for the imputation we will use the cumulative baseline hazard estimated by the Nelson–Aalen estimator, in addition to other covariates:

```
# Impute missing data
library(Hmisc)
n.impute <- 10
survdat$nelsonaalen <- nelsonaalen(survdat, time, status)
a <- aregImpute(data=survdat, ~status+x1+x2+I(x3)+I(x4)+I(x5)+
                  z1+z2+I(z3)+I(z4)+I(z5)+clust+nelsonaalen,
n.impute=n.impute, nk=3, match='closest')

# get imputed datasets
imputed <- list()
for (i in 1:n.impute){
  imputed[[i]] <- impute.transcan(a, imputation=i, data=survdat,
list.out=TRUE, pr=FALSE, check=FALSE)
  imputed[[i]]$time<-survdat$time
}
```

4.4 *Fit a prediction model*

Having created the imputed dataset, we can now fit a prediction model. We hereby use a Weibull model with smoothing splines for the continuous predictors:

```
# Weibull model with smoothing splines
fit.model <- list()
for(i in 1:n.impute){
  fit.model[[i]] <- survreg(Surv(time, status)~pspline(x1,
df=2)+pspline(x2, df=2)+ridge(x3,x4, scale=T),
                            imputed[[i]], dist="weibul")
}
```

We can use this model to make predictions for a new patient and draw the expected survival curve.

```
# predict for new patient
new.patient <- data.frame(x1 = 0.5, x2 = 0.2, x3 = as.factor(1), x4 =
as.factor(2))
prediction.weibull.time <- function(new.patient, fit.model = NULL){
  pct <- 1:99/100
  predicted.exp <- list()
  for(i in 1:n.impute){
ptime <- predict(fit.model[[i]], newdata=new.patient, type='quantile',
p=pct, se=TRUE)
    predicted.exp[[i]] <- data.frame(surv=pct,
pred=ptime$fit,var=ptime$se.fit^2)
  }

  predicted.exp.average <- Reduce("+", predicted.exp) / n.impute
  means <- predicted.exp.average[,2]
  U <- predicted.exp.average[,3]

  errors <- list()
  for(i in 1:n.impute){
    errors[[i]] <- (predicted.exp[[i]]$pred-means)^2
  }
  B <- 1/(n.impute-1)*(Reduce("+", errors))
  varMI <- U + (1+1/n.impute)*B
  results <- data.frame("percentiles.surv"=pct,"means"=means, "sd" =
sqrt(varMI),
                        "lowerCI"=means-1.96*sqrt(varMI),
"upperCI"=means+1.96*sqrt(varMI))
  return(results)
}

predictions.time <- prediction.weibull.time(new.patient, fit.model)

ggplot(predictions.time, aes(x = means, y =1 - percentiles.surv )) +
  geom_line(col="black") +
  geom_ribbon(aes(xmin = lowerCI, xmax = upperCI),
              alpha=0.1,
              linetype="dashed",
              color="grey")+xlab("days")+ylab("Survival probability")
+xlim(0,30)
```

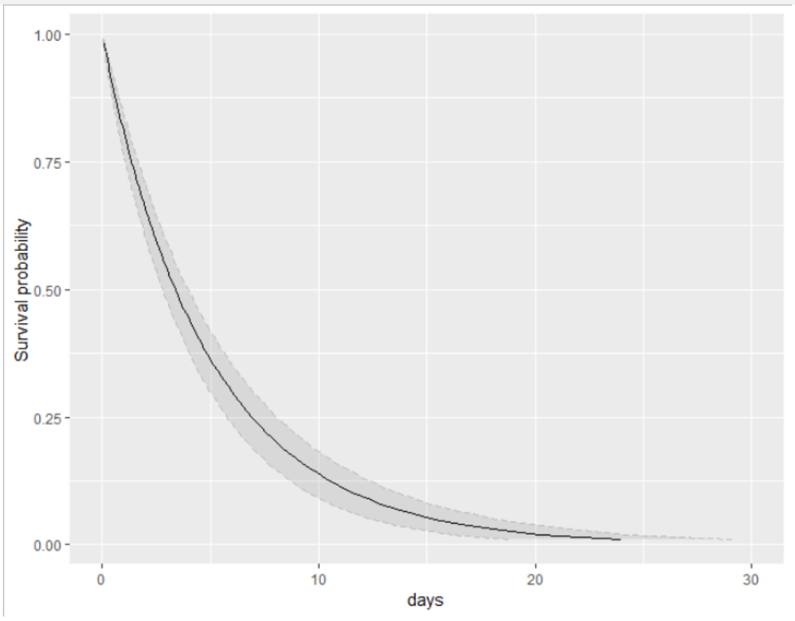

We can also calculate the so-called linear predictor, which we will use in the next sections to assess of model performance:

```
# calculate the linear predictor
prediction.weibull <- function(new.patient, single.fit = NULL, multiple.fit
= NULL){
  if(!is.null(multiple.fit)){

    ff <- function(i){
      predict(multiple.fit[[i]], newdata = new.patient, type = "lp")
    }
    prediction_matrix <- sapply(1:length(multiple.fit), ff)

    if(dim(new.patient)[1] == 1){
      prediction <- mean(prediction_matrix)
    } else{
      prediction <- apply(prediction_matrix, 1, mean)
    }

  } else if(!is.null(single.fit)){
    prediction <- predict(single.fit, newdata = new.patient, type = "lp")
  }
  return(prediction)
}
complete.data <- survdat[complete.cases(survdat[,1:5]),]
predicted.weibull <- prediction.weibull(complete.data, multiple.fit =
fit.model)
```

4.5 Calculate apparent performance of the model

As was the case for the binary outcome, we need to assess both calibration and discrimination of a new model. Discrimination is usually assessed via Harrell's c-statistic,[19] or using the modification proposed by Uno et al.[20] Calibration can be estimated by refitting a simple Cox model with the linear predictor of the model as the only covariate:

```
# Assess apparent performance (no optimism correction) -------------------
calculate_performance <- function(time = NULL, status = NULL, lp = NULL){

  #discrimination
  harrell_C <- concordance(Surv(time, status) ~ lp)
  harrell_C_est <- harrell_C$concordance
  #harrell_C_var <- harrell_C$var

  Uno_C <- concordance(Surv(time, status) ~ lp, timewt = "n/G2")
  Uno_C_est <- Uno_C$concordance
  #Uno_C_var <- Uno_C$var

  #calibration
  second_model <- survreg(Surv(time, status) ~ lp, dist="weibul")
  calslope <- second_model$coef[2]

  returnVec <- c(harrell_C_est, Uno_C_est, calslope)
  names(returnVec) <- c("Harrel_C", "Uno_C", "calibration.slope")

  return(returnVec)
}
apparent.weibull <- calculate_performance(time = complete.data$time, status
= complete.data$status,lp = predicted.weibull)
round(apparent.weibull,2)
```

```
Harrel_C          Uno_C calibration.slope
0.76              0.75              1.07
```

We can also assess calibration at a specific time point by comparing observed vs. predicted events. We use here the code provided by McLernon et al.[21], after adapting it for multiply imputed datasets:

```
# calibration in the large
timepoint <- 5
# Observed
obj <- summary(survfit(
  Surv(time, status) ~ 1,
  data = complete.data),
  times = timepoint)
obs_t <- 1 - obj$surv

# Predicted risk
prediction.weibull2 <- function(new.patient, fit.model = NULL, time.point =
NULL){

  if(is.null(time.point)){
    pct <- 10*1:300/100
  } else{
    pct <- time.point
  }
  predicted.exp <- list()
  for(i in 1:n.impute){
    mu_hat <- predict(fit.model[[i]], newdata = new.patient, type = "lp")
    predicted.exp[[i]] <- 1 - pweibull(pct, shape = 1/fit.model[[i]]$scale,
                                       scale = exp(mu_hat))
  }
  predicted.exp.average <- Reduce("+", predicted.exp) / n.impute

  results <- data.frame(time = pct, means = predicted.exp.average)
  return(results)
}

predictions <- list()
for (i in 1:dim(complete.data)[1]){
  predictions[[i]] <- prediction.weibull2(complete.data[i,], fit.model,
time.point = timepoint)$means
}
est.surv <- unlist(predictions)
pred <- 1 - est.surv

# Expected
exp_t <- mean(pred)
OE_t <- obs_t / exp_t

alpha <- .05
OE_summary <- c(
  "OE" = OE_t,
  "2.5 %" = OE_t * exp(-qnorm(1 - alpha / 2) * sqrt(1 / obj$n.event)),
  "97.5 %" = OE_t * exp(+qnorm(1 - alpha / 2) * sqrt(1 / obj$n.event))
)
round(OE_summary,2)
    OE  2.5 % 97.5 %
  1.02   0.87   1.19
```

We can inspect calibration graphically:

```
# drawing a calibration plot
library(rms)
predictions <- list()
for (i in 1:dim(complete.data)[1]){
  predictions[[i]] <- prediction.weibull2(complete.data[i,], fit.model,
time.point = complete.data[i,]$time)$means
}
est.surv <- unlist(predictions)
f <- val.surv(S= Surv(complete.data$time, complete.data$status),
              est.surv = est.surv)
plot(f, xlab="predicted survival", ylab="observed survival")
```

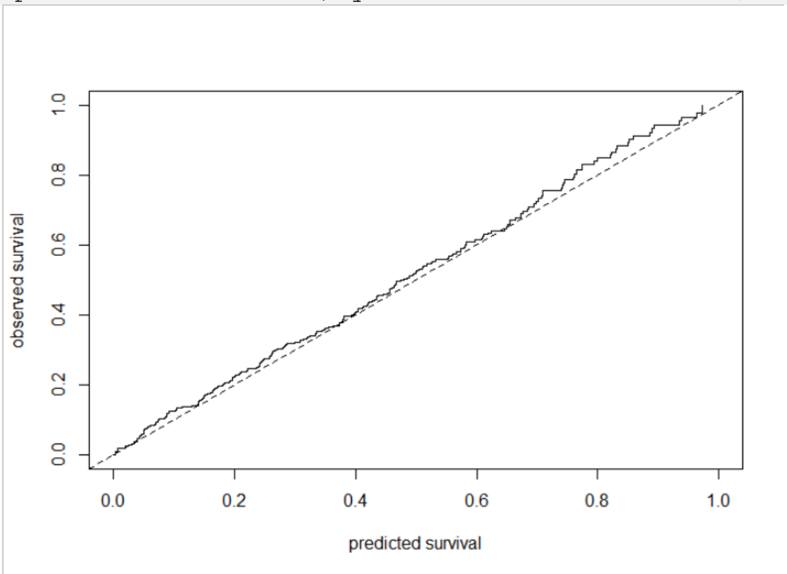

We can also assess calibration by checking the observed (i.e. Kaplan Meier) survival with the one estimated by the model.

```
# drawing calibration plot 2
# calibration plot overall
prediction.weibull2 <- function(new.patient, fit.model = NULL){
  pct <- 10*1:300/100
  predicted.exp <- list()
  for(i in 1:n.impute){
    mu_hat <- predict(fit.model[[i]], newdata = new.patient, type = "lp")
    predicted.exp[[i]] <- 1 - pweibull(pct, shape = 1/fit.model[[i]]$scale,
                                       scale = exp(mu_hat))
  }
  predicted.exp.average <- Reduce("+", predicted.exp) / n.impute

  results <- data.frame(time = pct, means = predicted.exp.average)
  return(results)
}

predictions <- list()
for (i in 1:dim(complete.data)[1]){
  predictions[[i]] <-
prediction.weibull2(complete.data[i,],fit.model)$means
}

predictions.mean <- apply(do.call(cbind, predictions), 1, mean)

m2 <- survfit(Surv(time, status) ~ 1, data= complete.data)
res <- summary(m2, censored = T)
dt1 <- with(res, data.frame(time = time, surv = surv, upper = upper,
```

```
                             lower = lower))
dt3 <- data.frame(time = c(10*1:300/100, dt1$time),
               surv = c(predictions.mean, dt1$surv),
               group = c(rep("pred",300), rep("obs",length(dt1$surv))))

ggplot(data = dt3) +
  geom_line(aes(x = time, y = surv, group = group, colour = group,
                linetype = group, size = group)) +
  scale_size_manual(values = c(0.5,0.1)) +
  scale_color_manual(values = c("red", "black")) +
  scale_linetype_manual(values = c("solid", "dashed")) +
  xlim(0,30)
```

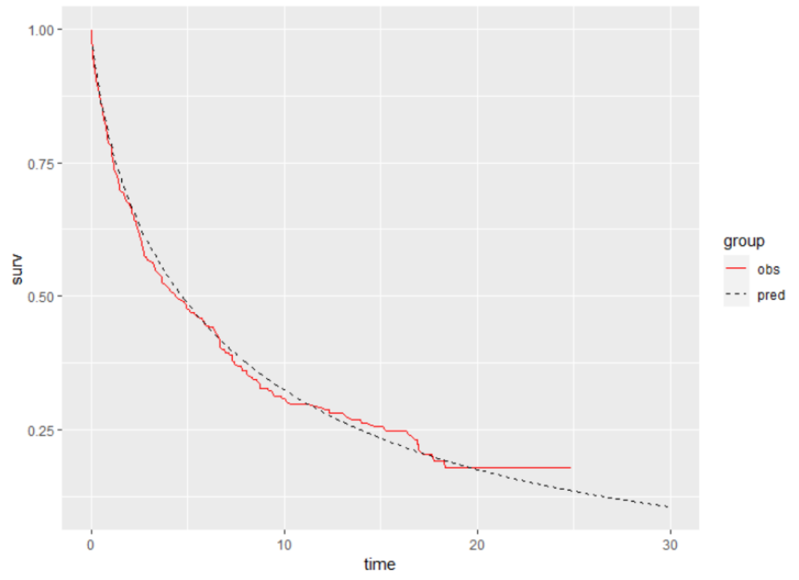

The red line above is the Kaplan Meier estimate, the dotted line is the predicted survival from the model. Additionally, we can plot by levels of covariates, to also assess discrimination.

```
# calibration plot by levels of covariates
group1 <- complete.data[complete.data$x3 == 0,]
group2 <- complete.data[complete.data$x3 == 1,]

predictions.group1 <- list()
for (i in 1:dim(group1)[1]){
  predictions.group1[[i]] <-
prediction.weibull2(group1[i,],fit.model)$means
}

predictions.group2 <- list()
for (i in 1:dim(group2)[1]){
  predictions.group2[[i]] <-
prediction.weibull2(group2[i,],fit.model)$means
}

predictions.mean.group1 <- apply(do.call(cbind, predictions.group1), 1,
mean)
predictions.mean.group2 <- apply(do.call(cbind, predictions.group2), 1,
mean)

predictions.both.groups <- data.frame(means=
c(predictions.mean.group1,predictions.mean.group2),
```

```
                                  group= c(rep("x3=1, fitted", 300),
rep("x3=2, fitted", 300)), percentiles.surv = 1:300/100)

m2 <- survfit(Surv(time, status) ~ x3, data=
complete.data[complete.cases(complete.data[,1:5]),])
res <- summary(m2, censored = T)
dt1 <- with(res, data.frame(time = time, surv = surv, upper = upper,
                           lower = lower, strata=strata))

dt2 <- data.frame(time = c(10*1:300/100, 10*1:300/100, dt1$time),
               surv = c(predictions.both.groups$means, dt1$surv),
               group = c(predictions.both.groups$group, dt1$strata))
dt2$group[dt2$group == "1"] <- "x3=1, observed"
dt2$group[dt2$group == "2"] <- "x3=2, observed"

ggplot(data = dt2) +
  geom_line(aes(x = time, y = surv, group = group, colour=group,
linetype=group,
                size = group)) +
  scale_size_manual(values=c(0.8,0.1,0.8,0.1))+
  scale_color_manual(values=c("red", "red", "black", "black"))+
  scale_linetype_manual(values=c("solid", "dashed","solid", "dashed"))+
  xlim(0,25)
```

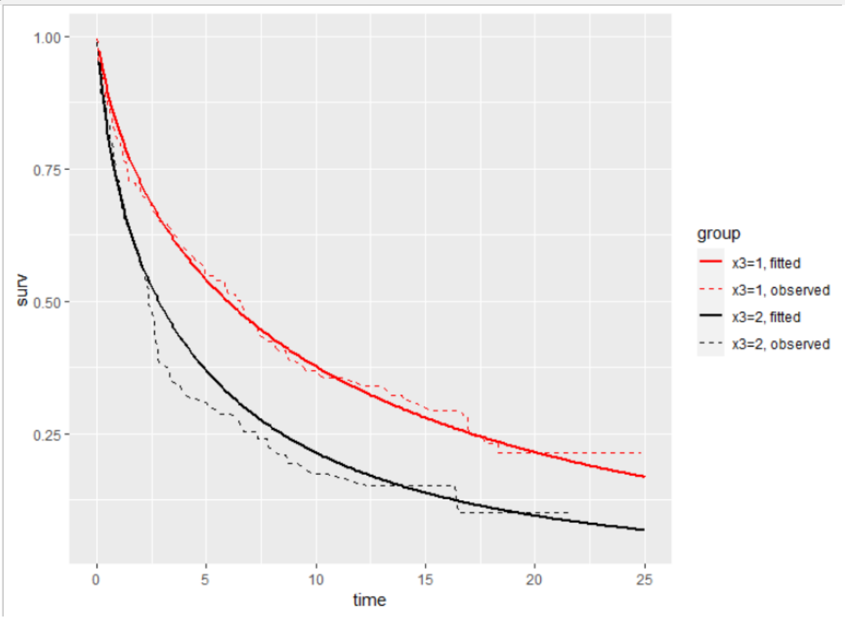

4.6 *Calculate optimism-corrected performance of the models*

We will use bootstrapping on the imputed datasets to obtain an optimism-corrected assessment of model performance, as we did in Section 2.5:

```
# Internal CV via bootstrapping -------------------
# bootstrapping in each multiply imputed dataset
n.bootstrap <- 10
n.impute <- 10
optimism.weibull.eachbootstrap <-  matrix(NA, n.bootstrap, 3)
optimism.weibull <- matrix(NA, n.impute, 3)

for (i in 1:n.impute){
  for(j in 1:n.bootstrap){

    boot.sample <- sample(length(imputed[[i]]$time), replace = T)
```

```
    # create bootstrap sample
    imp.boot <- lapply(imputed[[i]], function(x){x[boot.sample]})

    # fit spline model in bootstrap sample
    fit.weibull.boot <- survreg(Surv(time, status)~pspline(x1,
df=2)+pspline(x2, df=2)+ridge(x3,x4, scale=T),imp.boot, dist="weibul")

    # predict in bootstrap
    f1 <- as.data.frame(do.call(cbind, lapply(imp.boot, function(x)
{as.numeric(as.character(x))})))
    boot.prediction.weibull <- prediction.weibull(f1, single.fit =
fit.weibull.boot)

    weibull.boot <- calculate_performance(time = f1$time, status =
f1$status, lp = boot.prediction.weibull)

    # predict in test data
    f2 <- as.data.frame(do.call(cbind, lapply(imputed[[i]], function(x)
{as.numeric(as.character(x))})))
    test.prediction.weibull <- prediction.weibull(f2, single.fit =
fit.weibull.boot)

    weibull.test <- calculate_performance(time = f2$time, status =
f2$status, lp = test.prediction.weibull)

    optimism.weibull.eachbootstrap[j,] <- weibull.boot - weibull.test
  }
  optimism.weibull[i,] <- apply(optimism.weibull.eachbootstrap, 2, mean)

  print(paste0("imputation done: ", i))
}

mean.optimism.weibull <- apply(optimism.weibull, 2, mean)
optimism.corrected.weibull <- apparent.weibull - mean.optimism.weibull
optimism.corrected.weibull
Harrel_C           Uno_C calibration.slope
    0.76            0.75              1.06
```

Essentially we see no optimism here. This was expected since the dataset is large, there were many events, and the model had few parameters.

4.7 Perform internal-external cross-validation

We perform an internal-external cross-validation using the `clust` variable, as in Section 2.6:

```
# Internal-external CV -------------------
clusters <- unique(survdat$clust)
N.clust <- length(clusters) # 5 clusters in this example
data.in <- data.leftout <- list()

#create the datasets
for(i in 1:N.clust){
  data.in[[i]]<- survdat[survdat$clust!=clusters[i],]
  data.leftout[[i]]<- survdat[survdat$clust==clusters[i],]
  complete.index <- complete.cases(data.leftout[[i]][,c(paste0("x", 1:5))])
  data.leftout[[i]] <- data.leftout[[i]][complete.index,]
}

n.impute <- 10
imputed <- fit.weibull.CV <- list()
```

```
leftout.prediction.weibull <- leftout.performance.weibull <- list()

for (i in 1:N.clust){

  data.in[[i]]$nelsonaalen <- nelsonaalen(data.in[[i]], time, status)
  meth <- make.method(data.in[[i]])
  pred <- make.predictorMatrix(data.in[[i]])
  pred[,"time"] <- 0 # don't include time variable in the
imputation;instead we have baseline hazard
  imp.surv <- mice(data.in[[i]], m = n.impute)

  imputed <- list()
  impc <- complete(imp.surv, action="long")
  for(j in 1:n.impute){
    imputed[[j]] <- impc[impc$.imp==j, c(3:7, 13:15)]
  }

  for (j in 1:n.impute){
    fit.weibull.CV[[j]] <- survreg(Surv(time, status)~pspline(x1,
df=2)+pspline(x2, df=2)+ridge(x3,x4, scale=T),
                                  imputed[[j]], dist="weibul")
  }

  leftout.prediction.weibull[[i]] <- prediction.weibull(data.leftout[[i]],
multiple.fit = fit.weibull.CV)

  leftout.performance.weibull[[i]] <- calculate_performance(time =
data.leftout[[i]]$time, status = data.leftout[[i]]$status,
                                        lp = leftout.prediction.weibull[[i]])
}

# performance per cluster
IECV.weibull=data.frame(t(leftout.performance.weibull[[1]]))
for( i in 2:N.clust){IECV.weibull=rbind(IECV.weibull,
t(leftout.performance.weibull[[i]]))}
IECV.weibull$cluster=1:N.clust
round(IECV.weibull,2)
  Harrel_C Uno_C calibration.slope cluster
1     0.75  0.74              1.22       1
2     0.79  0.78              1.28       2
3     0.73  0.74              0.83       3
4     0.78  0.77              1.08       4
5     0.73  0.70              0.98       5
```

4.8 *Decision curve analysis*

We will use the complete dataset for this analysis. First, we need to set a time point of interest. Then we need to use the models developed in the imputed datasets to predict the probability of an event at this time point, for each patient in the complete dataset.

```
### decision curve analysis -----
library(dcurves)
timepoint<-10
ptime <- predict(fit.model[[i]], newdata=complete.data, type='quantile',
p=10, se=TRUE)

pred.complete=matrix(0, nrow=dim(complete.data)[1], ncol=n.impute)
for(i in 1:n.impute){
  mu_hat <- predict(fit.model[[i]], newdata = complete.data, type = "lp")
  pred.complete[,i]= pweibull(timepoint, shape = 1/fit.model[[i]]$scale,
                    scale = exp(mu_hat))}
```

```
pred.complete2=rowMeans(pred.complete)

for.dca=data.frame(time=complete.data$time, status=complete.data$status,
pred=pred.complete2)
dca1<-dca(Surv(time, status) ~ pred,
          data = for.dca,thresholds = 1:80 / 100,
          time = timepoint)
plot(dca1,smooth = TRUE)
```

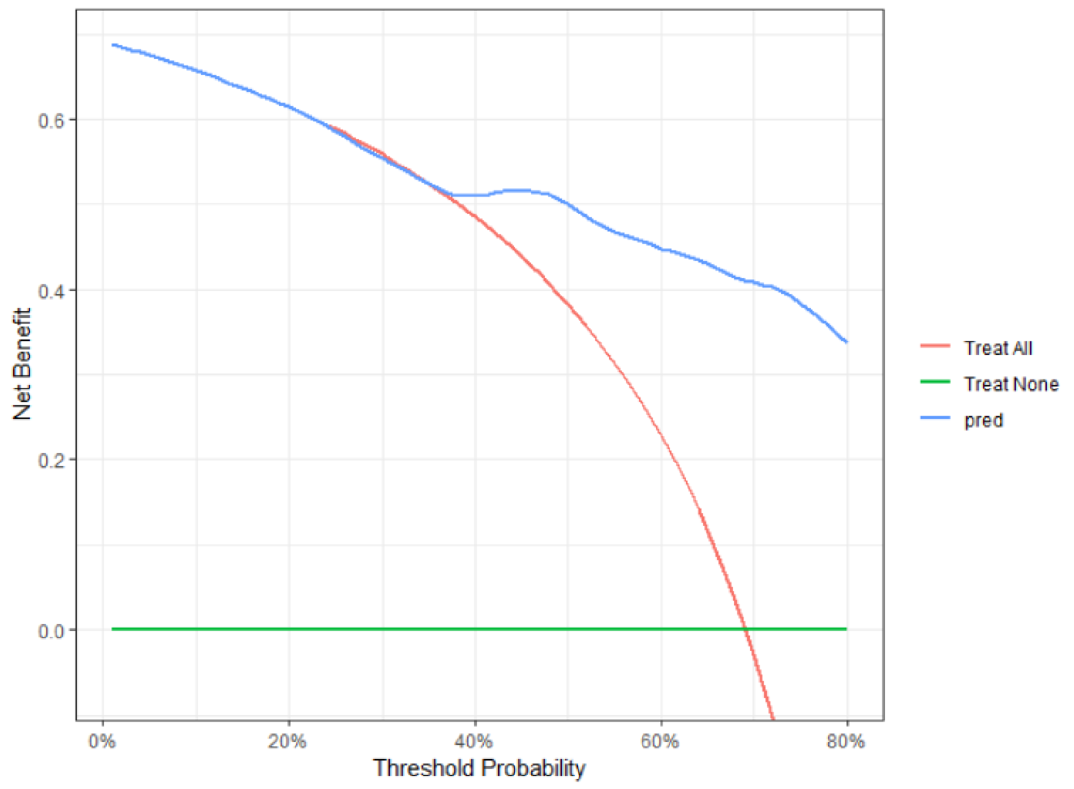

We see that the model is only useful for a threshold probability of around 40% or more. See also the note in Section 3.7 about overfitting.

5 Example for competing risks outcomes

Here we will reuse code provided by van Geloven et al.[22] after adjusting it for multiple imputations.

5.1 *Simulate a toy example*

We will first simulate a toy example, assuming there are 2 competing events. We will generate again 10 covariates (5 predictors, 5 auxiliary variables) `x1, x2, x3, x4, x5, z1, z2, z3, z4, z5,` a clustering variable `clust,` the `time` of follow-up for each patient and the type of an event, i.e. `status`, which can be 0 (for patients that did not have an event by the end of their longest follow-up), 1, and 2 (for patients having event type 1 or 2 respectively).

```
# parts of the code copied from https://github.com/survival-
lumc/ValidationCompRisks/blob/main/Prediction_CSC_minimal.R
# Original paper https://www.bmj.com/content/bmj/377/bmj-2021-
069249.full.pdf

remove(list=ls())
set.seed(42) # the answer to life the universe and everything

## simulate data ------------
library(MASS)
N <- 500
Sigma <- outer(1:10, 1:10, function(x,y) 0.5^abs(x-y)) #variance covariance
matrix for covariates

x <- mvrnorm(n = N, rep(0, 10), Sigma)
x[,3] <- ifelse(x[,3] > 0.5, 1, 0) #binary predictor
x[,4] <- ifelse(x[,4] > 0, 1, 0) #binary predictor
x[,5] <- ifelse(x[,5] > 1, 1, 0) #binary predictor
x[,8] <- ifelse(x[,8] > 2, 1, 0) #binary auxiliary variable
x[,9] <- ifelse(x[,9] > 2, 1, 0) #binary auxiliary variable
x[,10] <- cut(x[,10], breaks=c(-Inf, -1, 1, 2, Inf)) #categorical with 4
categories

survdat.compl <- data.frame(x)
colnames(survdat.compl) <- paste0("x", 1:10)
rate1 <- with(survdat.compl, exp(-
0.5+x1+0.4*x2+(x3==2)+0.5*(x4==2)+0.5*(x5==1)+rnorm(N,0,0.1))/10)
rate2 <- with(survdat.compl, exp(-1+0.1*x1+0.2*x2+(x3==2)+0.1*(x4==2)-
0.2*(x5==1)+rnorm(N,0,0.1))/10)

survdat.compl[,c(3:5, 8:10)] <- lapply(survdat.compl[,c(3:5, 8:10)],
factor)

fulltime <- rexp(N, rate = rate1)
fulltime2 <- rexp(N, rate = rate2)
censtimes <- 10 + 20*runif(N)

mintime <- pmin(fulltime, fulltime2, censtimes)
survdat.compl$time <- mintime

survdat.compl$status <- 0
survdat.compl$status[mintime == fulltime] <- 1
survdat.compl$status[mintime == fulltime2] <- 2

# introduce missing data for the covariates
```

```
missing.matrix=matrix(0, nrow=nrow(survdat.compl), ncol=10)
missing.matrix=matrix(rbinom(length(missing.matrix),1, p=0.02),
nrow=nrow(survdat.compl))
survdat=survdat.compl[,1:10]
survdat[missing.matrix==1]=NA
survdat=cbind(survdat, survdat.compl[11:12])

#create clusters
survdat$clust <- factor(sample(1:5, size = N, replace = TRUE, prob =
rep(0.2,5)))
survdat <- survdat[order(survdat$clust),]

#inspect data
head(survdat)
```

```
           x1          x2   x3 x4 x5         x6         x7 x8   x9 x10      time status clust
5  -1.7484193  0.18934122 <NA>  0  0  0.5400806  0.7311373  0    0   1  6.753887      2     1
7  -1.4796022 -1.30521258    0  0  0 -0.9412580 -1.0006861  0    0   2 19.014273      0     1
8  -1.4005888 -1.91554654    0  1  0  1.2988185  2.7307122  0    0   1  9.667323      1     1
10  1.5967150  1.47832616    0  0  0  0.3845286  1.0118199  0 <NA>   2  2.631073      1     1
11 -0.4144015  0.05737876    0  1  0 -2.0569517 -1.2557112  0    0   1  5.351348      2     1
12 -1.7079157 -1.26334587    0  0  0 -0.9881902 -2.2165987  0    0   2 23.622897      0     1
```

```
table(survdat$status)
```

```
  0   1   2
106 267 127
```

As we see, 106 of the patients did not have any event, 267 had event type 1, and 127 had event type 2.

5.2 *Perform multiple imputations*

We now use all predictors and auxiliary variables, status and log-time to perform the imputations. Here we use **`mice`**:[23]

```
# Impute missing data --------------
library(mice)
n.impute <- 10
survdat$logtime <- log(survdat$time)
meth <- make.method(survdat)
pred <- make.predictorMatrix(survdat)
pred[,c("time")] <- 0
imp.surv <- mice(survdat, pred = pred, meth = meth, m = n.impute)

# get imputed datasets
imputed <- list()
impc <- complete(imp.surv, action="long")
for(i in 1:n.impute){
  imputed[[i]] <- impc[impc$.imp==i, c(3:7, 13:15)]
}
```

5.3 *Visualize the data*

Here we use the **`cmprsk`** and **`survminer`** packages.[24,25]

```
library(cmprsk)
ci_fit <-
  cuminc(ftime = survdat.compl$time,fstatus=survdat.compl$status,cencode=0)
library(survminer)
ggcompetingrisks(ci_fit, xlab = "Days", conf.int = T, multiple_panels =
FALSE)
```

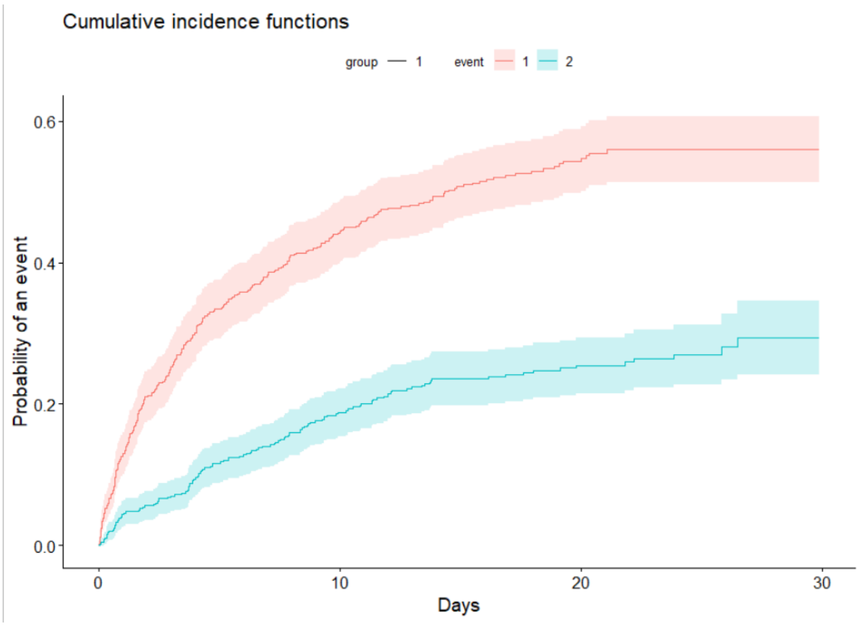

5.4 *Fit a prediction model in the imputed datasets*

Using the imputed datasets, we can fit a prediction model. In this example we use a cause-specific Cox proportional hazard regression model and **`riskRegression`**:[26]

```
# Fit cause-specific hazards models  ------
library(riskRegression)
fit.model <- list()
for(i in 1:n.impute){
  fit.model[[i]] <- CSC(
    formula = Hist(time, status) ~ x1 + x2 + x3 + x4 + x5,
    data = imputed[[i]]
  )
}
```

We can now use this model to predict the probability of each of the two outcomes for any time point:

```
 prediction.competing <- function(new.patient, single.fit = NULL,
                                  multiple.fit = NULL,
time.horizon = NULL, primary.event = NULL){

  if(!is.null(multiple.fit)){

    ff <- function(i){
      predictRisk(
        object = multiple.fit[[i]],
        cause = primary.event,
        newdata = new.patient,
        times = time.horizon
      )
    }
    prediction_matrix <- sapply(1:length(multiple.fit), ff)

    if(dim(new.patient)[1] == 1){
      prediction <- mean(prediction_matrix)
    } else{
      prediction <- apply(prediction_matrix, 1, mean)
    }
```

```
  } else if(!is.null(single.fit)){
    prediction <-
      predictRisk(
        object = single.fit,
        cause = primary.event,
        newdata = new.patient,
        times = time.horizon
      )
  }
  return(prediction)
}

time.horizon <- 10  ## time point for making predictions
new.patient <- data.frame(x1 = 1, x2 = -1.2, x3 = as.factor(1), x4 =
as.factor(0), x5=as.factor(0))
prediction.competing(new.patient, multiple.fit = fit.model, time.horizon =
time.horizon, primary.event = 1)
[1] 0.6059297
prediction.competing(new.patient, multiple.fit = fit.model, time.horizon =
time.horizon, primary.event = 2)
[1] 0.1649051
```

For this patient, the model estimates a 61% probability of experiencing event 1 and 16% probability of experiencing event 2 up until time=10.

We can also plot the predictions across all timepoints:

```
# plot survival curves
n.points=30
new.patient.prediction=data.frame(time=seq(0,30,length.out=n.points))

for (i in 1:n.points){
  new.patient.prediction$new.pred1[i]<-prediction.competing(new.patient,
multiple.fit = fit.model, time.horizon = new.patient.prediction$time[i],
primary.event = 1)
  new.patient.prediction$new.pred2[i]<-prediction.competing(new.patient,
multiple.fit = fit.model, time.horizon = new.patient.prediction$time[i],
primary.event = 2)

}
predictions.for.plot=data.frame(time=
  rep(new.patient.prediction$time,2),
                                pred=1-c(new.patient.prediction$new.pred1,
                                  new.patient.prediction$new.pred2 ),
                                event=c(rep("1",n.points),
rep("2",n.points)))

ggplot(predictions.for.plot, aes(x = time, y = pred, color = event)) +
geom_line(aes(group=factor(event)),size=1)
```

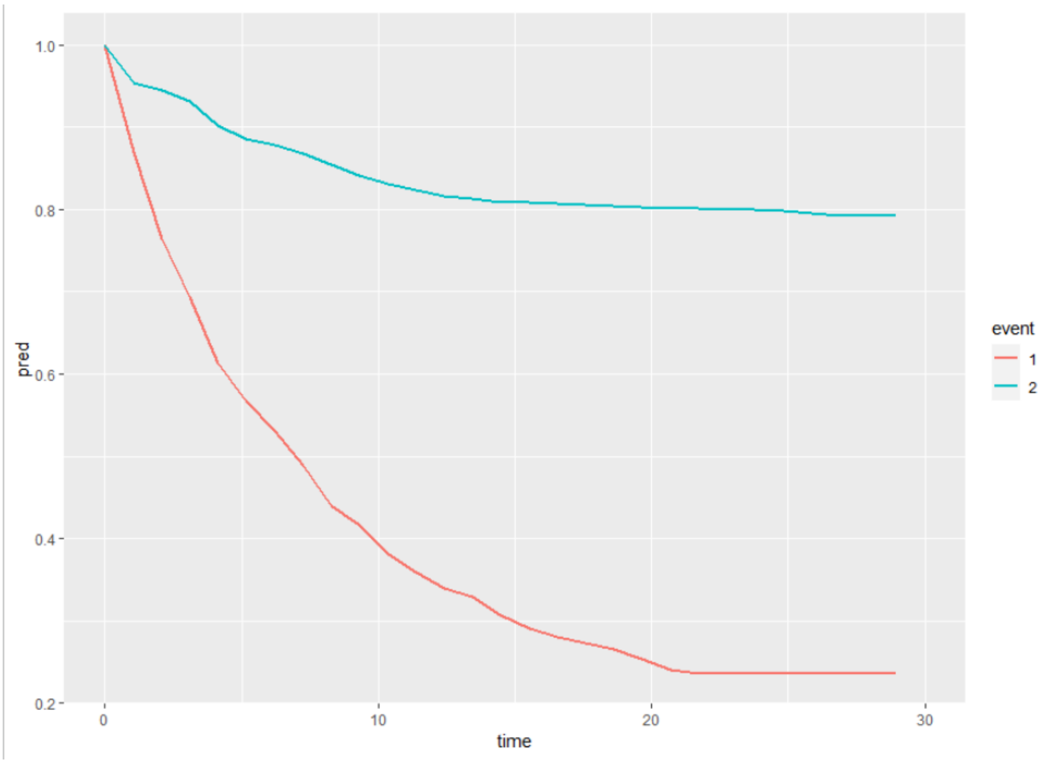

We will also predict survival for all patients with complete data. We will use this later to assess model's performance.

```
#predict for the complete dataset
complete.data <- survdat[complete.cases(survdat[,1:5]),]
predicted.competing.1 <- prediction.competing(complete.data, multiple.fit =
fit.model, time.horizon = time.horizon, primary.event = 1)
predicted.competing.2 <- prediction.competing(complete.data, multiple.fit =
fit.model, time.horizon = time.horizon, primary.event = 2)
```

5.5 *Calculate apparent performance of the model*

We can estimate model performance for each of the two competing events separately, starting by calibration, and observed vs. expected (OE) events ratio at a specific time point.

```
library(survival)
time.horizon<-10
# event 1
primary.event=1
complete.data$event <- factor(complete.data$status, 0:2, labels=c("censor",
"event", "competing"))

obj <- summary(survfit(Surv(time, event) ~ 1, data = complete.data), times
= time.horizon)
aj <- list("obs" = obj$pstate[, primary.event + 1], "se" = obj$std.err[,
primary.event + 1])

# Calculate O/E
OE <- aj$obs / mean(predicted.competing.1)
```

```
# For the confidence interval we use method proposed in Debray et al.
(2017) doi:10.1136/bmj.i6460
OE_summary1 <- c(
  "OE" = OE,
  "lower" = exp(log(OE - qnorm(0.975) * aj$se / aj$obs)),
  "upper" = exp(log(OE + qnorm(0.975) * aj$se / aj$obs))
)
round(OE_summary1,2)
   OE lower upper
 1.0   0.89  1.10
# event 2
primary.event=2
complete.data$event <- factor(complete.data$status, 0:2, labels=c("censor",
"event", "competing"))

obj <- summary(survfit(Surv(time, event) ~ 1, data = complete.data), times
= time.horizon)
aj <- list("obs" = obj$pstate[, primary.event + 1], "se" = obj$std.err[,
primary.event + 1])

# Calculate O/E
OE <- aj$obs / mean(predicted.competing.2)

# For the confidence interval we use method proposed in Debray et al.
(2017) doi:10.1136/bmj.i6460
OE_summary2 <- c(
  "OE" = OE,
  "lower" = exp(log(OE - qnorm(0.975) * aj$se / aj$obs)),
  "upper" = exp(log(OE + qnorm(0.975) * aj$se / aj$obs))
)
round(OE_summary2,2)
   OE lower upper
 0.97  0.78  1.17
```

We can also calculate AUC, c-index, and calibration intercept and slope, for each outcome. We use **geepack** and **pec**.[27,28] Below we only show for outcome 1:

```
library(geepack)
library(pec)

calculate_performance <- function(fit.model = NULL, time = NULL,
                                  status = NULL, data.used = NULL,
                                  time.horizon = NULL, primary.event =
NULL){

  score_vdata <- Score(
    list("csh_validation" = fit.model),
    formula = Hist(time, status) ~ 1,
    cens.model = "km",
    data = data.used,
    conf.int = TRUE,
    times = time.horizon,
    metrics = c("auc", "brier"),
    summary = c("ipa"),
    cause = primary.event,
    plots = "calibration" )

  pseudos <- data.frame(score_vdata$Calibration$plotframe)
  pseudos <- pseudos[order(pseudos$risk), ]
  pseudos$cll_pred <- log(-log(1 - pseudos$risk))
```

```
  # Fit model for calibration intercept
  fit_cal_int <- geese(
    pseudovalue ~ offset(cll_pred),
    data = pseudos,
    id = ID,
    scale.fix = TRUE,
    family = gaussian,
    mean.link = "cloglog",
    corstr = "independence",
    jack = TRUE  )

  # Fit model for calibration slope
  fit_cal_slope <- geese(
    pseudovalue ~ offset(cll_pred) + cll_pred,
    data = pseudos,
    id = ID,
    scale.fix = TRUE,
    family = gaussian,
    mean.link = "cloglog",
    corstr = "independence",
    jack = TRUE  )

  AUC <- score_vdata$AUC$score$AUC

  cindex_csh <- cindex(
    object = fit.model,
    formula = Hist(time, status) ~ 1,
    cause = primary.event,
    eval.times = time.horizon,
    data = data.used
  )$AppCindex$CauseSpecificCox

  returnVec <- c("calibration intercept" =
summary(fit_cal_int)$mean$estimate,
                 "calibration slope" = 1 +
summary(fit_cal_slope)$mean["cll_pred",]$estimate,
                 "AUC" = AUC,
                 "c-index" = cindex_csh)

  return(returnVec)
}

apparent.competing.list <- matrix(NA, nrow = n.impute, ncol = 4)
for(i in 1:n.impute){
apparent.competing.list[i,]<-
calculate_performance(fit.model=fit.model[[i]],
time=complete.data$time,
status=complete.data$status,
data.used=complete.data,
time.horizon=time.horizon,
primary.event=primary.event)}
apparent.competing1 <- apply(apparent.competing.list, 2, mean)
names(apparent.competing1)<-c("calibration intercept", "calibration slope",
"AUC", "c-index")
round(apparent.competing1,2)
```

```
calibration intercept     calibration slope                AUC              c-index
                 0.02                  1.01               0.83                 0.78
```

(see Section 5.8 on how to obtain uncertainty intervals around these numbers)

Next, we can create a calibration plot for each outcome. Below we only show for outcome 1.

```
#  calibration plot
primary.event<-1
pseudos=list()
smooth_pseudos=list()
for(i in 1:n.impute){
  score_vdata <- Score(
    list("csh_validation" = fit.model[[i]]),
    formula = Hist(time, status) ~ 1,
    cens.model = "km",
    data = complete.data,
    conf.int = TRUE,
    times = time.horizon,
    metrics = c("auc", "brier"),
    summary = c("ipa"),
    cause = primary.event,
    plots = "calibration"
  )
  pseudos[[i]] <- data.frame( score_vdata$Calibration$plotframe)
  pseudos[[i]] <- pseudos[[i]][order(pseudos[[i]]$risk), ]
  smooth_pseudos[[i]] <- predict(
    stats::loess(pseudovalue ~ risk, data = pseudos[[i]], degree = 1, span
= 0.33),se = TRUE)
}

ps.risk=pseudos[[1]]$risk/n.impute; for(i in
2:n.impute){ps.risk=ps.risk+pseudos[[i]]$risk/n.impute}
ps.val=pseudos[[1]]$pseudovalue/n.impute; for(i in
2:n.impute){ps.val=ps.val+pseudos[[i]]$pseudovalue/n.impute}
ps.fit=smooth_pseudos[[1]]$fit/n.impute; for(i in
2:n.impute){ps.fit=ps.fit+smooth_pseudos[[i]]$fit/n.impute}
ps.df=smooth_pseudos[[1]]$df/n.impute; for(i in
2:n.impute){ps.df=ps.df+smooth_pseudos[[i]]$df/n.impute}
ps.se=smooth_pseudos[[1]]$se/n.impute;for(i in
2:n.impute){ps.se=ps.se+smooth_pseudos[[i]]$se/n.impute}

spike_bounds <- c(-0.075, 0)
bin_breaks <- seq(0, 0.6, length.out = 100 + 1)
freqs <- table(cut(predicted.competing.1, breaks = bin_breaks))
bins <- bin_breaks[-1]
freqs_valid <- freqs[freqs > 0]
freqs_rescaled <- spike_bounds[1] + (spike_bounds[2] - spike_bounds[1]) *
  (freqs_valid - min(freqs_valid)) / (max(freqs_valid) - min(freqs_valid))


# produce plot
plot(
  x = ps.risk,
  y = ps.val,
  xlim = c(0, 0.6),
  ylim = c(spike_bounds[1], 0.6),
  yaxt = "n",
  frame.plot = FALSE,
  xlab = "Estimated risks",
  ylab = "Observed outcome proportions",
  type = "n"
)
axis(2, seq(0, 0.6, by = 0.1), labels = seq(0, 0.6, by = 0.1))
polygon(
```

```
  x = c(ps.risk, rev(ps.risk)),
  y = c(
    pmax(ps.fit - qt(0.975, ps.df) * ps.se, 0),
    rev(ps.fit + qt(0.975, ps.df) * ps.se)
  ),
  border = FALSE,
  col = "lightgray"
)
abline(a = 0, b = 1, col = "gray")
lines(x = ps.risk, y = ps.fit, lwd = 2)
segments(
  x0 = bins[freqs > 0],
  y0 = spike_bounds[1],
  x1 = bins[freqs > 0],
  y1 = freqs_rescaled
)
```

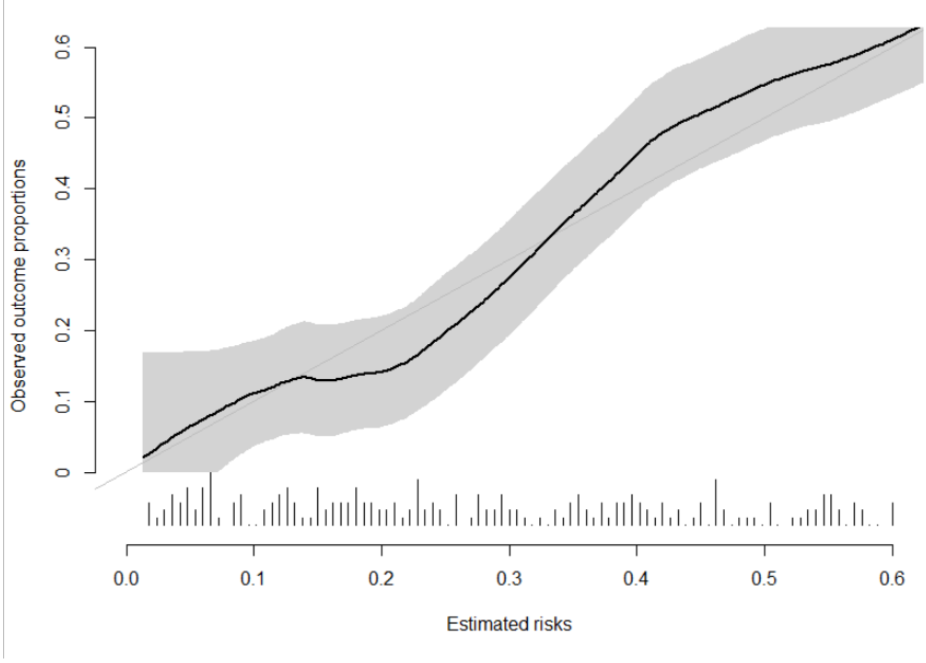

5.6 Calculate optimism-corrected performance of the model

We can now use bootstrapping to assess model optimism.

```
# Internal CV via bootstrapping -------------------
# bootstrap in each multiply imputed dataset
n.bootstrap <- 10
n.impute <- 10
primary.event<-1
time.horizon<-10
optimism.competing.eachbootstrap <-  matrix(NA, n.bootstrap, 4)
optimism.competing <- matrix(NA, n.impute, 4)

for (i in 1:n.impute){
  for(j in 1:n.bootstrap){

    boot.sample <- sample(length(imputed[[i]]$time), replace = T)

    # create bootstrap sample
```

```
    imp.boot <- as.data.frame(lapply(imputed[[i]],
function(x){x[boot.sample]}))

    # predict in bootstrap
    f1 <- as.data.frame(do.call(cbind, lapply(imp.boot, function(x)
{as.numeric(as.character(x))})))

    # fit cause specific hazard model
    fit.competing.boot <- CSC(formula = Hist(time, status) ~ x1 + x2 + x3 +
x4,
                              data = f1)

    competing.boot <- calculate_performance(fit.model = fit.competing.boot,
                                            time = f1$time,
                                            status = f1$status,
                                            data.used = f1,
                                            time.horizon = time.horizon,
                                            primary.event = primary.event)

    # predict in test data
    f2 <- as.data.frame(do.call(cbind, lapply(imputed[[i]], function(x)
{as.numeric(as.character(x))})))

    competing.test <- calculate_performance(fit.model = fit.competing.boot,
                                            time = f2$time,
                                            status = f2$status,
                                            data.used = f2,
                                            time.horizon = time.horizon,
                                            primary.event = primary.event)

    optimism.competing.eachbootstrap[j,] <- competing.boot - competing.test
  }
  optimism.competing[i,] <- apply(optimism.competing.eachbootstrap, 2,
mean)

  print(paste0("imputation done: ", i))
}

mean.optimism.competing <- apply(optimism.competing, 2, mean)
optimism.corrected.competing <- apparent.competing1 -
mean.optimism.competing
round(apparent.competing1,2)
calibration intercept     calibration slope                   AUC               c-index
                0.016                 1.008                 0.834                 0.778
round(optimism.corrected.competing,2)
calibration intercept     calibration slope                   AUC               c-index
                0.020                 0.974                 0.828                 0.772
```

Essentially we see no optimism here, which is of course expected due to the large sample size and small size of covariates used in the model.

5.7 Perform internal-external cross-validation

We can follow an internal-external cross-validation using the `clust` variable in the dataset.

```
# Internal-external CV -------------------
clusters <- unique(survdat$clust)
N.clust <- length(clusters) # 10 clusters in this example
data.in <- data.leftout <- list()

#create the datasets
```

```
for(i in 1:N.clust){
  data.in[[i]]<- survdat[survdat$clust!=clusters[i],]
  data.leftout[[i]]<- survdat[survdat$clust==clusters[i],]
  complete.index <- complete.cases(data.leftout[[i]][,c(paste0("x", 1:5))])
  data.leftout[[i]] <- data.leftout[[i]][complete.index,]
}

n.impute <- 10
imputed <- fit.competing.CV <- list()
leftout.prediction.competing <- leftout.performance.competing <- list()

for (i in 1:N.clust){
  data.in[[i]]$logtime <- log(data.in[[i]]$time)
  meth <- make.method(data.in[[i]])
  pred <- make.predictorMatrix(data.in[[i]])
  pred[,c("time")] <- 0
  imp.surv <- mice(data.in[[i]], pred = pred, meth = meth, m = n.impute)

  # get imputed datasets
  imputed <- list()
  impc <- complete(imp.surv, action="long")
  for(j in 1:n.impute){
    imputed[[j]] <- impc[impc$.imp==j, c(3:7, 13:15)]
  }

  leftout.performance.competing.each <- matrix(NA, nrow = n.impute, ncol =
4)
  for(j in 1:n.impute){
    fit.competing.CV <- CSC(
      formula = Hist(time, status) ~ x1 + x2 + x3 + x4,
      data = imputed[[j]]
    )

    leftout.performance.competing.each[j,] <- calculate_performance(
                          fit.model = fit.competing.CV,
                          time = data.leftout$time,
                          status = data.leftout$status,
                          data.used = imputed[[j]],
                          time.horizon = time.horizon,
                          primary.event = primary.event)
  }
  leftout.performance.competing[[i]] <-
apply(leftout.performance.competing.each, 2, mean)
}

# performance per cluster
leftout.performance.competing
int.ext.competing<-
data.frame(cluster=1:N.clust,round(matrix(unlist(leftout.performance.compet
ing),nrow=N.clust, byrow = T),2))
colnames(int.ext.competing)=c("cluster","calibration intercept",
"calibration slope", "AUC", "c-index")
int.ext.competing
  cluster calibration intercept calibration slope  AUC c-index
1       1                  0.04              0.97 0.82    0.76
2       2                  0.07              1.01 0.83    0.77
3       3                  0.02              0.94 0.81    0.76
4       4                  0.01              1.02 0.84    0.78
5       5                  0.01              0.93 0.81    0.76
```

We see very small variation across clusters.

5.8 *Obtain uncertainty intervals for performance measures*

Finally, we can repeat the calculations of Section 5.5 to also obtain some uncertainty intervals.

```
### calculate CIs for apparent performance -------
library(geepack)
library(pec)

calculate_performance <- function(fitmodel = NULL, time = NULL,
                                  status = NULL, data.used = NULL,
                                  time.horizon = NULL, primary.event =
NULL, bootstrap=100){

  score_vdata <- Score(
    list("csh_validation" = fitmodel),
    formula = Hist(time, status) ~ 1,
    cens.model = "km",
    data = data.used,
    conf.int = TRUE,
    times = time.horizon,
    metrics = c("auc", "brier"),
    summary = c("ipa"),
    cause = primary.event,
    plots = "calibration" )

  pseudos <- data.frame(score_vdata$Calibration$plotframe)
  pseudos <- pseudos[order(pseudos$risk), ]
  pseudos$cll_pred <- log(-log(1 - pseudos$risk))

  # Fit model for calibration intercept
  fit_cal_int <- geese(
    pseudovalue ~ offset(cll_pred),
    data = pseudos,
    id = ID,
    scale.fix = TRUE,
    family = gaussian,
    mean.link = "cloglog",
    corstr = "independence",
    jack = TRUE  )

  # Fit model for calibration slope
  fit_cal_slope <- geese(
    pseudovalue ~ offset(cll_pred) + cll_pred,
    data = pseudos,
    id = ID,
    scale.fix = TRUE,
    family = gaussian,
    mean.link = "cloglog",
    corstr = "independence",
    jack = TRUE  )

  AUC <- score_vdata$AUC$score$AUC
  se.AUC <- score_vdata$AUC$score$se

  ### CI bootstraps
  boots_ls <- lapply(seq_len(bootstrap), function(b) {
    vdata_boot <- data.used[sample(nrow(data.used), replace = TRUE), ]
    cindex_boot <- pec::cindex(
      object = fitmodel,
      formula = Hist(time, status) ~ 1,
```

```
      cause = primary.event,
      eval.times = time.horizon,
      data = vdata_boot,
      verbose = FALSE
    )$AppCindex$CauseSpecificCox


    cbind.data.frame("cindex" = cindex_boot)
  })

  cindex <- do.call(rbind.data.frame, boots_ls)

  #####
  returnVec <- c("cal.int" = summary(fit_cal_int)$mean$estimate,
                 "cal.int.se"=summary(fit_cal_int)$mean$san.se,
                 "cal.slope" = 1 +
summary(fit_cal_slope)$mean["cll_pred",]$estimate,

"cal.slope.se"=summary(fit_cal_slope)$mean["cll_pred",]$san.se,
                 "AUC" = AUC,"se.AUC"=se.AUC,
                 "c.index" = cindex)

  return(returnVec)
}

apparent.competing.list <- list()
cindex=c(); auc=c(); se.auc=c();
cal.int=c();cal.int.se=c();cal.slope=c();cal.slope.se=c()

for(i in 1:n.impute){
apparent.competing.list[[i]]<-
calculate_performance(fitmodel=fit.model[[i]],
time=complete.data$time,
status=complete.data$status,
data.used=complete.data,
time.horizon=time.horizon,
primary.event=1,
bootstrap=10)
  cindex=c(cindex, apparent.competing.list[[i]]$c.index)
  auc=c(auc,apparent.competing.list[[i]]$AUC )
  se.auc=c(se.auc, apparent.competing.list[[i]]$se.AUC)
  cal.int=c(cal.int,apparent.competing.list[[i]]$cal.int)
  cal.int.se=c(cal.int.se,apparent.competing.list[[i]]$cal.int.se)
  cal.slope=c(cal.slope,apparent.competing.list[[i]]$cal.slope)
  cal.slope.se=c(cal.slope.se,apparent.competing.list[[i]]$cal.slope.se)
}

apparent.cindex=round(c(mean(cindex), quantile(cindex, probs = c(0.025,
0.975)) ),2)
apparent.se.auc=sqrt(mean(se.auc^2)+(1+1/n.impute)/n.impute*(sum((mean(auc)
-auc)^2)))
apparent.AUC=round(c(mean(auc), mean(auc)-1.96*apparent.se.auc,
mean(auc)+1.96*apparent.se.auc),2)
names(apparent.AUC)=c("mean", "2.5%", "97.5%")

apparent.se.int=sqrt(mean(cal.int.se^2)+(1+1/n.impute)/n.impute*(sum((mean(
cal.int)-cal.int)^2)))
apparent.se.slope=sqrt(mean(cal.slope.se^2)+(1+1/n.impute)/n.impute*(sum((m
ean(cal.slope)-cal.slope)^2)))
```

```
apparent.int=round(c(mean(cal.int), mean(cal.int)-1.96*apparent.se.int,
mean(cal.int)+1.96*apparent.se.int),2)
names(apparent.int)=c("mean", "2.5%", "97.5%")

apparent.slope=round(c(mean(cal.slope), mean(cal.slope)-
1.96*apparent.se.slope, mean(cal.slope)+1.96*apparent.se.slope),2)
names(apparent.slope)=c("mean", "2.5%", "97.5%")

# results
apparent.cindex
       2.5% 97.5%
 0.78  0.74  0.81
apparent.AUC
 mean  2.5% 97.5%
 0.83  0.80  0.87
apparent.int
 mean  2.5% 97.5%
 0.02 -0.15  0.18
apparent.slope
 mean  2.5% 97.5%
 1.01  0.80  1.21
```

5.9 *Decision curve analysis*

Here we first need to provide the primary event of interest and a time point.

```
### Decision curve analysis
primary.event<-1
time.of.interest<-15
predicted.for.dca1 <- prediction.competing(complete.data, multiple.fit =
fit.model,time.horizon = time.of.interest, primary.event = 1)

for.dca=data.frame(time=complete.data$time, status=complete.data$status,
pred=predicted.for.dca1)
for.dca$status[for.dca$status!=primary.event]=0
dca1<-dca(Surv(time, status) ~ pred,
          data = for.dca,thresholds = 1:100 / 100,
          time = time.horizon)
plot(dca1,smooth = TRUE)
```

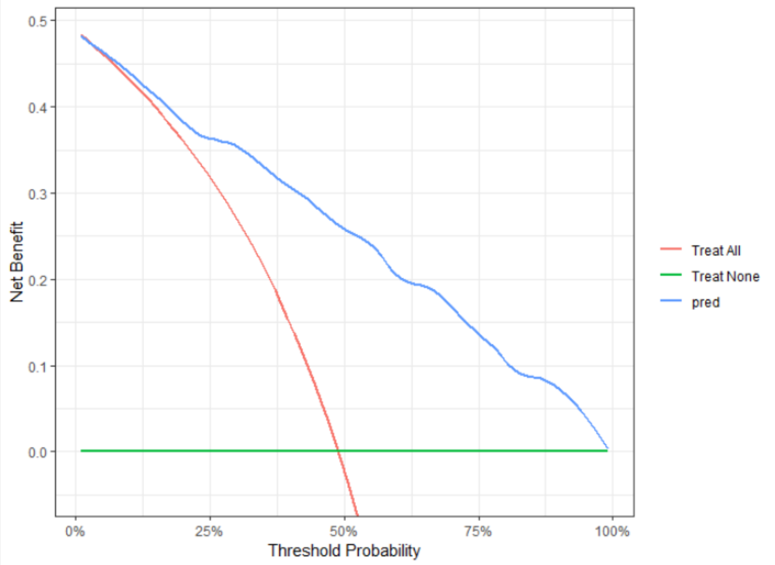

We see that the model performs better than both treat all and treat none strategies for thresholds above 5%.

6 References

1. Ensor, J., Martin, E. C. & Riley, R. D. pmsampsize: Calculates the Minimum Sample Size Required for Developing a Multivariable Prediction Model. (2021).
2. Ripley, B. *et al.* MASS: Support Functions and Datasets for Venables and Ripley's MASS. (2022).
3. Wickham, H. *et al.* ggplot2: Create Elegant Data Visualisations Using the Grammar of Graphics. (2022).
4. Harrell, F. J. & Dupont, C. Hmisc: Harrell Miscellaneous. (2021).
5. Harrell, F. rms: Regression Modeling Strategies. R package version 6.0-1. https://CRAN.R-project.org/package=rms. (2021).
6. Auguie, B. & Antonov, A. gridExtra: Miscellaneous Functions for 'Grid' Graphics. (2017).
7. Wood, S. mgcv: Mixed GAM Computation Vehicle with Automatic Smoothness Estimation. (2021).
8. Friedman, J. *et al.* glmnet: Lasso and Elastic-Net Regularized Generalized Linear Models. (2021).
9. Wang [aut, W., cre & Yan, J. splines2: Regression Spline Functions and Classes. (2022).
10. Kassambara, A. ggpubr: 'ggplot2' Based Publication Ready Plots. (2020).
11. Robin, X. *et al.* pROC: an open-source package for R and S+ to analyze and compare ROC curves. *BMC Bioinformatics* **12**, 77 (2011).
12. Schwarzer, G. meta: General Package for Meta-Analysis. (2018).
13. Sjoberg, D. D. & Vertosick, E. dcurves: Decision Curve Analysis for Model Evaluation. (2022).
14. Vickers, A. J., Cronin, A. M., Elkin, E. B. & Gonen, M. Extensions to decision curve analysis, a novel method for evaluating diagnostic tests, prediction models and molecular markers. *BMC Medical Informatics and Decision Making* **8**, 53 (2008).
15. Zhang, Z. *et al.* Decision curve analysis: a technical note. *Annals of Translational Medicine* **6**, 19–19 (2018).
16. Horikoshi, M. *et al.* ggfortify: Data Visualization Tools for Statistical Analysis Results. (2022).
17. Therneau, T. M., until 2009), T. L. (original S.->R port and R. maintainer, Elizabeth, A. & Cynthia, C. survival: Survival Analysis. (2022).
18. White, I. R. & Royston, P. Imputing missing covariate values for the Cox model. *Stat Med* **28**, 1982–1998 (2009).
19. Harrell, F. E., Califf, R. M., Pryor, D. B., Lee, K. L. & Rosati, R. A. Evaluating the yield of medical tests. *JAMA* **247**, 2543–2546 (1982).
20. Uno, H., Cai, T., Pencina, M. J., D'Agostino, R. B. & Wei, L. J. On the C-statistics for Evaluating Overall Adequacy of Risk Prediction Procedures with Censored Survival Data. *Stat Med* **30**, 1105–1117 (2011).
21. McLernon, D. J. *et al.* Assessing performance and clinical usefulness in prediction models with survival outcomes: practical guidance for Cox proportional hazards models. 2022.03.17.22272411 Preprint at https://doi.org/10.1101/2022.03.17.22272411 (2022).

22. Geloven, N. van *et al.* Validation of prediction models in the presence of competing risks: a guide through modern methods. *BMJ* **377**, e069249 (2022).
23. Buuren, S. van *et al.* mice: Multivariate Imputation by Chained Equations. (2021).
24. Gray, B. cmprsk: Subdistribution Analysis of Competing Risks. (2022).
25. Kassambara, A., Kosinski, M., Biecek, P. & Fabian, S. survminer: Drawing Survival Curves using ‘ggplot2’. (2021).
26. Gerds, T. A. *et al.* riskRegression: Risk Regression Models and Prediction Scores for Survival Analysis with Competing Risks. (2022).
27. Højsgaard, S., Halekoh, U., Yan, J. & Ekstrøm, C. T. geepack: Generalized Estimating Equation Package. (2022).
28. Gerds, T. A. pec: Prediction Error Curves for Risk Prediction Models in Survival Analysis. (2022).
